# Gastrodinol derivatives and prenylated flavones from the flower branch of *Gastrodia* *elata*

**DOI:** 10.1007/s13659-024-00430-6

**Published:** 2024-03-20

**Authors:** Shi-Hui Qin, Zhi-Lan Li, Liu Yang, Jiang-Miao Hu

**Affiliations:** 1 State Key Laboratory of Phytochemistry and Plant Resources in West China, Kunming Institute of Botany, Chinese Academy of Sciences, Kunming, 650201 Yunnan China; 2 College of Pharmacy, Anhui University of Chinese Medicine, Hefei, 230012 China

**Keywords:** *Gastrodiaelata*, Orchidaceae, Gastrodinol, Isoprene-flavonoids, Mulberrofurans, Gastrodiamide

## Abstract

Based on the research progress and traditional usage with whole herbal of the TCM "Tianma", chemical studies herein on the flower branch of *Gastrodia* *elata* were carried out in-depth and got 13 compounds including the gastrodinols (**1–4**), the flavonoid morins (**5–8, 11–12**), together with the specialist mulberrofurans (**9**, **13**) and gastrodiamide (**10**) for the first time from the species. The antibacterial and cholinesterase inhibitory activities were then evaluated and the results showed that compounds** 5**, **11**, **12**, **13** have good activity against anti-methicillin-resistant *Staphylococcus* *aureus*, and compounds **9**, **13** had good acetylcholinesterase inhibitory activity. All these results provide new chemical composition for better understanding the traditional application of "Tianma" and for exploring new pharmacological ingredients.

**Graphical Abstract:**

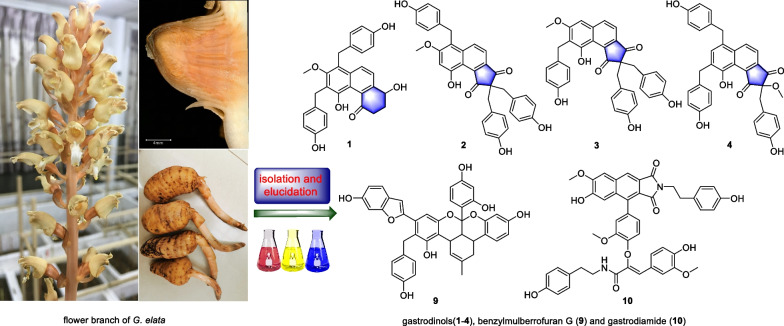

**Supplementary Information:**

The online version contains supplementary material available at 10.1007/s13659-024-00430-6.

## Introduction

**Fig. 1:**
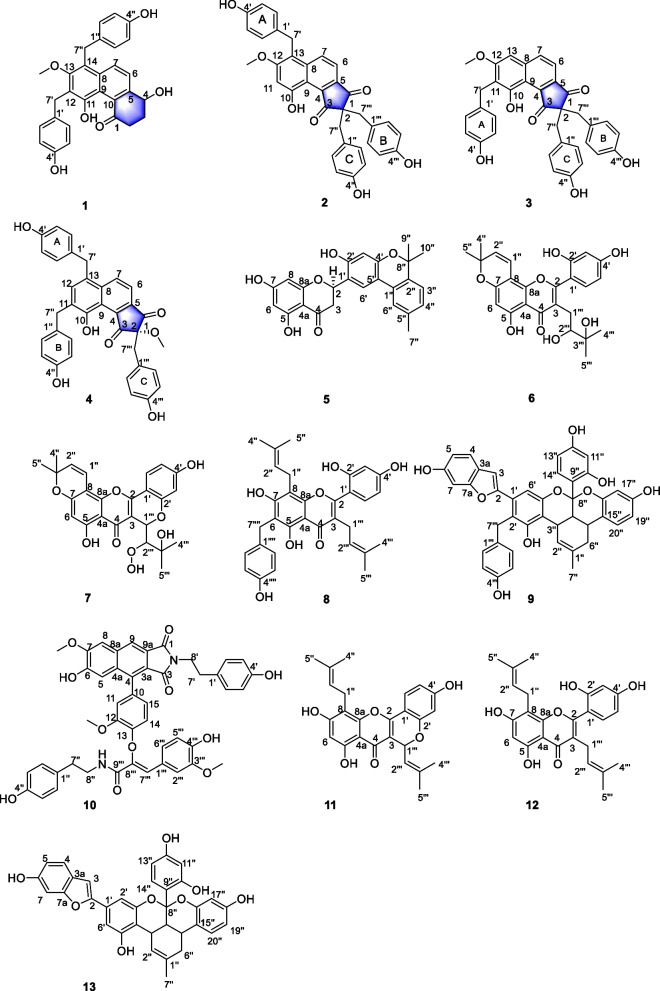
The structures of compounds **1**–**13**

*Gastrodia* *elata* used for "Tianma" is a perennial herb of the genus *Gastrodia* in Orhcidaceae family and mainly distributed in Sichuan, Yunnan, Guizhou, Shanxi, Anhui, Jilin and other places in China [1]. Since the 1960s, Prof Zhou Jun et al. have successively isolated and elucidated the main chemical composition includes gastrodin and parishins etc., and has made progress in the clinical application of gastrodin [2]. In order to further understand the active ingredients of the TCM "Tianma" and explore new drug precursors as mentioned ever, Prof Zhou’s group got gastrodinol etc. from the flower branch of *G.* *elata* with good antibacterial activities [3–5]. With the further pharmaceutical research of the TCM, the ethyl acetate of aboveground of the species was studied herein again to obtain 10 new compounds named as isogastrodinol (**1**), gastrodinols B-D (**2–4**), isotetrapterol A (**5**), morusinol B (**6**), cyclomorusinol hydroperoxide (**7**), benzylkuwanon C (**8**), benzylmulberrofuran G (**9**), gastrodiamide (**10**), together with three known compounds including cyclomulberrin (**11**) [6], kuwanon C (**12**) [7] and mulberrofuran G (**13**) [8] (Fig. 1).

Antimicrobial resistance in bacterial pathogens is a global challenge associated with high morbidity and mortality in pathogens resistant to existing antibiotics, and the threat of infectious bacteria is gradually increasing [9, 10]. Traditional medicinal plants have a variety of components, which can treat a variety of diseases caused by bacteria and have the advantages of less side effects. On this basis and from the early traditional usage of the TCM "Tianma", some of the compounds isolated herein were screened for anti-MRSA activity. The results showed that compounds **5**, **11**, **12** and **13** had good anti-MRSA activities, and their MIC_50_ were 4.46 μM, 1.908 μM, 1.911 μM and 1.264 μM, respectively. At the same time, compounds** 2**, **3**, **9** and **13** were sent for screening and showed good acetylcholinesterase inhibition activity with IC_50_ values of 24.682 μM, 15.663 μM, 0.488 μM and 1.057 μM, respectively.

## Results and discussion

### Structure determination

**Fig. 2:**
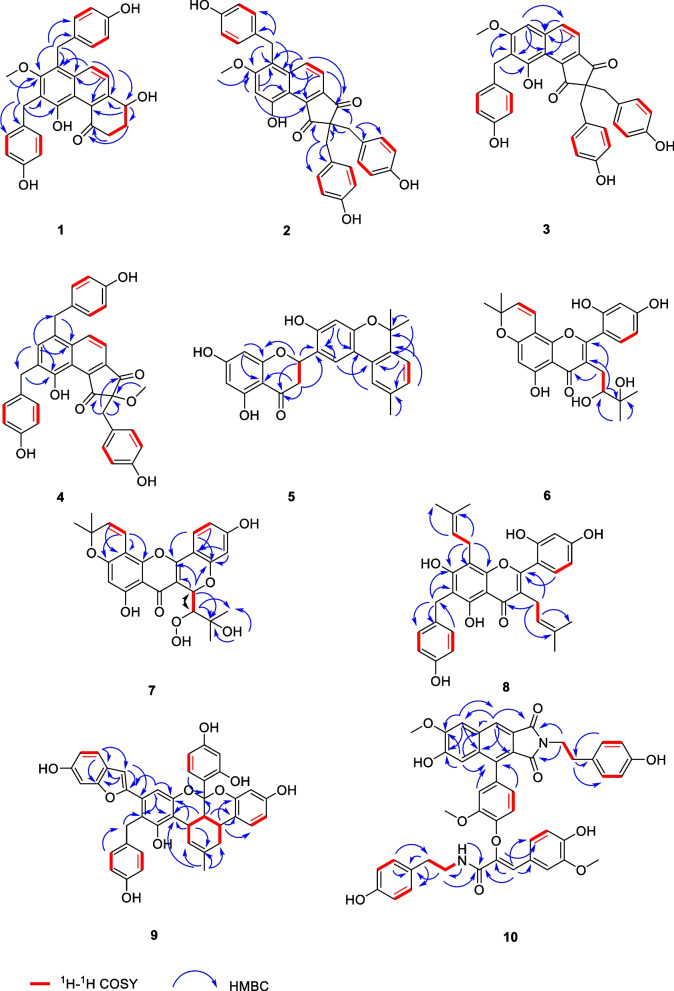
Selected ^1^H–^1^H COSY and HMBC correlations of compounds **1**–**10**

Compound **1** was obtained as yellow amorphous powder. The molecular formula C_29_H_26_O_6_ was established by HRESI-MS at *m/z* 469.1662 [M–H] ^−^, corresponding to 17 degrees of unsaturation. The IR spectrum of **1** exhibited typical absorptions of a hydroxyl (3413 cm^−1^), carbonyl (1631 cm^−1^) and a benzene ring (1594 cm^−1^). The ^13^C NMR, DEPT and HSQC spectra of **1** (Table 1) showed 29 carbon signals, including one carbonyl, twenty-two aromatic carbons, one oxymethine, four methylenes, and one methoxy, thus it is inferred that the compound contains four benzene rings including a naphthalene rings. Combining the NMR data of gastrodinol [3, 4] and bulbocodin C [11], the ^1^H-^1^H COSY correlations of H2/H3/H4 and H6/H7, the HMBC (Fig. 2) correlations of H-6/C-4, C-6 and C-8, H-4/C-3, C-5 and C-10 in compound **1** confirmed a fragment of the naphthalene ring with two *p*-hydroxybenzyl fragments. The HMBC correlations of H-7′/C-11, C-12, C-13 and H-7′′/C-8, C-13, C-14 supported that *p*-hydroxybenzyl was linked to C-12 and C-14, respectively. On the basis of above evidences, the structure of **1** was elucidated as 1,5-dihydroxy-6,8-*bis*(4-hydroxybenzyl)-7-methoxy-2,3-dihydrophenanthren-4(1*H*)-one, named as isogastrodinol.

**Table 1:** ^1^H NMR and ^13^C NMR spectroscopic date for compounds **1**–**4**

No.	1^a^		2^a^		3^a^		4^b^	
	*δ*_C_	*δ*_H_, m, *J* (Hz)	*δ*_C_	*δ*_H_, m, *J* (Hz)	*δ*_C_	*δ*_H_, m, *J* (Hz)	*δ*_C_	*δ*_H_, m, *J* (Hz)
1	206.61	–	200.57	–	203.22	–	200.21	–
2	37.31	3.11, m 2.84, m	61.84	–	63.89	–	86.86	–
3	30.87	2.37, m 2.16, m	210.38	–	211.92	–	208.74	–
4	69.51	4.93, dd, 7.9, 4.0	140.34	–	141.66	–	140.57	–
5	151.30	–	142.19	–	144.67	–	144.59	–
6	125.34	7.59, d, 8.9	117.65	7.50, d, 9.0	118.03	7.43, d, 8.5	117.25	7.56, d, 8.8
7	134.78	8.20, d, 8.9	135.46	8.21, d, 9.0	139.45	7.93, dd, 8.5, 1.6	138.49	8.39, d, 8.8
8	134.57	–	136.80	–	139.67	–	136.98	–
9	121.45	–	112.56	–	115.37	–	120.16	–
10	129.09	–	155.32	–	154.56	–	152.62	–
11	152.70	–	101.84	7.02, s	120.59	–	130.06	–
12	125.47	–	159.72	–	162.13	–	137.39	7.41, s
13-OCH_3_	62.30	3.59, s	–	–		–		
13	158.23	–	115.06	–	100.75	6.89, brs	130.06	–
14	121.63	–	–	–				
1′	133.54	–	130.49	–	133.10	–	132.62	–
2′, 6′	130.29	7.08, d, 8.6	128.72	6.80, d, 8.6	130.60	7.09, d, 8.6	130.28	6.81, d, 8.1
3′, 5′	115.97	6.63, d, 8.6	114.97	6.55, d, 8.6	115.80	6.65, d, 8.6	116.32	6.63, d, 8.1
4′	156.26	–	155.40	–	156.20	–	156.66	–
7′	31.30	4.20, s	28.73	4.10, s	29.32	4.05, s	38.78	4.20, m
1′′	133.38	–	125.15	–	127.45	–	132.78	–
2′′, 6′′	129.83	6.84, d, 8.6	130.44	6.71, d, 8.5	131.81	6.80, d, 8.6	130.94	7.08, d, 8.1
3′′, 5′′	115.64	6.60, d, 8.6	115.16	6.38, d, 8.5	115.89	6.38, d, 8.6	116.20	6.69, d, 8.1
4′′	155.98	–	155.98	–	157.27	–	156.73	–
7′′	31.15	4.39, s	39.96	3.14, m	41.76	3.21, s	36.20	4.07, m
12-OCH_3_	–	–	56.35	3.92, s	56.31	3.89, s		
10-OH	–	–	–	12.11, s	–	–	–	–
4′-OH	–	–	–	9.12, s	–	–	–	–
4′′-OH	–	–	–	9.12, s	–	–	–	–
4′′′-OH	–	–	–	9.12, s	–	–	–	–
1′′′	–	–	–	–	127.45	–	124.73	–
2′′′, 6′′′	–	–	–	–	131.81	6.80, d, 8.6	131.82	6.70, d, 8.0
3′′′, 5′′′	–	–	–	–	115.89	6.38, d, 8.6	116.13	6.32, d, 8.0
4′′′	–	–	–	–	157.27	–	157.71	–
7′′′	–	–	–	–	41.76	3.21, s	42.52	3.20, s
2-OCH_3_	–	–	–	–	–	–	54.66	3.27, s

^a1^H NMR recorded at 500 MHz, ^13^C NMR recorded at 125 MHz in Methanol-*d*_4_

^b1^H NMR recorded at 800 MHz, ^13^C NMR recorded at 200 MHz in Methanol-*d*_4_

Compound **2** was isolated as a yellow amorphous powder. It was established by the HRESI-MS ion peak at *m/z* 673.1695 ([M + CF_3_COO] ^−^, calcd for 673.1691), which suggested a molecular formula of C_35_H_28_O_7_ with 22 degrees of unsaturation. The IR spectrum showed hydroxyl (3395 cm^−1^), carbonyl (1719 cm^−1^, 1669 cm^−1^) and a benzene ring (1612 cm^−1^) absorption signals and thus strongly indicated the presence of a phenolic structure. The ^1^H NMR spectrum data of compound **2** (Table 1) displayed that the compound had three 1,4-disubstituted benzene ring fragments [*δ*_H_ 6.80 (2H, d, *J* = 8.6 Hz, H-2′, H-6′), 6.71 (4H, d, *J* = 8.5 Hz, H-2′′, H-6′′, H-2′′′, H-6′′′), 6.55 (2H, d, *J* = 8.6 Hz, H-3′, H-5′), 6.38 (4H, d, *J* = 8.5 Hz, H-3′′, H-5′′, H-3′′′, H-5′′′)] together with three methylene signals [*δ*_H_ 4.10 (2H, s, H-7′), 3.14 (4H, m, H-7′′, H-7′′′)]. Combining the 1D and 2D NMR data of **2 ** (Table 1) with that of gastrodinol [3], compound **2** has a similar nuclear structure but with three *p*-hydroxybenzyl fragments. The HMBC correlations of (Fig. 2) H-7′/C-8, C-12 and C-13 supported that a *p*-hydroxybenzyl was linked to C-13. Therefore, compound **2** was elucidated as 9-hydroxy-2,2,6-tris(4-hydroxybenzyl)-7-methoxy-1*H*-cyclopenta[*a*]naphthalene-1,3(2*H*)-dione, named as gastrodinol B.

Compound **3** was acquired as yellow amorphous powder, and its molecular formula of C_35_H_28_O_7_ determined by an HRESI-MS ion at *m/z* 605.1812 [M + COOH] ^–^ (calcd for 605.1817), corresponding to 22 degrees of unsaturation. The IR absorption bands at 3432 and 1713 cm^−1^ suggested the presence of hydroxyl and carbonyl. The ^1^H NMR and ^13^C NMR data were similar to those of **2**, while the HMBC spectrum (Fig. 2) correlations of H-7/C-8, C-9 and C-13; H-13/C-7, C-8, C-9 and C-11; H-7′/C-10, C-11 and C-12 could speculated that the *p*-hydroxybenzyl fragment substituted at C-11 but not C-13 in compound **3**. Subsequently, compound **3** was identified as 9-hydroxy-2,2,8-tris(4-hydroxybenzyl)-7-methoxy-1*H*-cyclopenta[*a*]naphthalene-1,3(2*H*)-dione, named as gastrodinol C.

**Fig. 3:**
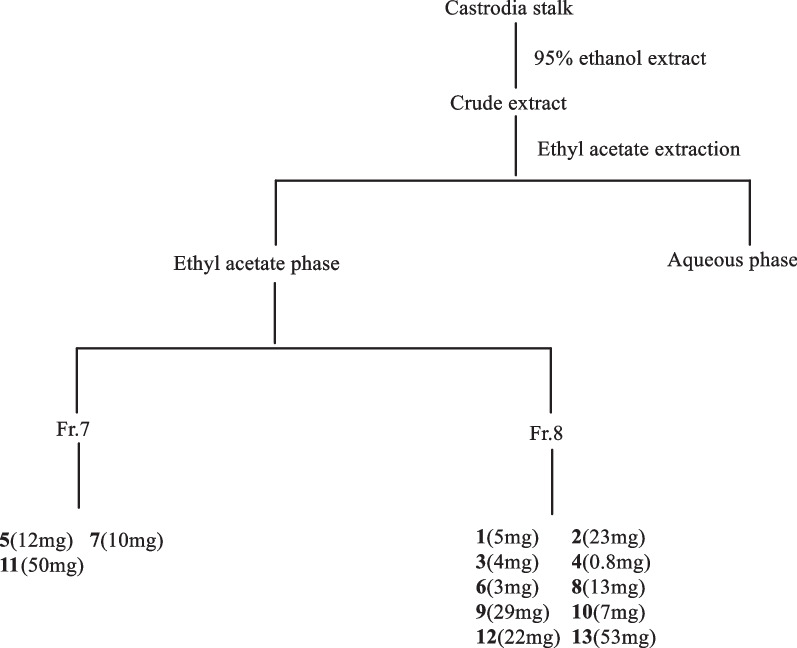
Calculated and experimental ECD spectra of compounds **4** and **5**

Compound **4** was obtained as yellow amorphous powder, having the molecular formula of C_35_H_28_O_7_ from HRESIMS (*m/z* 673.1685 [M + CF_3_COO] ^−^, calcd for 673.1691) with 22 degrees of unsaturation. The IR spectrum indicated the existence of hydroxyl (3425 cm^−1^) and carbonyl (1732 cm^−1^, 1679 cm^−1^). Comparing the ^1^H NMR and ^13^C NMR data (Table 1) of **4** with that of gastrodinol [3] and compound **2**, it could be seen that three *p*-hydroxybenzyl fragments were linked to C-13, C-11 and C-2, respectively. The correlations in HMBC spectra (Fig. 2) confirmed this linkage further with that of H-12/C-8, C-7′ and H-7′/C-8 (C-13); H-12/C-10, C-7′′ and H-7′′/C-10 (C-11); H-7′′′/C-1, C-2 and C-3 (C-2). In addition, in order to determine the absolute configuration of the compound, the ECD profiles of the calculated configuration of the compound were simulated using SpecDis1.70 software, and then compared with the experimental ECD profiles. The calculated results show that the experimental ECD spectra of compound **4** were basically in complete agreement with the computed spectra of *S* (4), and the absolute configuration of compound **4** was determined as 2S (Fig. 3a). Thus, the structure of **4** was determined as 9-hydroxy-2,6,8-tris(4-hydroxybenzyl)-2-methoxy-1*H*-cyclopenta[*a*]naphthalene-1,3(2*H*)-dione, named as gastrodinol D.

Compound **5** was isolated as yellow amorphous powder and it was assigned a molecular formula of C_25_H_22_O_6_ according to its positive HRESI-MS ion peak at *m/z* 417.1349 ([M–H] ^−^, calcd for 417.1344), which required 15 degrees of unsaturation. Its IR spectrum indicated the presence of hydroxyl (3371 cm^−1^), carbonyl (1712 cm^−1^), benzene (1635 cm^−1^) groups. The ^13^C NMR, DEPT and HSQC spectra of **5** (Table 2) suggested that the structure of the compound has 25 carbons, including one carbonyl, eighteen aromatic carbons, one oxymethine, one methylene, three methyl signals, one oxygen-bearing quaternary carbon. According to the chemical shift characteristics of C-2 (*δ*_C_ 75.94) oxymethine, C-3 (*δ*_C_ 43.24) methylene and C-4 (*δ*_C_ 197.85) carbonyl in ^13^C NMR spectrum, it was speculated that the parent nucleus of compound **5** was dihydroflavone. This was further confirmed by the HMBC (Fig. 2) correlations of H-8/C-4a and C-8a; H-2/C-3, C-4, C-4a, C-8a and C-1′; H-3′ and H-6′/C-1′. ^1^H NMR and ^13^C NMR data are similar to those of tetrapterol A [12] except the parent nucleus of dihydroflavones. Correlation of H-3′′ (*δ*_H_ 7.12)/H-4′′ (*δ*_H_ 7.03) in ^1^H-^1^H COSY spectra and HMBC spectra H-3′′/C-1′′, C-5′′ and C-8′′; H-4′′/C-2′′; H-7′′/C-5′′; H-9′′/C-2′′ and C-8′′ determined a 1,3,4-substituted phenyl derivatives as in tetrapterol A. HMBC spectra of H-3′/C-5′; H-6′/C-1′′; H-6′′/C-5′ supported that phenyl derivatives was linked to C-5′ with an α-dihydropyran ring as shown in tetrapterol A. In addition, through experimental analysis and ECD curve calculation, the results show that the experimental ECD spectra of compound **5** were basically consistent with the computed spectra of *R* (5), and the absolute configuration of compound **5** was determined as 2*R* (Fig. 3b). Accordingly, compound **5** was identified as 5,7-dihydroxy-2-(3-hydroxy-6,6,9-trimethyl-6*H*-benzo[*c*]chromen-2-yl) chroman-4-one, named as isoterapterol A.

**Table 2:** ^1^H NMR and ^13^C NMR spectroscopic date for compounds **5**–**8**

No.	5^a^		6^b^		7^b^		8^c^	
	*δ*_C_	*δ*_H_, m, *J* (Hz)	*δ*_C_	*δ*_H_, m, *J* (Hz)	*δ*_C_	*δ*_H_, m, *J* (Hz)	*δ*_C_	*δ*_H_, m, *J* (Hz)
2	75.94	5.67, dd, 13.0, 3.0	163.41	–	155.09	–	163.43	–
3	43.24	3.12, dd, 17.2, 13.0 2.77, dd, 17.2, 3.0	120.41	–	103.78	–	121.38	–
4	197.85	–	184.13	–	192.99	–	184.09	–
4a	102.97	–	105.49	–	100.64	–	105.52	–
5	165.55	–	162.66	–	163.91	–	157.98	–
6	96.79	5.88, d, 2.1	99.84	6.17, s	98.00	6.10, s	112.64	–
7	170.03	–	160.14	–	162.35	–	160.49	–
8	97.54	5.97, d, 2.1	101.63	–	102.00	–	107.39	–
8a	165.25	–	153.33	–	151.46	–	155.40	–
1′	120.69	–	112.74	–	114.56	–	113.59	–
2′	156.84	–	157.17	–	155.09	–	157.87	–
3′	105.26	6.39, s	104.27	6.54, d, 1.9	101.65	6.23, s	107.83	6.41, d, 2.3
4′	155.19	–	161.61	–	159.17	–	161.83	–
5′	115.89	–	108.22	6.51, dd, 8.6, 1.9	107.42	6.38, d, 8.2	103.73	6.39, dd, 8.3, 2.3
6′	122.48	7.86, s,	132.91	7.40, d, 8.6	123.98	7.41, d, 8.2	132.45	7.07, d, 8.3
1′′	129.71	–	115.32	6.60, d, 10.0	114.50	6.80, d, 10.1	22.73	3.39, d, 7.2
2′′	136.71	–	128.16	5.67, d, 10.0	128.10	5.81, d, 10.1	123.22	5.12, m
3′′	124.22	7.12, d, 7.9	78.88	–	78.75	–	132.57	–
4′′	128.71	7.02, d, 7.9	28.31	1.45, s	28.13	1.45, s	17.65	1.53, s
5′′	138.49	–	28.23	1.44, s	28.03	1.45, s	25.85	1.58, s
6′′	7.50, s	122.84						
7′′	2.34, s	21.32						
8′′	–	79.02						
9′′	1.56, s	27.99						
10′′	1.55, s	27.96						
1′′′			28.88	2.79, dd, 13.9, 2.6 2.48, dd, 13.9, 10.6	77.49	4.85, s	24.91	3.11, d, 7.0
2′′′			76.91	3.78, dd, 10.6, 2.6	86.43	3.83, s	123.04	5.12, m
3′′′			72.70	–	69.97	–	132.54	–
4′′′			25.19	1.09, s	25.88	1.05, s	17.80	1.40, s
5′′′			25.77	1.08, s	25.63	0.91, s	25.93	1.60, s
5-OH						11.87, s		
4′-OH						9.65, s		
2′′′-OOH						7.47, s		
3′′′-OH						4.55, s		
1′′′′							133.10	–
2′′′′, 6′′′′							130.42	7.09, d, 8.6
3′′′′, 5′′′′							115.80	6.64,d, 8.6
4′′′′							156.20	–
7′′′′							28.07	3.95, s

^a1^H NMR recorded at 400 MHz, ^13^C NMR recorded at 100 MHz in Methanol-*d*_4_

^b^^1^H NMR recorded at 600 MHz, ^13^C NMR recorded at 150 MHz in Acetone-*d*_6_

^c1^H NMR recorded at 500 MHz, ^13^C NMR recorded at 125 MHz in Methanol-*d*_4_

Compound **6** was purified as a yellow amorphous powder with molecular formula of C_25_H_26_O_8_ by the [M–H] ^–^ ion at *m/z* 453.1562 (calcd for 453.1555) in the HRESI-MS, exhibiting 13 degrees of unsaturation. The absorptions bands at 3421 and 1653 cm^−1^ in the IR spectrum explained the existence of hydroxyl, carbonyl groups. The ^13^C NMR, DEPT and HSQC spectra of **6** (Table 2) suggested that possessed 25 carbonds, including one carbonyl, sixteen aromatic carbons, one oxymethine, one methylene, four methyl signals, two oxygen-bearing quaternary carbons. Combined with ^1^H NMR and ^13^C NMR data, it was preliminarily speculated that compound **6** was an isopentenyl substituted flavonoids as morusinol [13]. This was further confirmed by the ^1^H-^1^H COSY correlations of H-1′′′/H-2′′′ and the HMBC (Fig. 2) correlations of H-2′′′/C-1′′′, C-3′′′, C-4′′′ and C-5′′′; H-4′′′ and H-5′′′/C-2′′′ and C-3′′′, and supported that hydroxyl was linked to C-2′′′. H-1′′′/C-2 and C-4; H-2′′′/C-3 supported that isopentenyl was linked to C-3. Combined with the molecular formula, the structure of compound **6** was determined. In summary, the structure of compound **6** was identified as 3-(2,3-dihydroxy-3-methylbutyl)-2-(2,4-dihydroxyphenyl)-5-hydroxy-8,8-dimethyl-4*H*,8*H*-pyrano[2,3-*f*] chromen-4-one, named as morusinol B.

Compound **7** was isolated as a yellow amorphous powder and had a chemical composition of C_25_H_4_O_9_ with 14 degrees of unsaturation from the HRESI-MS (*m/z*467.1353 [M–H] ^−^, calcd for 467.1348). The IR spectrum showed hydroxyl (3428 cm^−1^), carbonyl (1712 cm^−1^), phenyl (1631 cm^−1^) absorption signals of compound **7** strongly indicated the presence of a phenolic structure. By comparing the data of this compound with that of the morusin hydroperoxide [14], it was found that the structure of the two compounds was similar, except that compound **7** has a six-membered ring formed through an oxygen atom in C-1′′′ (*δ*_C_ 77.49) and C-2′ (*δ*_C_ 155.09). In addition, combined with the molecular formula and unsaturation of compound **7**, it was speculated that compound** 7** contained a peroxy hydroxyl. The HMBC (Fig. 2) correlated of 3′′′-OH/C-3′′′, C-4′′′ and C-2′′′ supported that hydroxyl was linked to C-3"′, and due to the chemical shift value of C-2′′′ (*δ*_C_ 86.43) moved to the low field to determine the position of peroxy hydroxyl (C-2′′′). According to the correlation between H-1′′′ (*δ*_H_ 4.85) and H-2′′′ (*δ*_H_ 3.83) in the ROESY spectrum, it was speculated that H-1′′′ and H-2′′′ were on the same side, so as to determine the relative configurations of compound **7** as 1R and 2R. Thus, the structure of **7** was determined as 8-(1-hydroperoxy-2-hydroxy-2-methylpropyl)-6,11-dihydroxy-3,3-dimethyl-3*H*,7*H*,8*H*-pyrano[3,2-*c*:6,5-*f*] dichromen-7-one, named as cyclomorusinol hydroperoxide.

Compound **8** was obtained as yellow amorphous powder. Its molecular formula as C_32_H_32_O_7_ was deduced from HRESI-MS (*m/z* 527.2071 [M–H] ^−^, calcd for 527.2075) with 17 degrees of unsaturation. The IR spectrum indicated the occurrence of a hydroxyl (3412 cm^−1^) and carbonyl (1620 cm^−1^, 1647 cm^−1^). The ^13^C NMR, DEPT and HSQC spectra of compound **8** (Table 2) showed 32 carbon resonances, including one carbonyl, twenty-four aromatic carbons, three oxygenated methylenes, four methyl signals. Combining the ^1^H NMR and ^13^C NMR data, it could be seen that it was very similar to that of kuwanon C (**12**) [7] except that compound **8** was linked to a *p*-hydroxyphenyl fragment in C-6. According to the single-peak methylene signal in ^1^H NMR spectrum [*δ*_*H*_ 3.95 (2H, s, H-7′′′′] and the carbon signals in ^13^C NMR spectrum with chemical shifts of 133.10 (C-1′′′′), 130.42(C-2′′′′, C-6′′′′), 115.80 (C-3′′′′, C-5′′′′), 156.20 (C-4′′′′), 28.07 (C-7′′′′), and combined with the type of compounds that had been resolved, confirmed that there was a *p*-hydroxybenzyl fragment in compound **8**. The HMBC correlations of (Fig. 2) H-2′′′′/C-7′′′′; H-7′′′′/C-1′′′′ and C-2′′′′; H-7′′′′/C-6 and C-7 was supported that hydroxyethyl was linked to C-6. The HMBC correlations of H-1′′/C-7, C-8 and C-8a supported that one isopentenyl was linked to C-8, H-1′′′/C-2 and C-4 supported that another isopentenyl was linked to C-3. Combined with the molecular formula, the structure of compound **8** was determined. In summary, the structure of compound **8** was identified as 2-(2,4-dihydroxyphenyl)-5,7-dihydroxy-6-(4-hydroxybenzyl)-3,8-bis(3-methylbut-2-en-1-yl)-4*H*-chromen-4-one, named as benzylkuwanon C.

Compound **9** was obtained as black amorphous powder. The molecular formula was assigned as C_41_H_32_O_9_ according to HRESI-MS (*m/z* 669.2126 [M + H] ^+^, calcd for 669.2119) with 26 degrees of unsaturation. The IR spectrum showed hydroxyl (3425 cm^−1^) phenyl (1622 cm^−1^). Comparing the ^1^H NMR and ^13^C NMR data of compound **9 ** (Table 3) with that of mulberrofuran G (**13**) [8], it could be speculated that both have the same parent nucleus. Considering the molecular formula of **9** and corresponding signals in NMR data, a *p*-substituted benzene could be inferred and which linked to C-2′ and the HMBC (Fig. 2) correlation of H-7′′′/C-2′ confirmed it further. The ROESY correlations of H-3′′/H-4′′; H-3′′/H-14′′ and H-4′′/H-14′′ supported that H-3′′′, H-4′′′ and ring B were on the same side, so the relative configurations of compound** 9** were determined as 3′′*R*, 4′′*R*, 5′′*S* and 8′′*R*. In summary, the structure of compound **9** was identified as 8a-(2,4-dihydroxyphenyl)-6-(6-hydroxybenzofuran-2-yl)-5-(4-hydroxybenzyl)-2-methyl-1,3a1,8a,13b-tetrahydro-3aH-benzo [3, 4] isochromeno[1,8-bc] chromene-4,11-diol, named as benzylmulberrofuran G.

**Table 3:** ^1^H NMR and ^13^C NMR spectroscopic date for compound **9** in acetone-*d*_6_

No.	*δ*_C_	*δ*_H_, m, *J* (Hz)	No.	*δ*_C_	*δ*_H_, m, *J* (Hz)
2	154.60	–	10′′	157.59	–
3	105.60	6.65, d, 0.9	11′′	104.62	6.42, d, 2.4
3a	122.38	–	12′′	159.93	–
4	121.99	7.33, d, 8.4	13′′	107.15	6.24, dd, 8.6, 2.4
5	113.09	6.77, dd, 8.4, 2.1	14′′	130.47	7.23, d, 8.6
6	156.64	–	15′′	117.56	–
7	98.28	6.94, dd, 2.1, 0.9	16′′	153.38	–
7a	156.45	–	17′′	103.95	6.36, d, 2.5
1′	131.49	–	18′′	157.78	–
2′	119.70	–	19′′	109.86	6.50, dd, 8.4, 2.5
3′	155.78	–	20′′	127.91	7.14, dd, 8.4, 1.2
4′	115.28	–	1′′′	131.98	–
5′	152.35	–	2′′′, 6′′′	129.83	6.90, d, 8.6
6′	109.56	7.03, s	3′′′, 5′′′	115.94	6.67, d, 8.6
1′′	134.39	–	4′′′	156.33	–
2′′	122.34	6.43, dd, 3.7, 2.4	7′′′	32.05	4.16, s
3′′	35.16	3.56, d, 5.6	6–OH	–	8.54, s
4′′	37.40	3.36, dd, 12.0, 5.4	3′–OH	–	7.49, s
5′′	28.52	2.99, m	10′′–OH	–	8.56, s
6′′	36.29	2.73, dd, 17.1, 5.4 2.02, dd, 17.1, 12.0	12′′–OH	–	8.50, s
7′′	23.96	1.77, s	18′′–OH	–	8.34, s
8′′	102.47	–	4′′′–OH	–	8.12, s
9′′	116.89	–			

^a1^H NMR recorded at 500 MHz, ^13^C NMR recorded at 125 MHz_。_

Compound **10** was obtained as yellow amorphous powder. The molecular formula was assigned as C_46_H_40_N_2_O_11_ according to HRESI-MS (*m/z* 797.2710 [M + H] ^+^, calcd for 797.2705) with 28 degrees of unsaturation. The IR absorption bands at 3417 cm^−1^, 1758 cm^−1^ resulted from the amino and carbonyl group. The ^1^H NMR spectrum data of compound **10** (Table 4) displayed that the compound had two 1,3,4-trisubstituted benzene ring fragments [*δ*_H_ 6.77 (1H, d, *J* = 2.0 Hz, H-11), 6.80 (1H, d, *J* = 8.0 Hz, H-14), 6.50 (1H, dd, *J* = 8.0, 2.0 Hz, H-15), 7.27 (1H, d, *J* = 2.0 Hz, H-2′′′), 6.75 (1H, d, *J* = 8.4 Hz, H-5′′′), 7.08 (1H, dd, *J* = 8.4, 2.0 Hz, H-6′′′)]; two 1,4-disubstituted benzene ring fragments [*δ*_**H**_ 7.02 (2H, d, *J* = 8.5 Hz, H-2′, H-6′), 6.70 (1H, d, *J* = 8.5 Hz, H-3′, H-5′), 6.82 (H-2′′, d, *J* = 8.5 Hz, 2H, H-6′′), 6.53 (2H, d, *J* = 8.5 Hz, H-3′′, H-5′′)]; four single peak aromatic hydrogens [*δ*_H_ 8.22 (1H, s, H-9), 7.80 (1H,s, H-8), 7.30 (1H, s, H-5), 7.18 (1H, s, H-7′′′)]; four methylenes [*δ*_H_ 3.74 (2H, m, H-8′), 2.81 (2H, m, H-7′), 3.41 (2H, td, *J* = 7.0, 5.7 Hz, H-8′′), 2.59 (2H, td, *J* = 7.0, 5.0 Hz, H-7′′)]; three methoxy groups [*δ*_H_ 4.12 (3H, s, 7-OCH_3_), 3.72 (3H, s, 12-OCH_3_) and 3.69 (3H, s, 3′′′-OCH_3_)]; an amino hydrogenl [*δ*_H_ 7.21 (1H, t, *J* = 5.9 Hz, 9′′′-CONH)]. The ^13^C NMR, DEPT and HSQC spectra of **10** (Table 4) suggested that **10** possessed 46 carbonds, including three carbonyls, 36 aromatic carbons (including eighteen aromatic quaternary carbons), four methylenes, three methoxys. The combination of ^1^H NMR and ^13^C NMR data of compound **10** with that of oleraisoindole A [15], more signals for a segment of *p*-hydroxyphenylethylamine and a segment of disubstituted styrene could be seen in **9**. The correlations of H-7′′/H-8′′; H-8′′/NH; H-2′′/H-3′′ in ^1^H-^1^H COSY spectrum, and H-7′′/C-1′′ and C-2′′; H-8′′/C-1′′; H-2′′/C-1′′ and C-7′′ in HMBC (Fig. 2) spectrum could speculated a fragment of *p*-hydroxyphenylethylamine further. The correlation of H-5′′′/H-6′′′ in ^1^H–^1^H COSY spectrum, and H-7′′′/C-1′′′, C-2′′′, C-6′′′ and C-8′′′ in HMBC spectrum confirmed a fragment of 3,4-Disubstituted styrene. The correlation of H-8′′, NH and H-7′′′/C-9′′′ was indicated that the stilbene and *p*-hydroxyphenylethylamine segments are linked by the carbonyl group at C-9′′′. The correlation of H-7′/H-8′; H-2′/H-3′ in ^1^H–^1^H COSY spectrum, and H-7′/C-1′ and C-2′; H-8′/C-1 and C-3; H-2′/C-7′ in HMBC spectrum were speculated to have a fragment of *N,N*-dicarbonyl *p*-hydroxyphenylethylamine. The correlation of H-5/C-4, C-6, C-7 and C-8a; H-8/C-4a, C-6, C-7 and C-9; H-9/C-3a, C-4a, C-8, C-8a and C-1, and the unsaturation of 28 were speculated to have a fragment of naphthalene ring and five-membered ring. The correlation of H-14/H-15 in ^1^H–^1^H COSY spectrum, the splitting of H-11/H-14/H-15 in ^1^H NMR, and H-11 and H-15/C-4 in HMBC spectrum shows that a 1,3,4-trisubstituted benzene ring fragment is connected with a naphthalene ring pentacyclic fragment by C_4_–C_10_ connection, and combined with the molecular formula of compound **10**, it was speculated that C-8′′′ and C-13 were connected by an oxygen atom, finally the structure of compound **10** was obtained. In summary, the structure of compound **10** was identified as (*Z*)-2-(4-(6-hydroxy-2-(4-hydroxyphenethyl)-7-methoxy-1,3-dioxo-2,3-dihydro-1*H*-benzo[1]isoindol-4-yl)-2-methoxyphenoxy)-3-(4-hydroxy-3-methoxyphenyl)-*N*-(4-hydroxyphenethyl) acrylamide, named as gastrodiamide.

**Table 4:** ^1^H NMR and ^13^C NMR spectroscopic date for compound **10** in acetone-*d*_6_

No.	*δ*_C_	*δ*_H_, m, *J* (Hz)	No.	*δ*_C_	*δ*_H_, m, *J* (Hz)
1	168.08	–	7′	34.10	2.81, m
3	167.51	–	8′	40.07	3.74, m
3a	123.37	–	1′′	130.58	–
4	139.46	–	2′′, 6′′	130.27	6.82, d, 8.5
4a	131.54	–	3′′, 5′′	115.91	6.53, d, 8.4
5	111.57	7.30, s	4′′	156.52	–
6	148.12	–	7′′	35.34	2.59, td, 7.0, 5.0
7	152.04	–	8′′	41.74	3.41, td, 7.0, 5.0
8	110.74	7.80, s	1′′′	125.14	–
8a	133.98	–	2′′′	113.43	7.27, d, 2.0
9	122.24	8.22, s	3′′′	148.18	–
9a	128.64	–	4′′′	148.82	–
10	126.06	–	5′′′	115.90	6.75, d, 8.4
11	114.79	6.77, d, 2.0	6′′′	125.37	7.08, dd, 8.4, 2.0
12	147.77	–	7′′′	123.89	7.18, s
13	147.58	–	8′′′	141.58	–
14	115.29	6.80, d, 8.0	9′′′	162.65	–
15	123.94	6.50, dd, 8.0, 2.0	7–OCH_3_	56.63	4.12, s
1′	130.05	–	12–OCH_3_	56.29	3.72, s
2′, 6′	130.55	7.02, d, 8.5	3′′′–OCH_3_	55.85	3.69, s
3′, 5′	116.04	6.70, d, 8.5	NH		7.21, t, 5.9
4′	156.82	–			

^a^^1^H NMR recorded at 500 MHz, ^13^C NMR recorded at 125 MHz

### The results of anti-MRSA activity of compounds

In order to screen the anti-MRSA active ingredient from the gastrodia stem, the anti-MRSA activity of 10 compounds (**1**, **2**, **3**, **5**, **6**, **7**, **9**, **11**, **12** and **13**) obtained by the isolation was preliminarily screened, and it was found that at a concentration of 100 μM, the inhibition rate of MRSA by 6 compounds (**1**, **5**, **6**, **11**, **12** and **13**) reached more than 80% (Table 5). Therefore, the three compounds were further screened for activity, and it was found that compound (**5, 11, 12** and **13**) had good anti-MRSA activity, and their MIC_50_ were 4.4 μM, 1.908 μM, 1.911 μM, 1.264 μM, respectively (Table 6).

**Table 5:** The results of anti-MRSA activity of compounds

Sample	Concentration (μM)	Inhibition rate (%)
Vancomycin	100	98.863 ± 0.495
1	100	98.688 ± 1.98
2	100	77.166 ± 1.609
3	100	13.474 ± 2.475
5	100	90.114 ± 0
6	100	88.627 ± 0.124
7	100	− 5.95 ± 2.475
9	100	63.955 ± 0.124
11	100	91.689 ± 0
12	100	95.364 ± 0.124
13	100	95.189 ± 0.99

**Table 6:** The MIC_50_ of anti-MRSA activity of compounds

Sample	MIC_50_ (μM)
Vancomycin	0.433 ± 0.002
1	22.057 ± 0.026
5	4.46 ± 0.02
6	61.639 ± 1.556
11	1.908 ± 0.014
12	1.911 ± 0.018
13	1.264 ± 0.005

### Acetylcholinesterase inhibition activity screening results

Acetylcholinesterase inhibition activity primary screening was performed on 10 compounds. The results showed that when the concentration was 50 μM, the inhibition activity of AChE was higher for 4 compounds (**2**, **3**, **9**, **13**) (inhibition rate greater than 60%). The four compounds were further screened for activity to obtain their IC_50_ values, which were 24.682 μM, 15.663 μM, 0.488 μM and 1.057 μM, respectively (Table 7).

**Table 7:** The IC_50_ of AChE-inhibiting activity of compounds

Sample	IC_50_ (μM)
TA	0.138 ± 0.005
2	24.682 ± 0.732
3	15.663 ± 1.565
9	0.488 ± 0.032
13	1.057 ± 0.011

## Experimental

### General

L-550 desktop low-speed centrifuge (Hunan Xiangyi Laboratory Instrument Development Co., Ltd.); AVANCE III 400 MHz, AVANCE III 500 MHz, AVANCE III 600 MHz and AVANCE III 800 MHz NMR spectrometers (Bruker GmbH)); V100 circular dichroic spectrometer (Applied Photophysics, UK); Autopol VI polarimeter (Rudolf, Germany); Shimadzu UV2401PC UV/VIS spectrophotometer (Shimadzu Corporation, Japan); Rotary evaporator (Heidolph AG of Germany); Agilent 1100 liquid chromatography (Agilent, USA), semi-prepared column ZORBAX SB-C18 (55 μm, 10 × 250 mm) (Agilent Corporation, USA); Thin layer chromatography silica gel, 200–300 mesh column chromatography silica gel (Qingdao Ocean Chemical Plant); Gel column chromatography Sephadex LH-20 (Pharmacia Corporation). The color developer is an ethanol solution of 10% H_2_SO_4_.

### Plant material

The stem, collected in 2019 from Zhaotong City, Yunnan Province, was identified by Professor Liu Shoujin of Anhui University of Chinese Medicine as derived from the *Gastrodia* *elata* Bl.

### Extraction and isolation

**Fig. 4:**
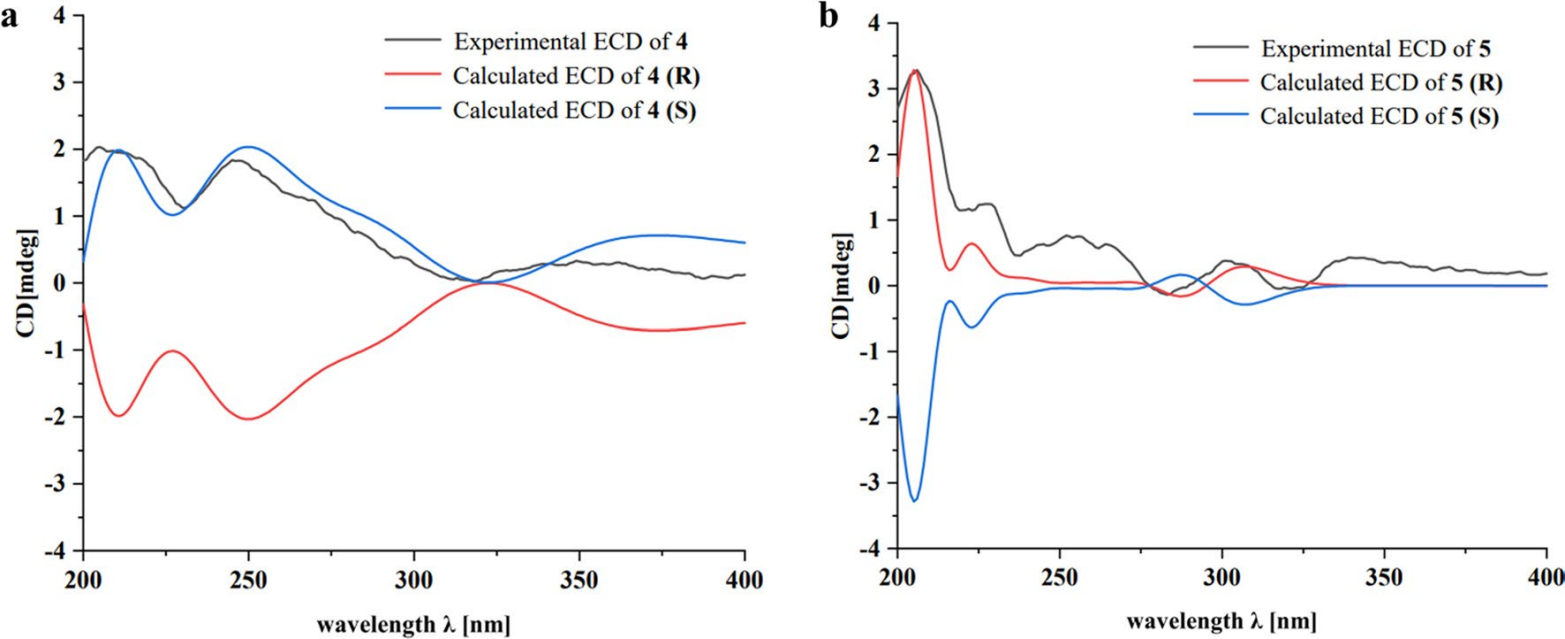
Flow chart of extraction and separation of the flow branch of *Gastrodia* *elata*

The dried flow branch (170 kg) of *G.* *elata* were re-extracted 3 times with 95% ethanol, each time for 2 h, the extraction solution was obtained, and then extracted 3 times with ethyl acetate and other volumes, and the ethyl acetate phase and aqueous phase were obtained after decompression and concentration. The ethyl acetate phase is detected by silica gel column chromatography (petroleum ether-acetone (elution of 100:1–0:1) gradient), TLC detection and merger of the same polarity part to obtain 8 components (Fr.1–Fr.8). Through TLC detection and color development results of color developers, select Fr.5. Fr.7 and Fr.8 LC–MS analysis was performed, and according to the analysis results, Fr.7 and Fr.8, were separated and purified. Drag Fr.7 (124 g) normal-phase silica gel column chromatographic section with petroleum ether/acetone as eluent and yielded 14 parts: Fr.7.1–Fr.7.14. Drag Fr. Part 7.10 was obtained by Sephadex LH-20 gel column chromatography (chloroform: methanol 1:1), and then by repeated atmospheric pressure normal-phase column chromatography (200–300 mesh), Sephadex LH-20 gel column chromatography (methanol) to obtain Compound **7** (yellow powder, 10 mg). Drag Fr.7.11 Part is obtained by Sephadex LH-20 gel column chromatography (chloroform: methanol 1:1), and then by repeated atmospheric pressure normal-phase column chromatography (200–300 mesh), Sephadex LH-20 gel column chromatography (methanol) to obtain compound **5** (yellow powder, 12 mg). Drag Fr.7.13 Part is obtained by Sephadex LH-20 gel column chromatography (chloroform: methanol 1:1), and then subjected to repeated atmospheric pressure normal-phase column chromatography (200–300 mesh) and recrystallization to obtain compound **11** (yellow amorphous powder, 50 mg). Drag Fr.8 (743 g) Atmospheric pressure normal-phase silicone column chromatographic section with chloroform/acetone as eluent yielded 13 parts: Fr.8.1–Fr.8.13. Drag Fr.8.6 (26 g) Atmospheric pressure normal-phase silicone column chromatographic section with chloroform/acetone as eluent yielded 6 parts: Fr.8.6.1–Fr.8.6.6. Fr.8.6.6 (5.3 g) partially purified by Sephadex LH-20 gel column chromatography (chloroform: methanol 1:1), repeated atmospheric pressure normal-phase column chromatography (200 to 300 mesh) and Sephadex LH-20 gel column chromatography, semi-prepared HPLC to obtain compounds** 1** (yellow powder, 5 mg), **2** (yellow powder, 23 mg),** 3** (yellow powder, 4 mg), **4** (yellow powder, 0.8 mg), **8** (yellow powder, 13 mg) and **12** (yellow powder, 22 mg). Drag Fr.8.8 Removed by MCI gives seven parts, Fr.8.8.1 to Fr.8.8.7. Fr.8.8.1 Compound** 9** (black powder, 29 mg) was obtained by repeated atmospheric pressure normal-phase column chromatography (200–300 mesh), Sephadex LH-20 gel column chromatography (chloroform: methanol 1:1) and Sephadex LH-20 gel column chromatography (methanol), and further semi-prepared HPLC purification to give compound **6** (yellow powder, 3 mg). Drag Fr.8.8.2 Compound **10** (light yellow powder, 7 mg) and Compound **13** (black amorphous powder, 53 mg) was purified by repeated atmospheric pressure normal-phase column chromatography (200–300 mesh), Sephadex LH-20 gel column chromatography (200 to 300 mesh), Sephadex LH-20 gel column chromatography (methanol), and semi-prepared HPLC (Fig. 4).

### Spectroscopic data of the isolates

#### Isogastrodinol (1)

Yellow powder; [*α*_L_^26^] − 2.81 (*c* 0.077, MeOH); UV (MeOH) λ_max_ (log *ε*): 195 (4.90), 221 (4.60), 279 (4.32), 377 (3.47) nm; IR (KBr) *v*_max_ 3413, 3021, 2924, 2852, 1631, 1614, 1594, 1151, 1542, 1446, 1422, 1394, 1331, 1236 cm^−1^; 1D NMR data see Table 1; HRESI-MS *m/z* 469.1662 [M–H]^−^, calcd for C_29_H_25_O_6_, 469.1657 (Additional file 1: Figs. 1–8).

#### Gastrodinol B (2)

Yellow powder; UV (MeOH) λ_max_ (log *ε*): 201 (4.81), 224 (4.88), 316 (4.68), 366 (3.74), 441 (3.53) nm; IR (KBr) *v*_max_ 3395, 3066, 3023, 2920, 2850, 1719, 1669, 1612, 1514, 1469, 1443, 1384, 1264 cm^−1^; 1D NMR data see Table 1; HRESI-MS *m/z* 673.1695 [M + CF_3_COO]^−^, calcd for C_35_H_28_O_7_CF_3_CO_2_, 673.1691 (Additional file 1: Figs. 9–16).

#### Gastrodinol C (3)

Yellow powder; UV (MeOH) λ_max_ (log *ε*): 201 (4.96), 226 (4.97), 277 (4.38), 319 (4.77) nm; IR (KBr) *v*_max_ 3432, 3027, 2971, 2920, 2851, 1713, 1668, 1613, 1514, 1484, 1442, 1417, 1383, 1359, 1261 cm^−1^; 1D NMR data see Table 1; HRESI-MS *m/z* 605.1812 [M + COOH]^−^, calcd for C_35_H_28_O_7_CO_2_H, 605.1817 (Additional file 1: Figs. 17–24).

#### Gastrodinol D (4)

Yellow powder; [*α*]_L_^26^ − 10.41 (*c* 0.078, MeOH); UV (MeOH) λ_max_ (log *ε*): 195 (4.71), 225 (4.23), 313 (3.90), 382 (3.21) nm; IR (KBr) *v*_max_ 3425, 3023, 2922, 2852, 1732, 1679, 1613, 1598, 1514, 1441, 1428, 1383, 1258 cm^−1^; 1D NMR data see Table 1; HRESI-MS *m/z* 673.1685 [M + CF_3_COO]^−^, calcd for C_35_H_28_O_7_CF_3_CO_2_, 673.1691 (Additional file 1: Figs. 25–32).

#### Isotetrapterols A (5)

Yellow powder; [*α*]_L_^20^ − 20.35 (*c* 0.115, MeOH); UV (MeOH) λ_max_ (log *ε*): 195 (4.55), 216 (4.66), 284 (4.34), 315 (416) nm; IR (KBr) *v*_max_ 3371, 3030, 2969, 2925, 2854, 1712, 1635, 1506, 1457, 1434, 1381, 1362, 1273 cm^−1^; 1D NMR data see Table 2; HRESI-MS *m/z* 417.1349 [M–H]^−^, calcd for C_25_H_21_O_6_, 417.1344 (Additional file 1: Figs. 33–40).

#### Morusinol B (6)

Yellow powder; [*α*]_L_^25^ − 23.00 (*c* 0.060, MeOH); UV (MeOH) λ_max_ (log *ε*): 199 (4.60), 225 (4.32), 271 (4.49), 348 (3.69) nm; IR (KBr) *v*_max_ 3421, 3263, 2975, 2930, 1653, 1622, 1602, 1579, 1483, 1434, 1382, 1351, 1255 cm^−1^; 1D NMR data see Table 2; HRESI-MS *m/z* 453.1562 [M–H]^−^, calcd for C_25_H_25_O_8_, 453.1555 (Additional file 1: Figs. 41–48).

#### Cyclomorusinol hydroperoxide (7)

Yellow powder; [*α*]_L_^20^ − 18.10 (*c* 0.084, MeOH); UV (MeOH) λ_max_ (log ε): 203 (4.31), 227 (3.82), 273 (4.02), 311 (3.58), 367 (3.14) nm; IR (KBr) *v*_max_ 3428, 3074, 2974, 2928, 2856, 1712, 1631, 1533, 1508, 1461, 1402, 1383, 1278 cm^−1^; 1D NMR data see Table 2; HRESI-MS *m/z* 467.1353 [M − H]^−^, calcd for C_25_H_23_O_9_, 467.1348 (Additional file 1: Figs. 49–56).

#### Benzylkuwanon C (8)

Yellow powder; UV (MeOH) λ_max_ (log *ε*): 203 (5.05), 268 (4.65), 305 (4.29) nm; IR (KBr) *v*_max_ 3412, 3041, 2969, 2925, 2856, 1647, 1620, 1562, 1512, 1466, 1441, 1357, 1222 cm^−1^; 1D NMR data see Table 2; HRESI-MS *m/z* 527.2071 [M–H]^−^, calcd for C_32_H_31_O_7_, 527.2075 (Additional file 1: Figs. 57–64).

#### Benzylmulberrofuran G (9)

Black powder; [*α*]_D_^20^  + 475.49 (*c* 0.142 MeOH); UV (MeOH) λ_max_ (log *ε*): 198 (5.03), 223 (4.61), 284 (4.20), 314 (4.28) nm; IR (KBr) *v*_max_ 3425, 3069, 2924, 2853, 1712, 1622, 1563, 1511, 1488,1449,1414, 1364, 1257 cm^−1^; 1D NMR data see Table 3. HRESI-MS *m/z* 669.2126 [M + H]^+^, calcd for C_41_H_33_O_9_, 669.2119 (Additional file 1: Figs. 65–72).

#### gastrodiamide (10)

Light yellow powder; IR (KBr) *v*_max_ 3417, 3263, 3069, 3016, 2930, 2853, 1758, 1702, 1630, 1613, 1514, 1470, 1434, 1404, 1383, 1255 cm^−1^; 1D NMR data see Table 4. HRESI-MS *m/z* 797.2710 [M + H]^+^, calcd for C_46_H_41_N_2_O_11_, 797.2705 (Additional file 1: Figs. 73–79).

#### Cyclomulberrin (11)

Yellow amorphous powder; C_25_H_24_O_6_; ^1^H NMR (600 MHz, Methanol-*d*_4_) *δ*_H_: 7.61 (d, *J* = 8.6 Hz, 1H, H-6′), 6.52 (dd, *J* = 8.6, 2.2 Hz, 1H, H-5′), 6.32 (d, *J* = 2.2 Hz, 1H, H-3′), 6.22 (s, 1H, H-6), 6.15 (d, *J* = 9.2 Hz, 1H, H-1′′′), 5.42 (d, *J* = 9.2 Hz, 1H, H-2′′′), 5.26 (t, *J* = 5.9 Hz, 1H, H-2′′), 3.58 (dd, *J* = 5.9, 15 Hz, 2H, H-1′′), 1.95 (s, 3H, H-4′′′), 1.84 (s, 3H, H-5′′′), 1.70 (s, 3H, H-5′′), 1.69 (s, 3H, H-4′′); ^13^C NMR (150 MHz, Methanol-*d*_4_) *δ*_C_: 179.93 (C-4), 164.70 (C-8a), 162.87 (C-7), 160.94 (C-4′), 159.58 (C-2), 157.16 (C-2′), 155.72 (C-5), 139.79 (C-3′′′), 132.41 (C-3′′), 126.21 (C-6′), 123.79 (C-2′′), 122.56 (C-2′′′), 110.93 (C-5′), 110.04 (C-1′), 108.87 (C-3), 108.15 (C-4a), 105.59 (C-8), 105.05 (C-3′), 99.40 (C-6), 70.71 (C-1′′′), 25.90 (C-4′′, 5′′), 22.49 (C-1′′), 18.68 (C-4′′′), 18.17 (C-5′′′).

#### Kuwanon C (12)

Yellow amorphous powder; C_25_H_26_O_6_; ^1^H NMR (500 MHz, Methanol-*d*_4_) *δ*_H_: 7.07 (d, *J* = 8.3 Hz, 1H, H-6′), 6.42 (d, *J* = 2.3 Hz, 1H, H-3′), 6.39 (dd, *J* = 8.3, 2.3 Hz, 1H, H-5′), 6.23 (s, 1H, H-6), 5.16 (m, 1H, H-2′′), 5.10 (m, 1H, H-2′′′), 3.32 (d, *J* = 7.1 Hz, 2H, H-1′′), 3.09 (d, *J* = 6.9 Hz, 2H, H-1′′′), 1.59 (s, 6H, H-4′′′, H-5′′′), 1.55 (s, 3H, H-5′′), 1.39 (s, 3H, H-4′′); ^13^C NMR (125 MHz, Methanol-*d*_4_) *δ*_C_: 184.02 (C-4), 163.59 (C-7), 162.71 (C-4′), 161.82 (C-8a), 160.68 (C-2), 157.86 (C-2′), 157.07 (C-5), 132.60 (C-3′′′), 132.42 (C-6′), 132.01 (C-3′′), 123.43 (C-2′′), 122.97 (C-2′′′), 121.33 (C-3), 113.53 (C-1′), 107.84 (C-5′), 107.50 (C-8), 105.34 (C-4a), 103.73 (C-3′), 98.92 (C-6), 25.94 (C-4′′′), 25.84 (C-4′′), 24.83 (C-1′′′), 22.34 (C-1′′), 17.75 (C-5′′′), 17.65 (C-5′′).

#### Mulberrofuran G (13)

Black amorphous powder; C_34_H_26_O_8_; ^1^H NMR (400 MHz, Methanol-*d*_4_) *δ*_H_: 7.34 (d, *J* = 8.4 Hz, 1H, H-4), 7.14 (d, *J* = 8.4 Hz, 1H, H-14′′), 7.09 (d, *J* = 8.4 Hz, 1H, H-20′′), 6.92 (s, 1H, H-7), 6.91 (d, *J* = 1.8 Hz, 2H, H-2′, H-6′), 6.82 (s, 1H, H-3), 6.73 (dd, *J* = 8.4, 2.1 Hz, 1H, H-5), 6.46 (dd, *J* = 8.4, 2.6 Hz, 1H, H-19′′), 6.40 (d, *J* = 4.7 Hz, 1H, H-2′′), 6.35 (d, *J* = 2.6 Hz, 1H, H-17′′), 6.33 (d, *J* = 2.4 Hz, 1H, H-11′′), 6.14 (dd, *J* = 8.6, 2.4 Hz, 1H, H-13′′), 3.34 – 3.23 (m, 2H, H-3′′, H-4′′), 2.95 (dd, *J* = 11.4, 5.5 Hz, 1H, H-5′′), 2.66 (dd, *J* = 17.0, 5.5 Hz, 1H, H-6′′a), 2.01 (dd, *J* = 17.0, 11.4 Hz, 1H, H-6′′b), 1.78 (s, 3H, H-7′′); ^13^C NMR (100 MHz, Methanol-*d*_4_) *δ*_C_: 158.65 (C-10′′), 157.02 (C-18′′), 156.51 (C-12′′), 156.33 (C-6), 155.85 (C-2), 155.39 (C-5′), 154.34 (C-3′), 153.52 (C-16′′), 152.21 (C-7a), 132.52 (C-1′′), 130.14 (C-1′), 129.18 (C-14′′), 126.60 (C-20′′), 121.96 (C-2′′), 121.71 (C-3a), 120.60 (C-4), 116.89 (C-4′), 115.96 (C-15′′), 112.52 (C-9′′), 111.85 (C-5), 108.65 (C-19′′), 105.63 (C-13′′), 104.10 (C-3), 103.60 (C-6′), 103.15 (C-2′), 102.78 (C-17′′), 101.70 (C-8′′), 100.76 (C-11′′), 97.10 (C-7), 36.24 (C-3′′), 35.34 (C-6′′), 34.02 (C-5′′), 27.44 (C-4′′), 22.54 (C-7′′).

### Compound anti-Staphylococcus aureus activity experiment

Prepare samples with DMSO as a solvent into a solution with an initial concentration of 20 mM; Take a 96-well culture plate, dilute the sample to be measured, and add bacterial solution to each well, and the final concentration is 5 × 10^5^ CFU/mL; Incubate at 37 °C for 24 h, and the microplate reader determines the OD value at 625 nm. The experiment also set up a media blank control, a bacterial control and a vancomycin-positive drug control.

MIC_50_ (50% minimum inhibitory concentration) is calculated according to the Reed & Muench method.

### Acetylcholinesterase inhibition activity screening results

Use phosphate buffer (0.1 M Na_2_HPO_4_ solution per 100 mL of phosphate buffer 94.7 mL; 0.1 M NaH_2_PO_4_ solution 5.3 mL, pH adjusted to 8.0) Dilute AChE into 0.1 U/mL working solution; Thioacetylcholine iodide and DTNB are formulated with phosphate buffer to form a 6.25 mM solution (working solution); The compound is diluted with DMSO to form a concentration gradient. The positive control is tacrine, diluted with DMSO to a concentration gradient; The negative control group (NC group) was a 2% DMSO solvent control. The reaction is carried out in a 96-well plate with plates plated by 200 μL/system with 3 replicates per sample; Detect the absorbance value of 405 nm every 30 s within 1 h after the addition of the color developer and substrate. Select the sample absorbance value when the average absorbance value of the NC group is about 1, calculate the average value of the compound absorbance value (compound measurement value—background value), and calculate the compound AChE inhibition rate according to (NC-compound absorbance value average)/NC*100%.

## Conclusions

The traditional Chinese medicine “Tianma” from *Gastrodia* *elata* has a long history and has a regulatory effect on the nervous system. Previous studies mainly focused on the tubers and have shown that *G.* *elata* mainly contains gastrodin and other natural products with benzyl alcohol as the basic unit. Pharmaceutical research herein on the flower branch of *G.* *elata* and a completely different chemical composition was founded to that of the tubers of *G.* *elata*. Excepte gastrodinols **1**–**4** that enriched chemical diversity of this kind of composition, isopentenyl-substituted flavonoids (**5**–**8** and **11**–**12**), mulberrofurans (**9**, **13**) and gastrodiamide (**10**) were isolated for the first time from the species *Gastrodia*. Isopentenyl-substituted flavonoids and mulberry furans are mostly reported from mulberry bark [16], and it’s extremely rare for the isolation of these compounds from Orchidaceae. With the foundation and confirmation of isopentenyltransferase by heterologous expression in *Oberonia* *myosurus* [17], it could be indicated that pentenyl-substitution is a universality in Orchidaceae.

The isolated new compound part was screened for activity, and it was found that compound **5**, **11**, **12** and **13** had good anti-MRSA activity, and their MIC_50_ were 4.4 μM, 1.908 μM, 1.911 μM, 1.264 μM, respectively. compound **2**, **3**,** 9** and **13** had a higher inhibitory activity for AChE, and their IC_50_ were 24.682 μM, 15.663 μM, 0.488 μM and 1.057 μM, respectively. The results showed that compound** 2**, **3**, **5**, **9**, **11**, **12**, **13** are expected to develop called antibacterial drugs or neurodrugs. In addition, the study of the flow branch of *G*. *elata* is also necessary and should attract people's attention.

### Supplementary Information

**Additional file 1: Figure S1. **HRESIMS of compound **1**. **Figure S2. **Ultraviolet spectra of compound **1**. **Figure S3. **Infrared spectra of compound **1**. **Figure S4. **^1^H-NMR of compound **1**. **Figure S5. **^13^C-NMR of compound **1**. **Figure S6. **HSQC of compound **1**. **Figure S7. **^1^H-^1^H COSY of compound **1**. **Figure S8. **HMBC of compound **1**. **Figure S9. **HRESIMS of compound **2**. **Figure S10. **Ultraviolet spectra of compound **2. Figure S11. **Infrared spectra of compound **2. Figure S12. **^1^H-NMR of compound **2**. **Figure S13. **^13^C-NMR of compound **2**. **Figure S14. **HSQC of compound **2**. **Figure S15. **^1^H-^1^H COSY of compound **2**. **Figure S16. **HMBC of compound **2**. **Figure S17. **HRESIMS of compound **3**. **Figure S18. **Ultraviolet spectra of compound **3**. **Figure S19. **Infrared spectra of compound **3**. **Figure S20. **^1^H-NMR of compound **3**. **Figure S21. **^13^C-NMR of compound **3**. **Figure S22. **HSQC of compound **3**. **Figure S23. **^1^H-^1^H COSY of compound **3**. **Figure S24. **HMBC of compound **3**. **Figure S25. **HRESIMS of compound **4**. **Figure S26. **Ultraviolet spectra of compound **4. Figure S27. **Infrared spectra of compound **4**. **Figure S28. **^1^H-NMR of compound **4**. **Figure S29. **^13^C-NMR of compound **4**. **Figure S30. **HSQC of compound **4**. **Figure S31. **^1^H-^1^H COSY of compound **4**. **Figure S32. **HMBC of compound **4**. **Figure S33. **HRESIMS of compound **5**. **Figure S34. **Ultraviolet spectra of compound **5. Figure S35. **Infrared spectra of compound **5. Figure S36. **^1^H-NMR of compound **5**. **Figure S37. **^13^C-NMR of compound **5**. **Figure S38. **HSQC of compound **5**. **Figure S39. **^1^H-^1^H COSY of compound **5**. **Figure S40. **HMBC of compound **5**. **Figure S41. **HRESIMS of compound **6**. **Figure S42. **Ultraviolet spectra of compound **6. Figure S43. **Infrared spectra of compound **6. Figure S44. **^1^H-NMR of compound **6**. **Figure S45. **^13^C-NMR of compound **6**. **Figure S46. **HSQC of compound **6. Figure S47. **^1^H-^1^H COSY of compound **6**. **Figure S48. **HMBC of compound **6**. **Figure S49. **HRESIMS of compound **7**. **Figure S50. **Ultraviolet spectra of compound **7. Figure S51. **Infrared spectra of compound **7. Figure S52. **^1^H-NMR of compound **7**. **Figure S53. **^13^C-NMR of compound **7**. **Figure S54. **HSQC of compound **7. Figure S55. **^1^H-^1^H COSY of compound **7**. **Figure S56. **HMBC of compound **7**. **Figure S57. **HRESIMS of compound **8**. **Figure S58. **Ultraviolet spectra of compound **8. Figure S59. **Infrared spectra of compound **8. Figure S60. **^1^H-NMR of compound **8**. **Figure S61. **^13^C-NMR of compound **8**. **Figure S62. **HSQC of compound **8. Figure S63. **^1^H-^1^H COSY of compound **8**. **Figure S64. **HMBC of compound **8**. **Figure S65. **HRESIMS of compound **9**. **Figure S66. **Ultraviolet spectra of compound **9. Figure S67. **Infrared spectra of compound **9. Figure S68. **^1^H-NMR of compound **9**. **Figure S69. **^13^C-NMR of compound **9**. **Figure S70. **HSQC of compound **9. Figure S71. **^1^H-^1^H COSY of compound **9**. **Figure S72. **HMBC of compound **9**.** Figure S73. **HRESIMS of compound **10**. Ultraviolet spectra of compound. **Figure S74. **Infrared spectra of compound **10. Figure S75. **^1^H-NMR of compound **10**. **Figure S76. **^13^C-NMR of compound **10**. **Figure S77. **HSQC of compound **10. Figure S78. **^1^H-^1^H COSY of compound **10**. **Figure S79. **HMBC of compound **10**.

## Data Availability

The data supporting the results of this study can be obtained from the corresponding authors upon reasonable request.
