# Supplementary material for: Gastrodinol derivatives and prenylated flavones from the flower branch of Gastrodia elata
Source: Nat Prod Bioprospect. 2024 Mar 20;14(1):22. doi: 10.1007/s13659-024-00430-6 (PMC10954593; doi:10.1007/s13659-024-00430-6)
Supplement: Supplementary file 1 — Additional file 1: Figure S1. HRESIMS of compound 1. Figure S2. Ultraviolet spectra of compound 1. Figure S3. Infrared spectra of compound 1. Figure S4. 1H-NMR of compound 1. Figure S5. 13C-NMR of compound 1. Figure S6. HSQC of compound 1. Figure S7. 1H-1H COSY of compound 1. Figure S8. HMBC of compound 1. Figure S9. HRESIMS of compound 2. Figure S10. Ultraviolet spectra of compound 2. Figure S11. Infrared spectra of compound 2. Figure S12. 1H-NMR of compound 2. Figure S13. 13C-NMR of compound 2. Figure S14. HSQC of compound 2. Figure S15. 1H-1H COSY of compound 2. Figure S16. HMBC of compound 2. Figure S17. HRESIMS of compound 3. Figure S18. Ultraviolet spectra of compound 3. Figure S19. Infrared spectra of compound 3. Figure S20. 1H-NMR of compound 3. Figure S21. 13C-NMR of compound 3. Figure S22. HSQC of compound 3. Figure S23. 1H-1H COSY of compound 3. Figure S24. HMBC of compound 3. Figure S25. HRESIMS of compound 4. Figure S26. Ultraviolet spectra of compound 4. Figure S27. Infrared spectra of compound 4. Figure S28. 1H-NMR of compound 4. Figure S29. 13C-NMR of compound 4. Figure S30. HSQC of compound 4. Figure S31. 1H-1H COSY of compound 4. Figure S32. HMBC of compound 4. Figure S33. HRESIMS of compound 5. Figure S34. Ultraviolet spectra of compound 5. Figure S35. Infrared spectra of compound 5. Figure S36. 1H-NMR of compound 5. Figure S37. 13C-NMR of compound 5. Figure S38. HSQC of compound 5. Figure S39. 1H-1H COSY of compound 5. Figure S40. HMBC of compound 5. Figure S41. HRESIMS of compound 6. Figure S42. Ultraviolet spectra of compound 6. Figure S43. Infrared spectra of compound 6. Figure S44. 1H-NMR of compound 6. Figure S45. 13C-NMR of compound 6. Figure S46. HSQC of compound 6. Figure S47. 1H-1H COSY of compound 6. Figure S48. HMBC of compound 6. Figure S49. HRESIMS of compound 7. Figure S50. Ultraviolet spectra of compound 7. Figure S51. Infrared spectra of compound 7. Figure S52. 1H-NMR of compound 7. Figure S53. 13C-NMR of compound 7. Figure S54. H [file 13659_2024_430_MOESM1_ESM.pdf]

Supporting information

Gastrodinol derivatives and prenylated flavones from the flower branch of *Gastrodia elata*

Shi-Hui Qin[1,2+], Zhi-Lan Li[1+], Liu Yang[1], Jiang-Miao Hu[1*]

[1]State Key Laboratory of Phytochemistry and Plant Resources in West China, Kunming Institute of Botany, Chinese Academy of Sciences, Kunming 650201, Yunnan, China

[2]College of Pharmacy, Anhui University of Chinese Medicine, Hefei 230012, China

* Corresponding author. E-mail address: hujiangmiao@mail.kib.ac.cn (J.M. Hu)

[+] These two authors contributed equally to this work.

Supporting Information List

Page 4: HRESIMS data of compound **1**

Page 5: Ultraviolet and Infrared spectra of compound **1**

Page 6: ^{1}H NMR and ^{13}C NMR (DEPT) spectrum of compound **1**

Page 7: HSQC and ^{1}H-^{1}H COSY spectrum of compound **1**

Page 8: HMBC spectrum of compound **1**

Page 9: HRESIMS data of compound **2**

Page 10: Ultraviolet and Infrared spectra of compound **2**

Page 11: ^{1}H NMR and ^{13}C NMR (DEPT) spectrum of compound **2**

Page 12: HSQC and ^{1}H-^{1}H COSY spectrum of compound **2**

Page 13: HMBC spectrum of compound **2**

Page 14: HRESIMS data of compound **3**

Page 15: Ultraviolet and Infrared spectra of compound **3**

Page 16: ^{1}H NMR and ^{13}C NMR (DEPT) spectrum of compound **3**

Page 17: HSQC and ^{1}H-^{1}H COSY spectrum of compound **3**

Page 18: HMBC spectrum of compound **3**

Page 19: HRESIMS data of compound **4**

Page 20: Ultraviolet and Infrared spectra of compound **4**

Page 21: ^{1}H NMR and ^{13}C NMR (DEPT) spectrum of compound **4**

Page 22: HSQC and ^{1}H-^{1}H COSY spectrum of compound **4**

Page 23: HMBC spectrum of compound **4**

Page 24: HRESIMS data of compound **5**

Page 25: Ultraviolet and Infrared spectra of compound **5**

Page 26: ^{1}H NMR and ^{13}C NMR (DEPT) spectrum of compound **5**

Page 27: HSQC and ^{1}H-^{1}H COSY spectrum of compound **5**

Page 28: HMBC spectrum of compound **5**

Page 29: HRESIMS data of compound **6**

Page 30: Ultraviolet and Infrared spectra of compound **6**

Page 31: ^{1}H NMR and ^{13}C NMR (DEPT) spectrum of compound **6**

Page 32: HSQC and ^{1}H-^{1}H COSY spectrum of compound **6**

Page 33: HMBC spectrum of compound **6**

Page 34: HRESIMS data of compound **7**

Page 35: Ultraviolet and Infrared spectra of compound **7**

Page 36: ^{1}H NMR and ^{13}C NMR (DEPT) spectrum of compound **7**

Page 37: HSQC and ^{1}H-^{1}H COSY spectrum of compound **7**

Page 38: HMBC spectrum of compound **7**

Page 39: HRESIMS data of compound **8**

Page 40: Ultraviolet and Infrared spectra of compound **8**

Page 41: ^{1}H NMR and ^{13}C NMR (DEPT) spectrum of compound **8**

Page 42: HSQC and ^{1}H-^{1}H COSY spectrum of compound **8**

Page 43: HMBC spectrum of compound **8**

Page 44: HRESIMS data of compound **9**

Page 45: Ultraviolet and Infrared spectra of compound **9**

Page 46: ^{1}H NMR and ^{13}C NMR (DEPT) spectrum of compound **9**

Page 47: HSQC and ^{1}H-^{1}H COSY spectrum of compound **9**

Page 48: HMBC spectrum of compound **9**

Page 49: HRESIMS data of compound **10**

Page 50: Infrared spectra of compound **10**

Page 51: ^{1}H NMR and ^{13}C NMR (DEPT) spectrum of compound **10**

Page 52: HSQC and ^{1}H-^{1}H COSY spectrum of compound **10**

Page 53: HMBC spectrum of compound **10**

Qualitative Analysis Report

**Data Filename**	ZLZL-155.d	**Sample Name**	ZLZL-155
**Sample Type**	Sample	**Position**	P1-A5
**Instrument Name**	Instrument 1	**User Name**	
**Acq Method**	s-.m	**Acquired Time**	1/19/2022 10:58:30 AM
**IRM Calibration Status**	Success	**DA Method**	PCDL.m
**Comment**			

Sample Group **Info.**
Acquisition SW Version 6200 series TOF/6500 series Q-TOF B.05.01 (B5125.2)

User Spectra

Fragmentor Voltage	Collision Energy	Ionization Mode
135	0	ESI

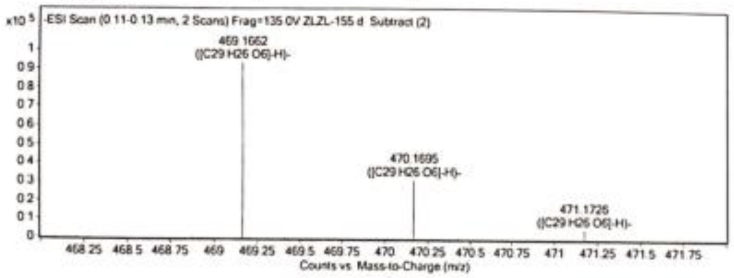

Peak List

m/z	z	Abund	Formula	Ion
226.9788	1	33401.4		
285.0328	1	25380.17		
312.1247	1	58198.37		
313.1287	1	12086.04		
322.1095	1	23606.51		
426.1178	1	11429.35		
457.0778	1	12446.16		
469.1662	1	93725.33	C29 H26 O6	(M-H)-
470.1695	1	31818.86	C29 H26 O6	(M-H)-
505.1426	1	13546.31		

Formula Calculator Element Limits

Element	Min	Max
C	3	60
H	0	120
O	0	30
N	0	10

Formula Calculator Results

Formula	CalculatedMass	CalculatedMz	Mz	Diff. (mDa)	Diff. (ppm)	DBE
C29 H26 O6	470.1729	469.1657	469.1662	-0.50	-1.07	17.0000

--- End Of Report ---

Figure S1. HRESIMS of compound **1**.

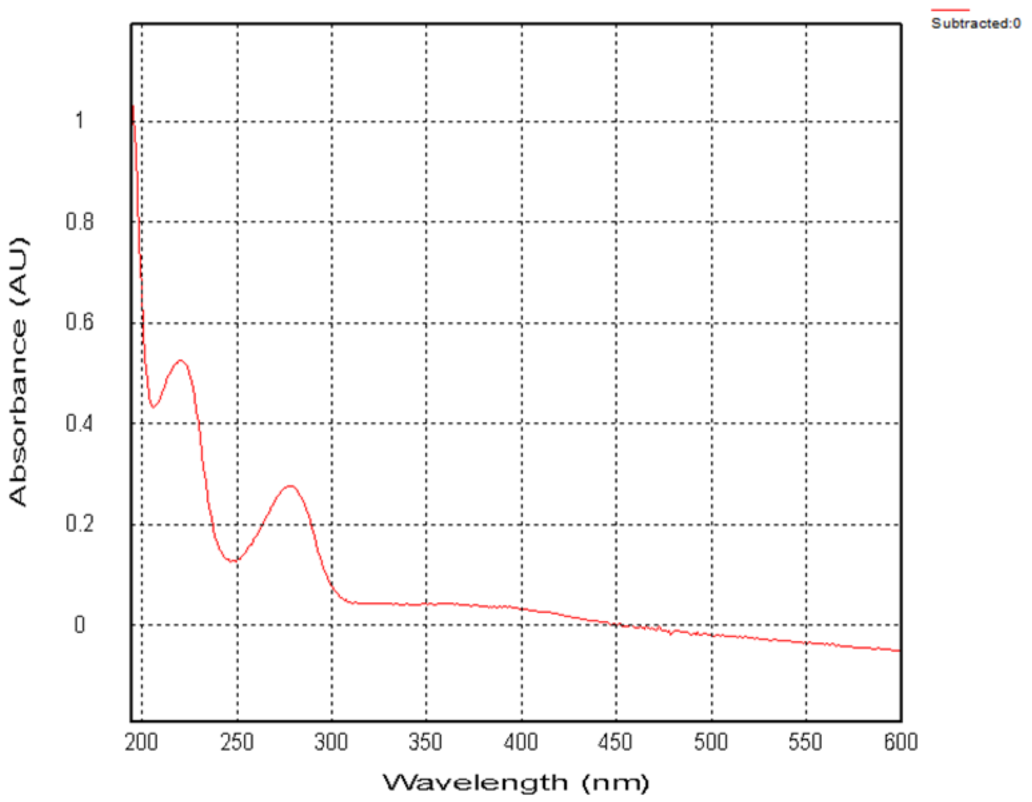

Figure S2. Ultraviolet spectra of compound **1**.

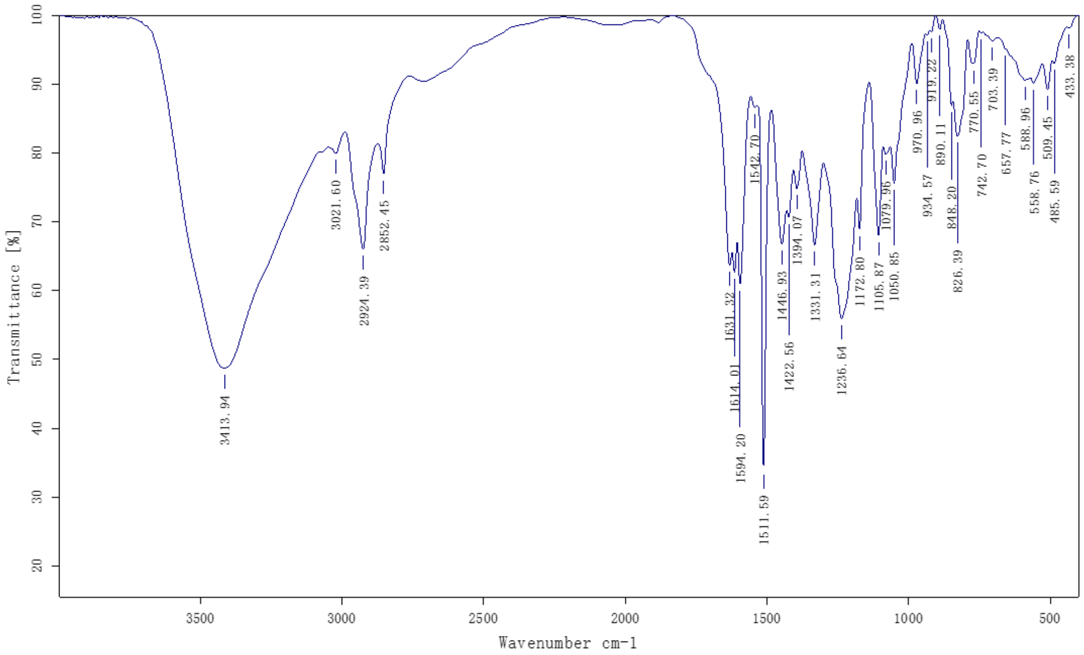

Figure S3. Infrared spectra of compound **1**.

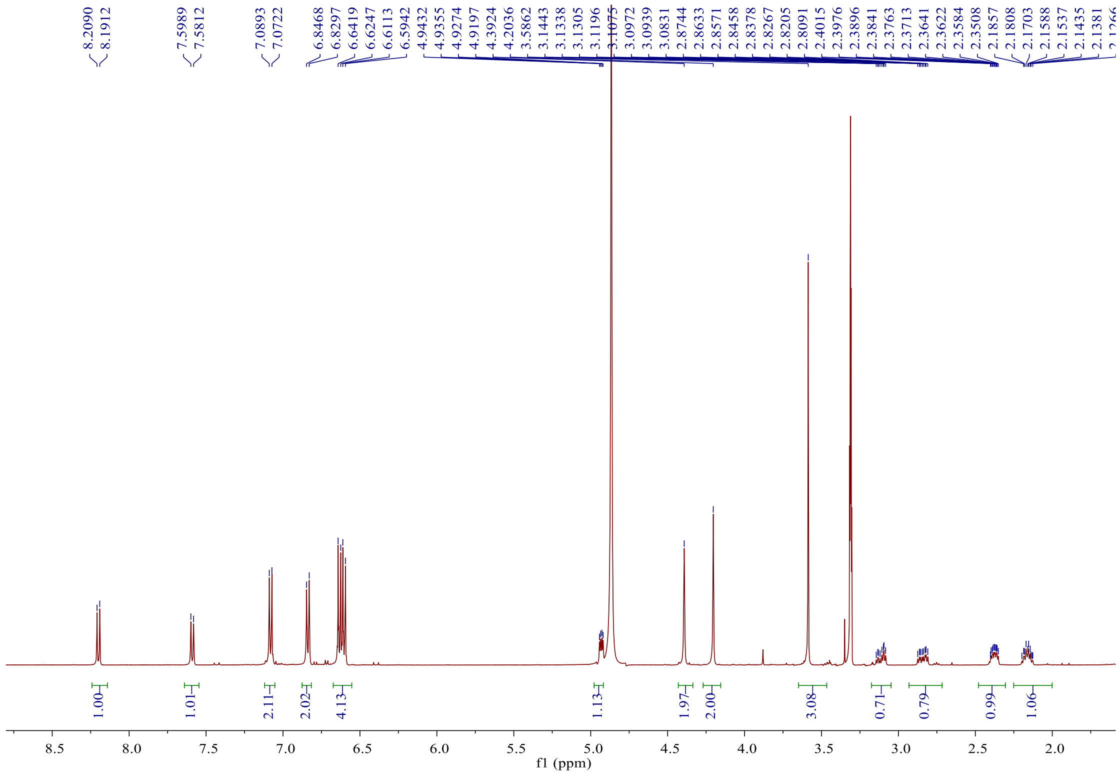

Figure S4. ^{1}H-NMR of compound **1**.

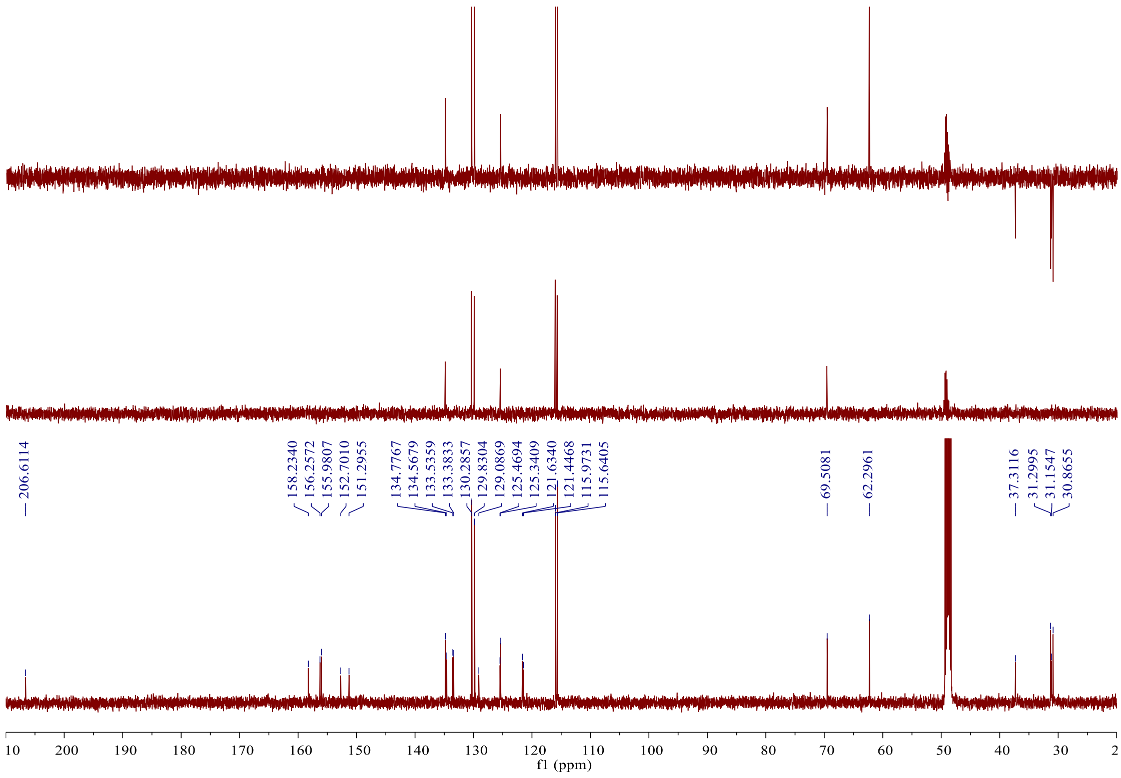

Figure S5. ^{13}C-NMR of compound **1**.

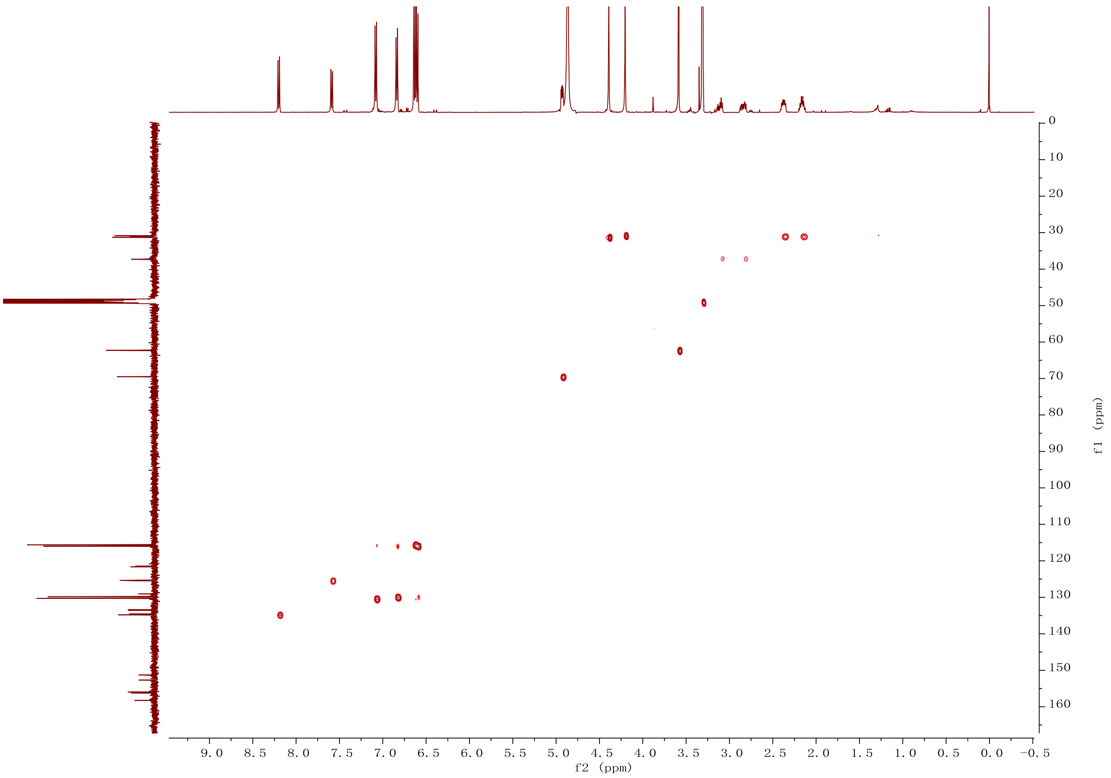

Figure S6. HSQC of compound **1**.

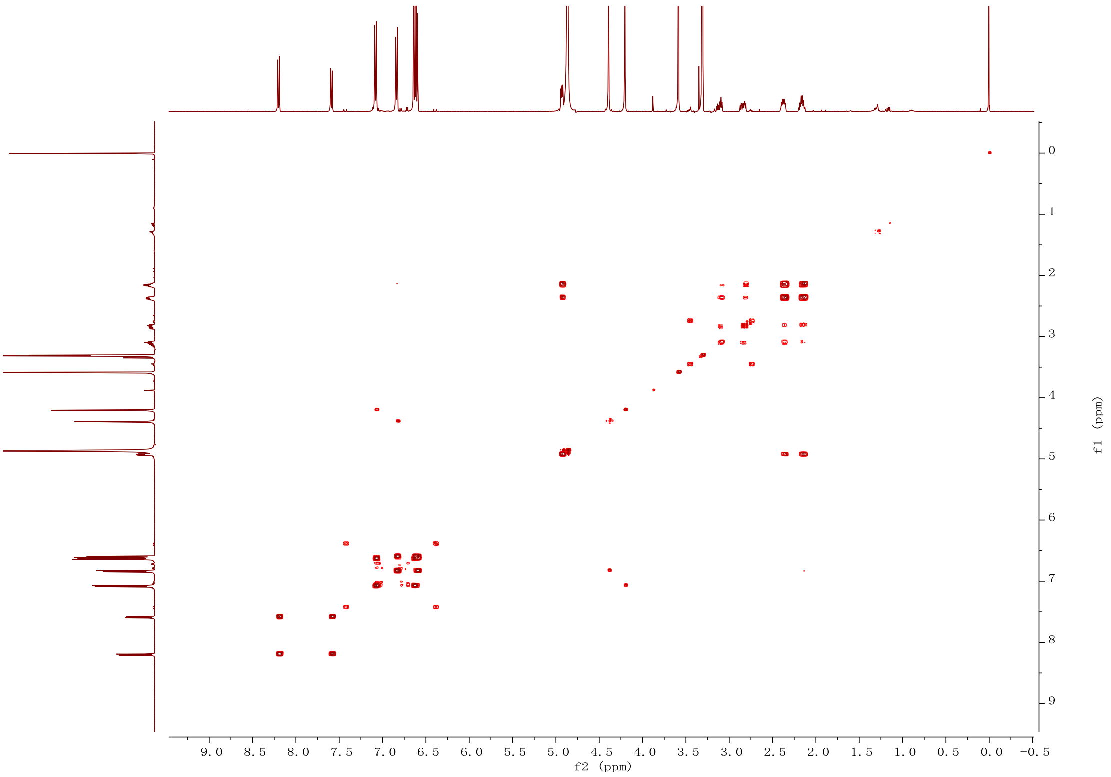

Figure S7. ^{1}H-^{1}H COSY of compound **1**.

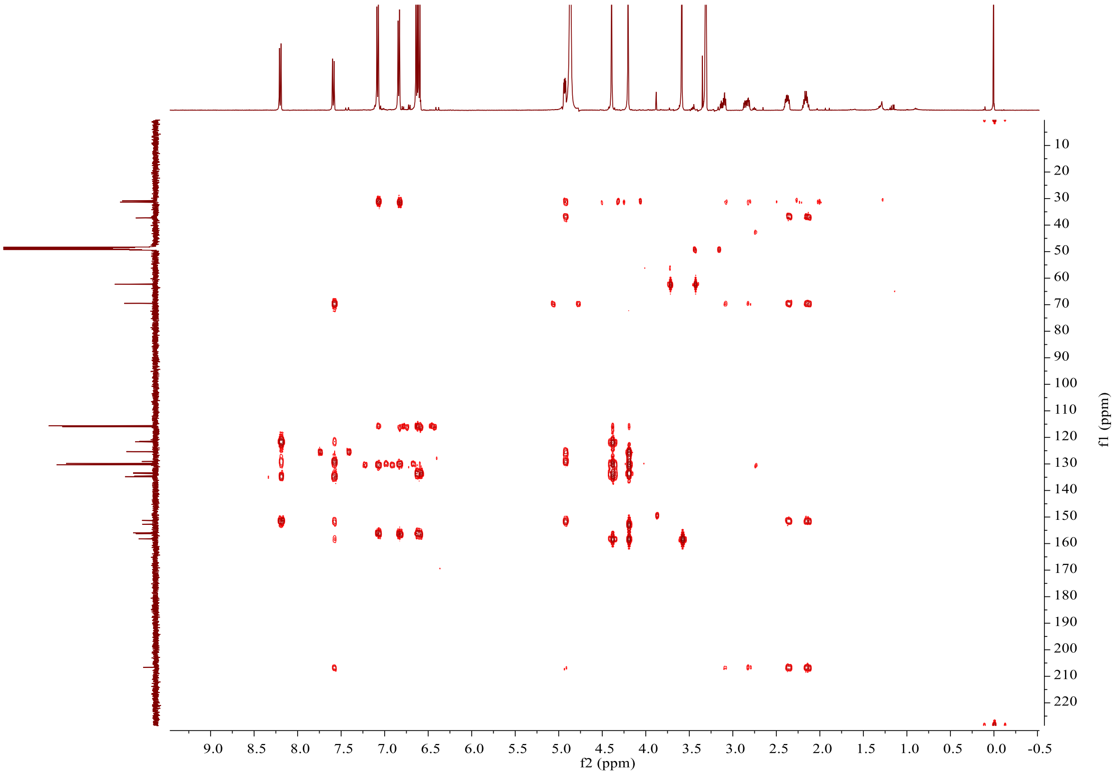

Figure S8. HMBC of compound **1**.

Qualitative Analysis Report

**Data Filename**	ZLZL-156-.d	**Sample Name**	ZLZL-156
**Sample Type**	Sample	**Position**	P1-A1
**Instrument Name**	Instrument 1	**User Name**	
**Acq Method**	s-.m	**Acquired Time**	1/24/2022 10:59:53 AM
**IRM Calibration Status**	Success	**DA Method**	PCDL.m
**Comment**			

Sample Group **Info.**
Acquisition SW Version 6200 series TOF/6500 series Q-TOF B.05.01 (B5125.2)

User Spectra

Fragmentor Voltage	Collision Energy	Ionization Mode
135	0	ESI

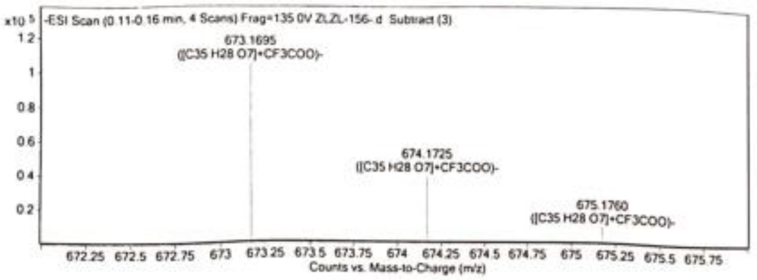

Peak List

m/z	z	Abund	Formula	Ion
160.8417	1	7576.28		
403.3072	1	9868.41		
417.323	1	11325		
453.1348	1	48586.64		
454.1379	1	13806.28		
595.1531	1	13966.08		
622.1719	1	13411.26		
673.1695	1	105208.61	C35 H28 O7	(M+CF3COO)-
674.1725	1	39709.67	C35 H28 O7	(M+CF3COO)-
675.176	1	10236.45	C35 H28 O7	(M+CF3COO)-

Formula Calculator Element Limits

Element	Min	Max
C	3	60
H	0	120
O	0	30

Formula Calculator Results

Formula	CalculatedMass	CalculatedMz	Mz	Diff. (mDa)	Diff. (ppm)	DBE
C35 H28 O7	560.1835	673.1691	673.1695	-0.40	-0.59	22.0000

--- End Of Report ---

Agilent Technologies   Page 1 of 1   Printed at: 11:42 AM on: 1/24/2022

Figure S9. HRESIMS of compound **2**.

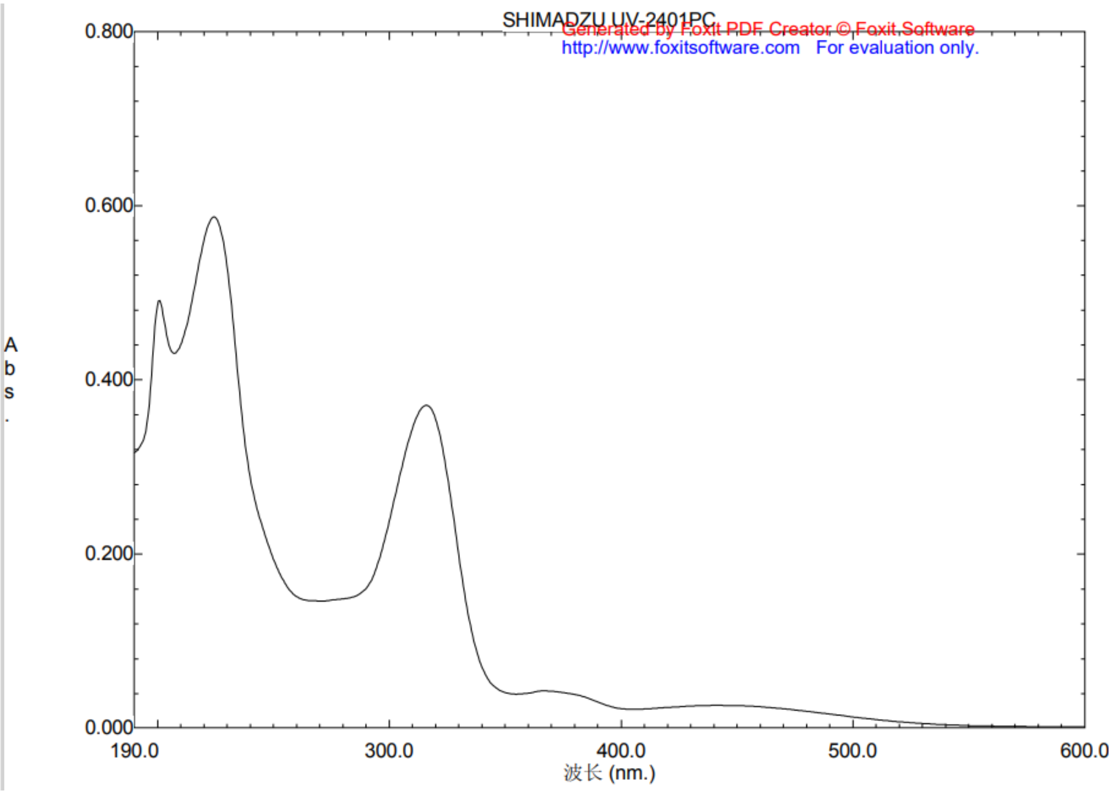

Figure S10. Ultraviolet spectra of compound **2**

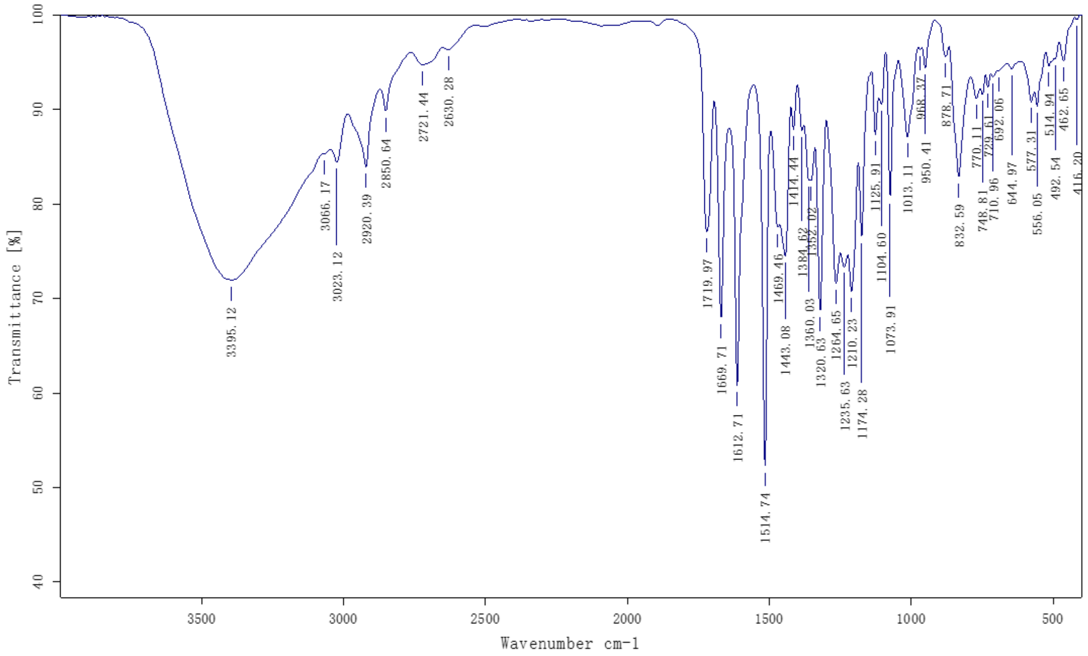

Figure S11. Infrared spectra of compound **2**

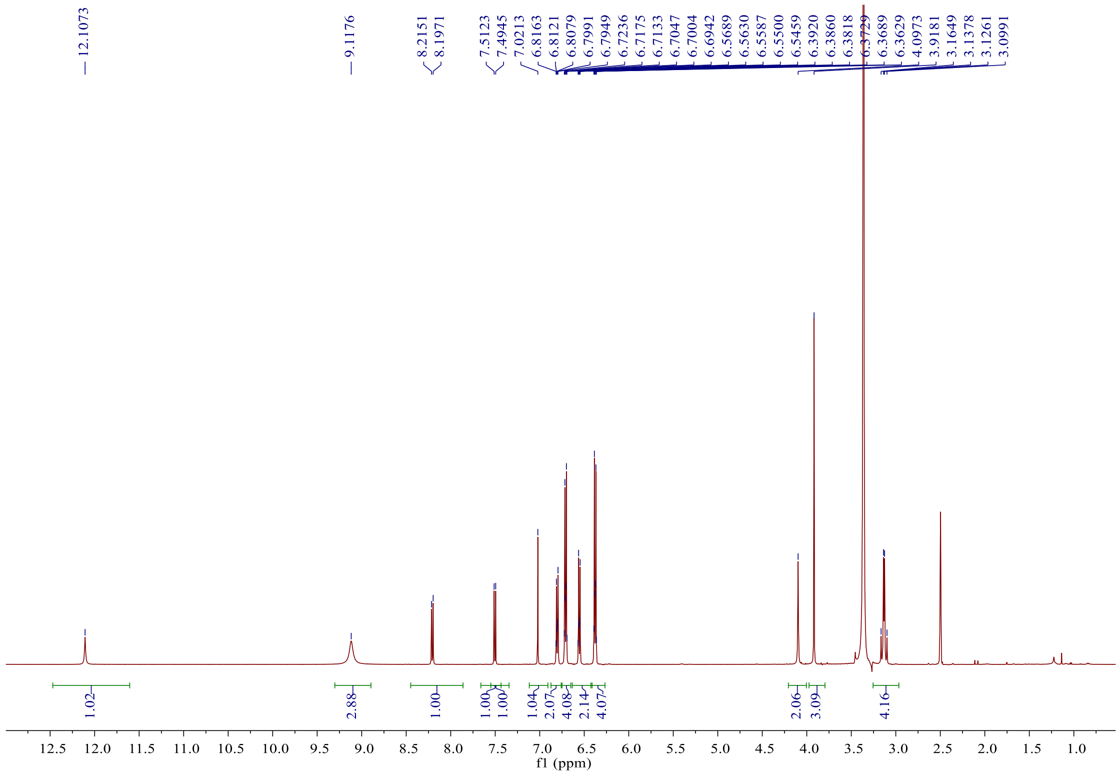

Figure S12. ^{1}H-NMR of compound **2**.

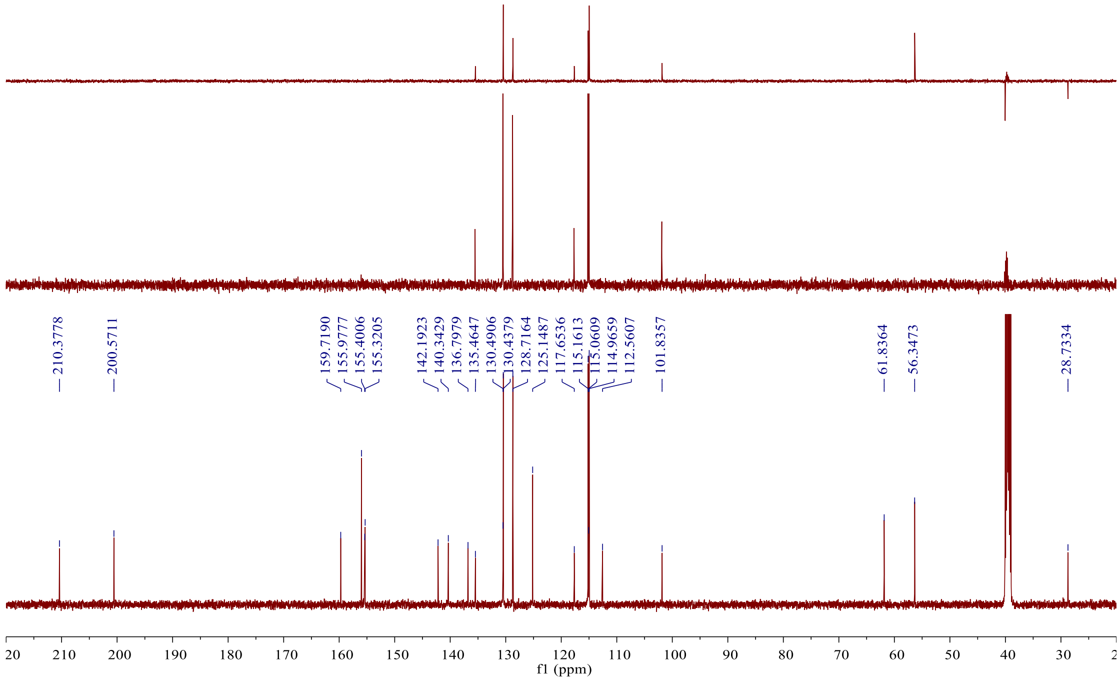

Figure S13. ^{13}C-NMR of compound **2**.

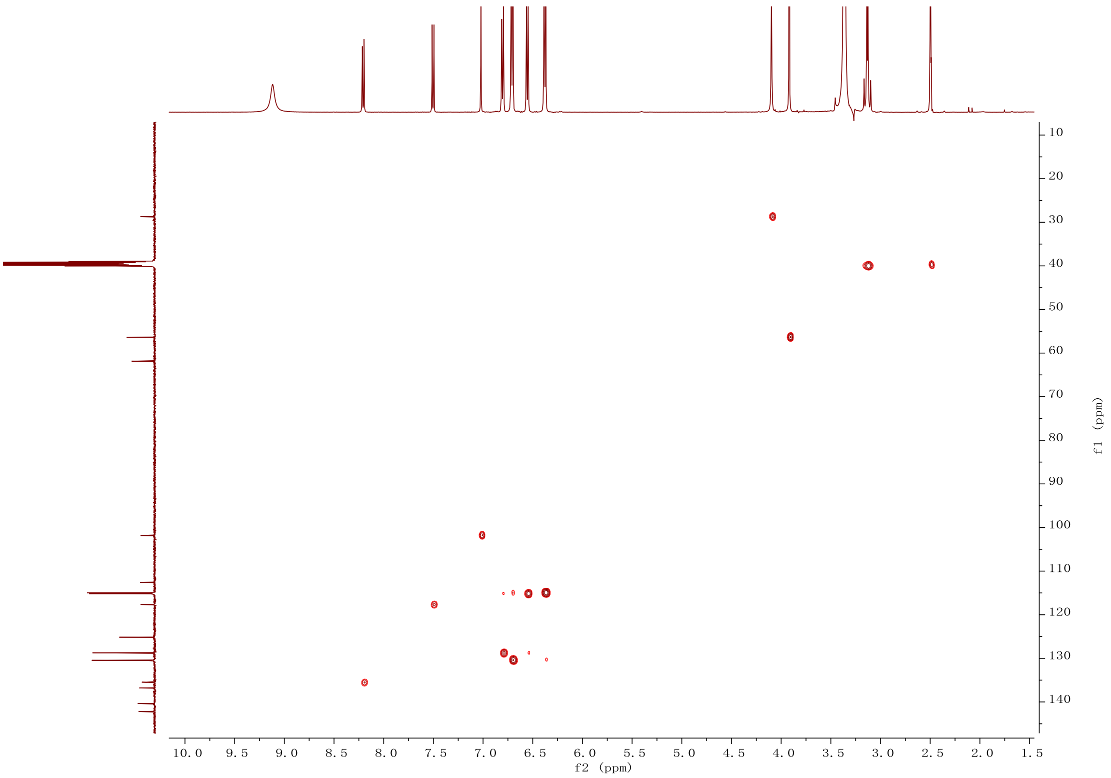

Figure S14. HSQC of compound **2**.

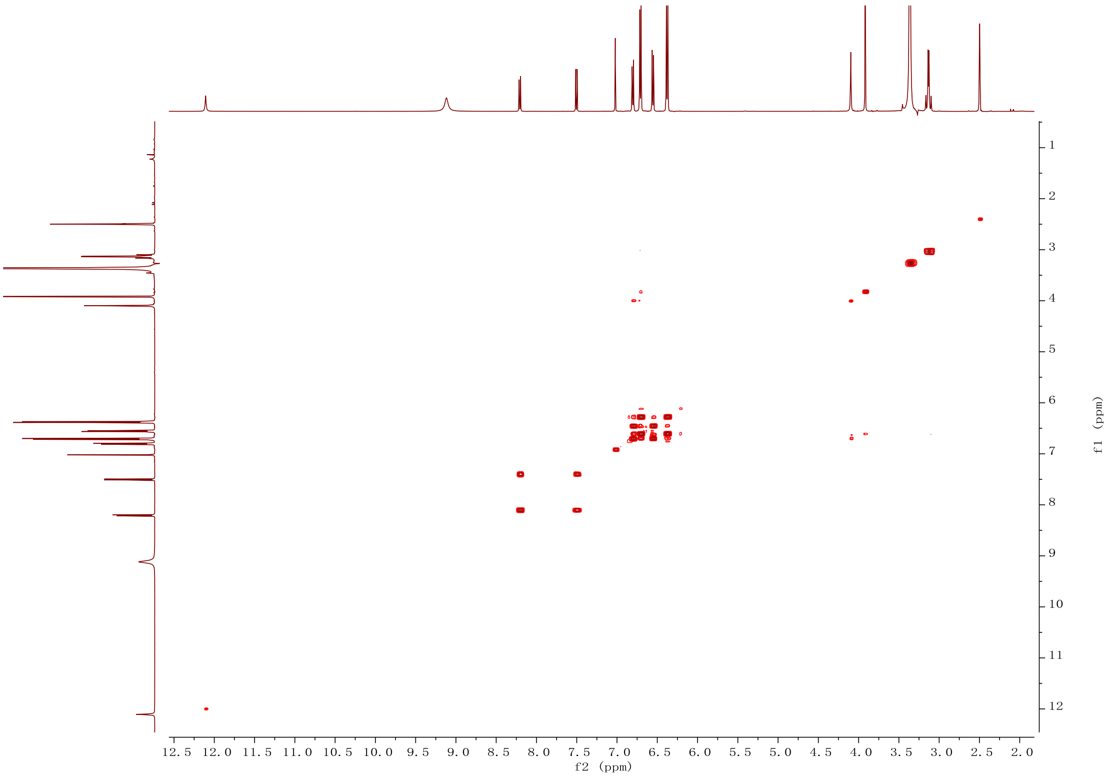

Figure S15. ^{1}H-^{1}H COSY of compound **2**.

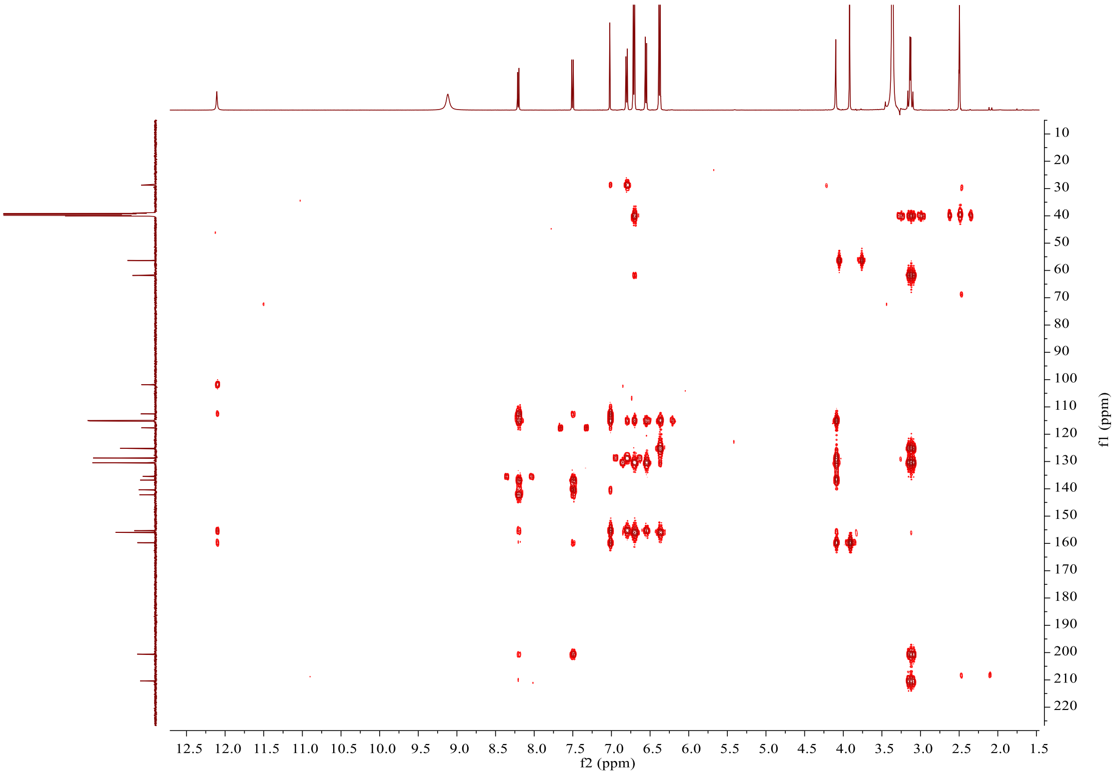

Figure S16. HMBC of compound **2**.

Qualitative Analysis Report

**Data Filename**	ZLZL-161.d	**Sample Name**	ZLZL-161
**Sample Type**	Sample	**Position**	P1-A4
**Instrument Name**	Instrument 1	**User Name**	
**Acq Method**	s-.m	**Acquired Time**	2/21/2022 10:57:31 AM
**IRM Calibration Status**	Success	**DA Method**	PCDL.m
**Comment**			

Sample Group **Info.**

Acquisition SW Version 6200 series TOF/6500 series Q-TOF B.05.01 (B5125.2)

User Spectra

Fragmentor Voltage	Collision Energy	Ionization Mode
135	0	ESI

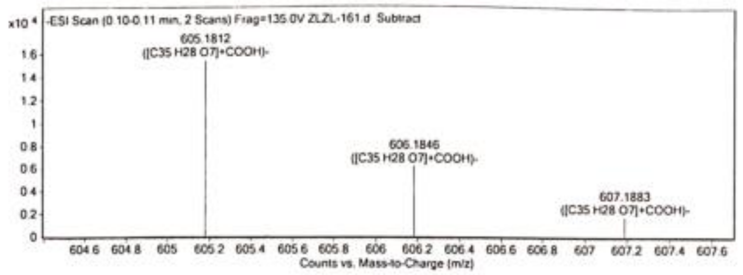

Peak List

m/z	z	Abund	Formula	Ion
112.9855	1	7905.33		
393.2771	1	4894.05		
403.3061	1	9960.41		
453.1337	1	16482.63		
454.1374	1	4956.57		
595.152	1	8572.05		
596.1563	1	4894.06		
605.1812	1	15327.76	C35 H28 O7	(M+COOH)-
606.1846	1	6108.17	C35 H28 O7	(M+COOH)-
966.0005	1	6880.27		

Formula Calculator Element Limits

Element	Min	Max
C	3	60
H	0	120
O	0	30

Formula Calculator Results

Formula	CalculatedMass	CalculatedMz	Mz	Diff. (mDa)	Diff. (ppm)	DBE
C35 H28 O7	560.1835	605.1817	605.1812	0.50	0.83	22.0000

--- End Of Report ---

Agilent Technologies Page 1 of 1 Printed at: 10:16 AM on: 2/23/2022

Figure S17. HRESIMS of compound **3**.

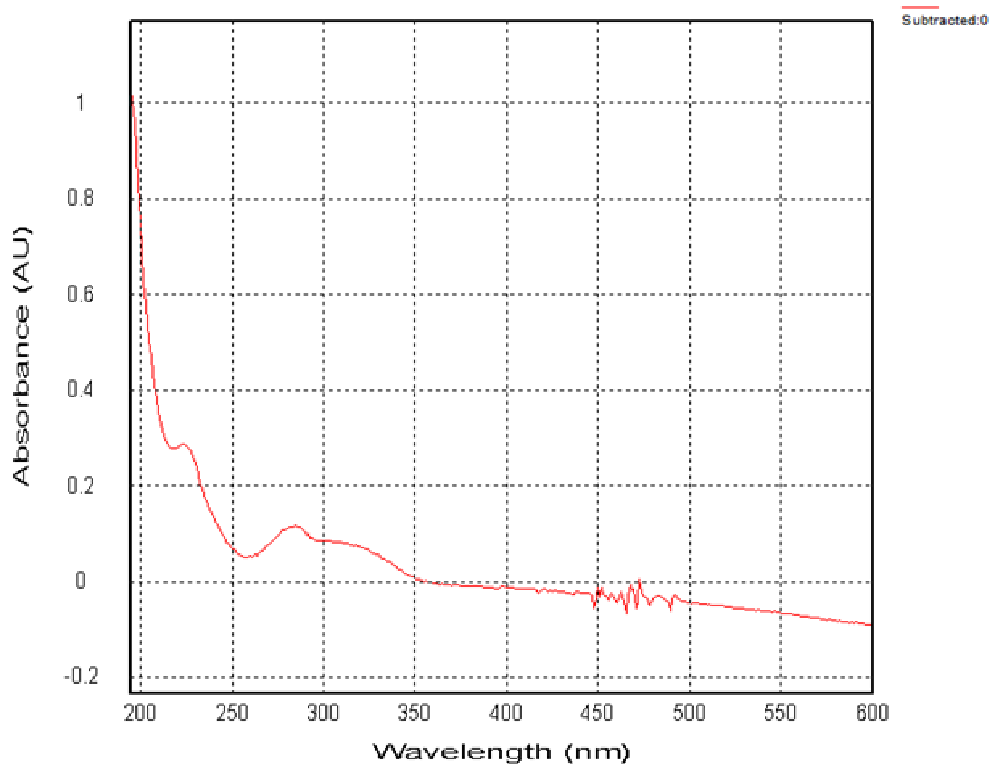

Figure S18. Ultraviolet spectra of compound **3**.

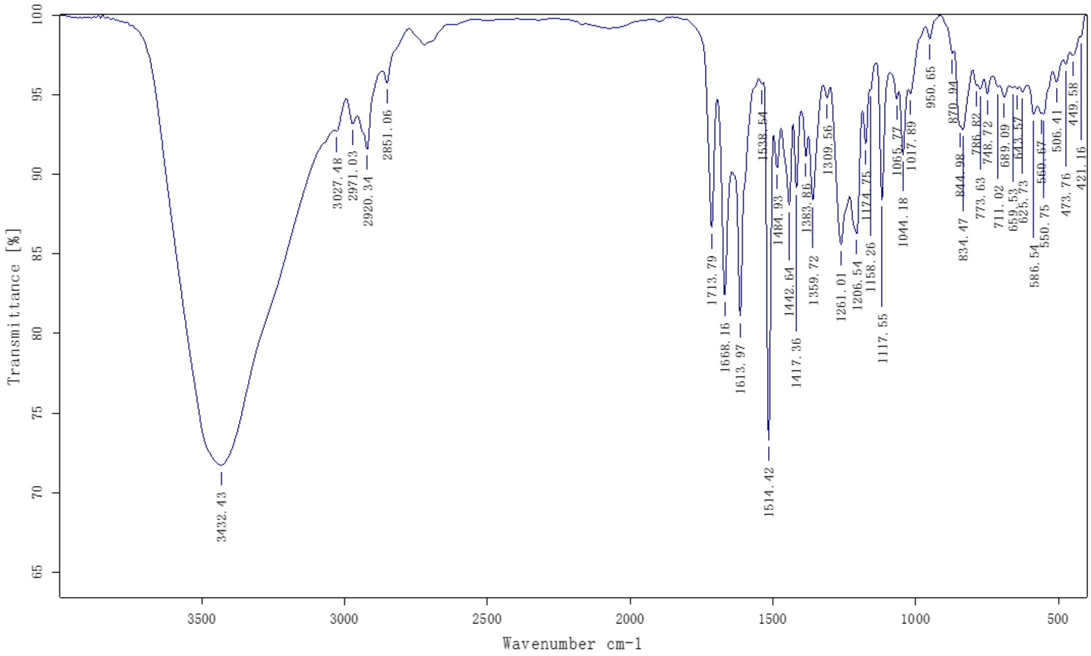

Figure S19. Infrared spectra of compound **3**.

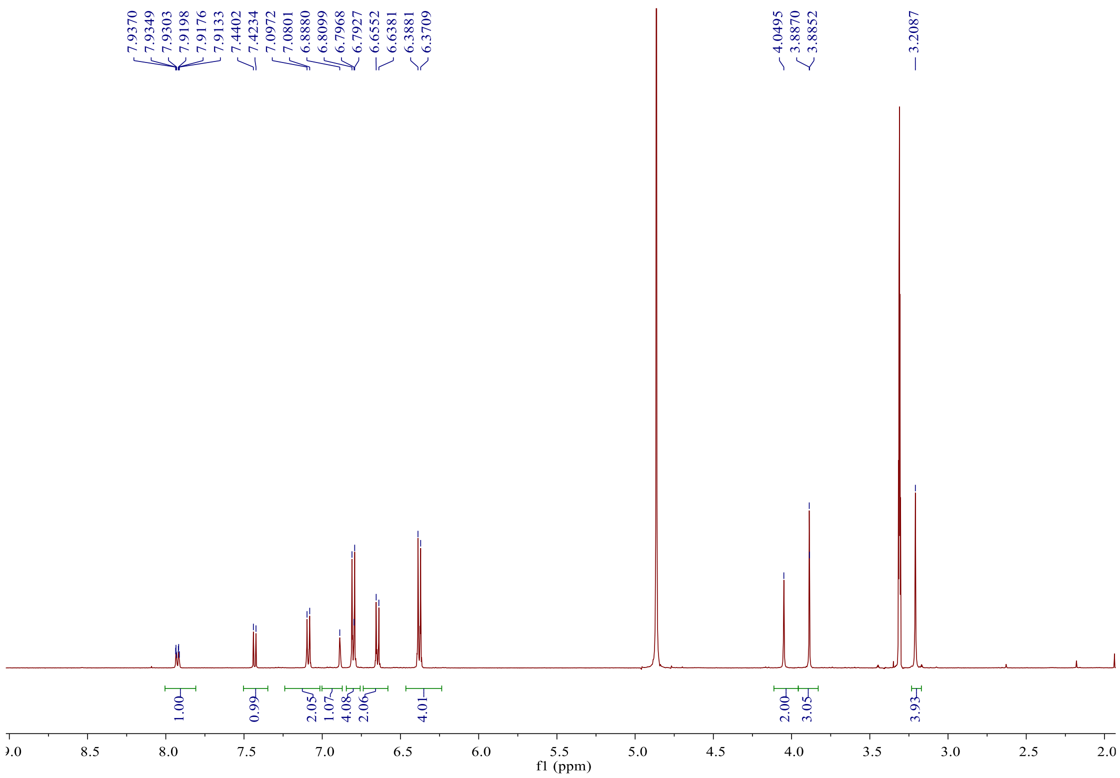

Figure S20. ^{1}H-NMR of compound **3**.

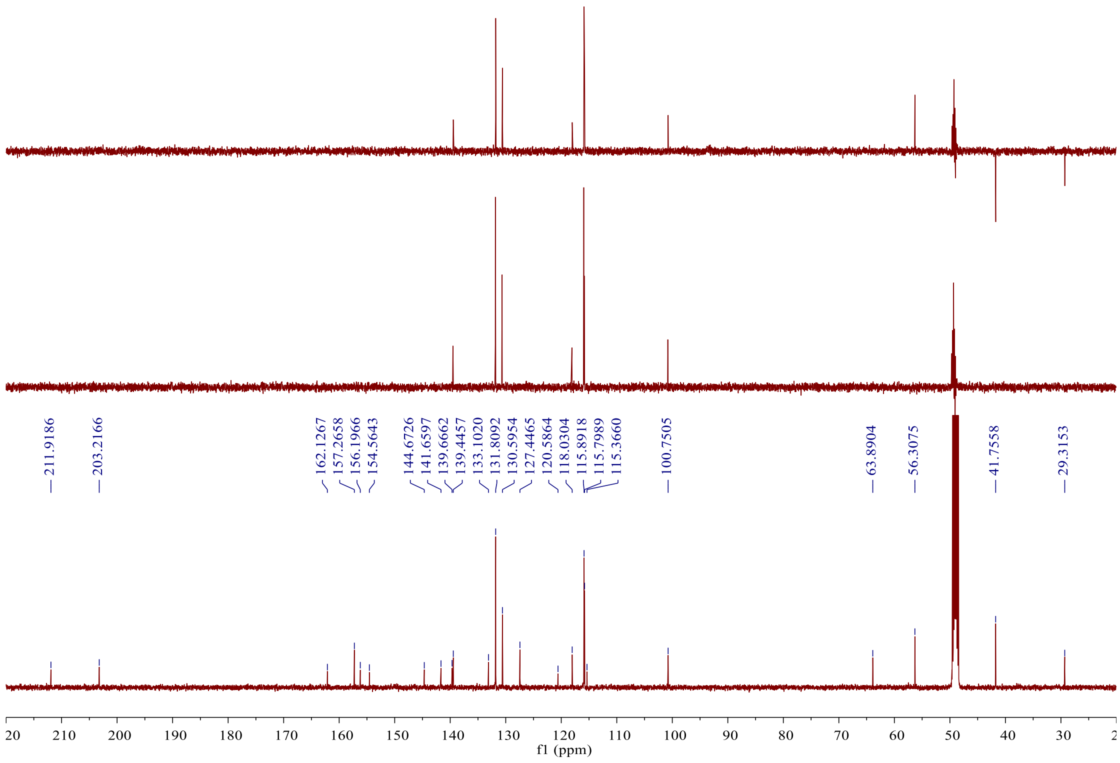

Figure S21. ^{13}C-NMR of compound **3**.

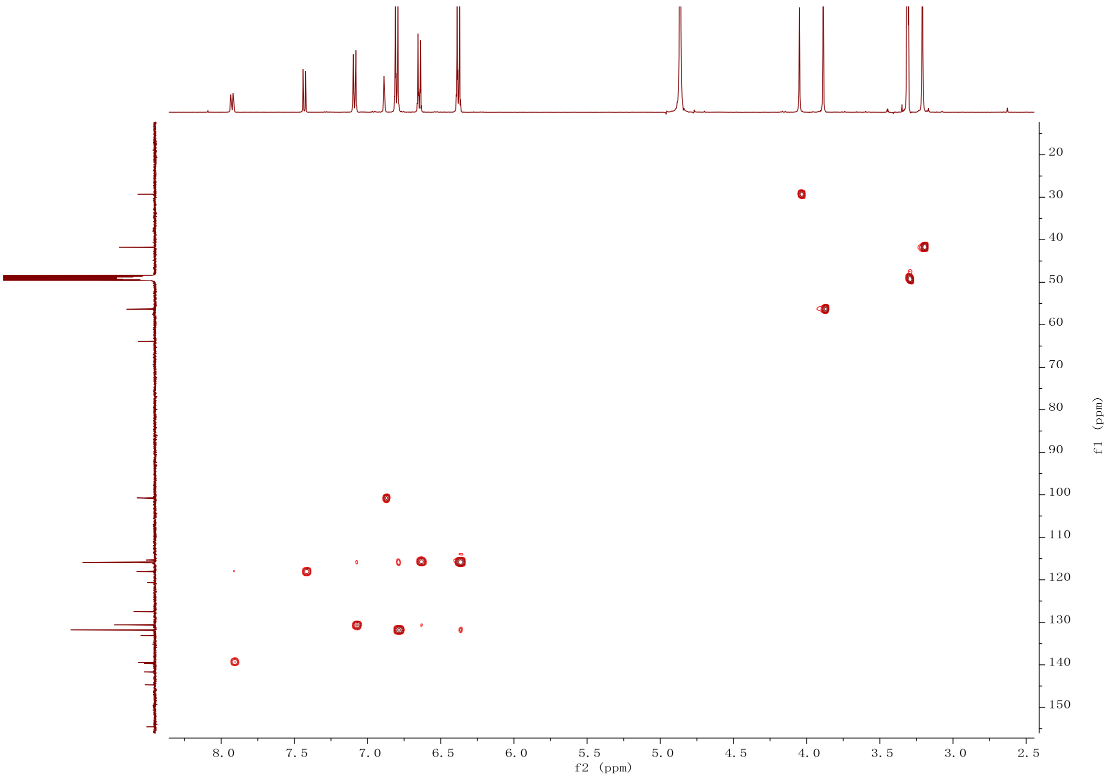

Figure S22. HSQC of compound **3**.

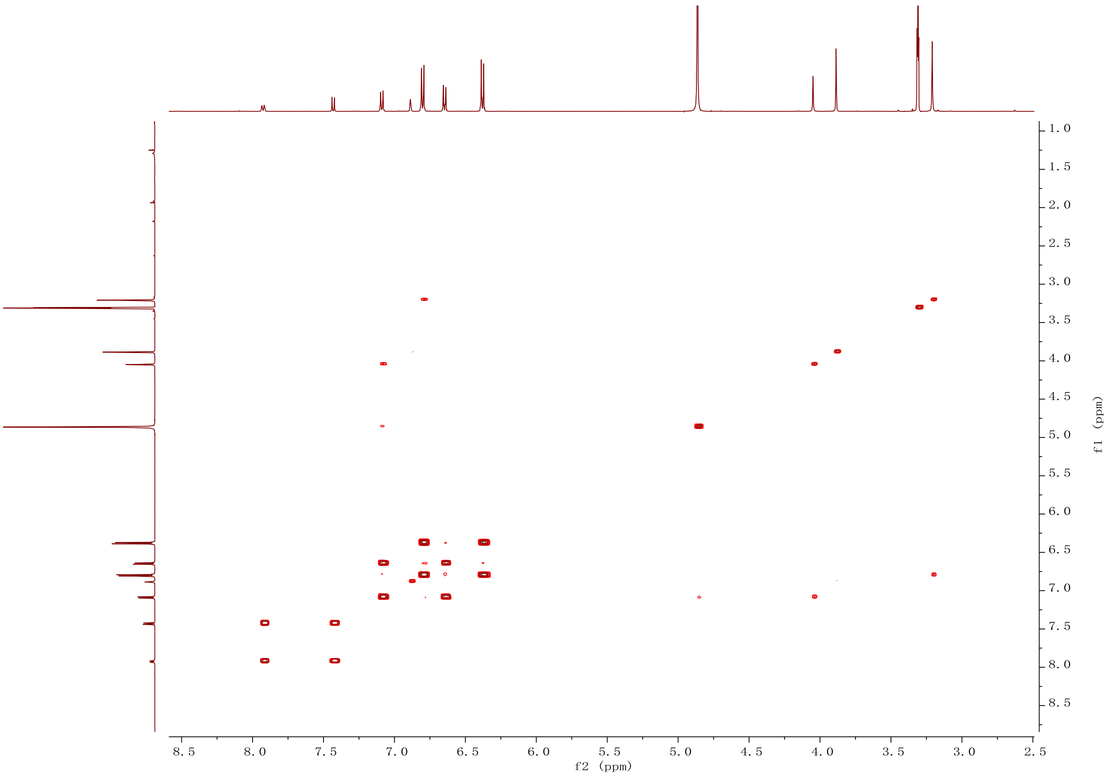

Figure S23. ^1H-^1H COSY of compound **3**.

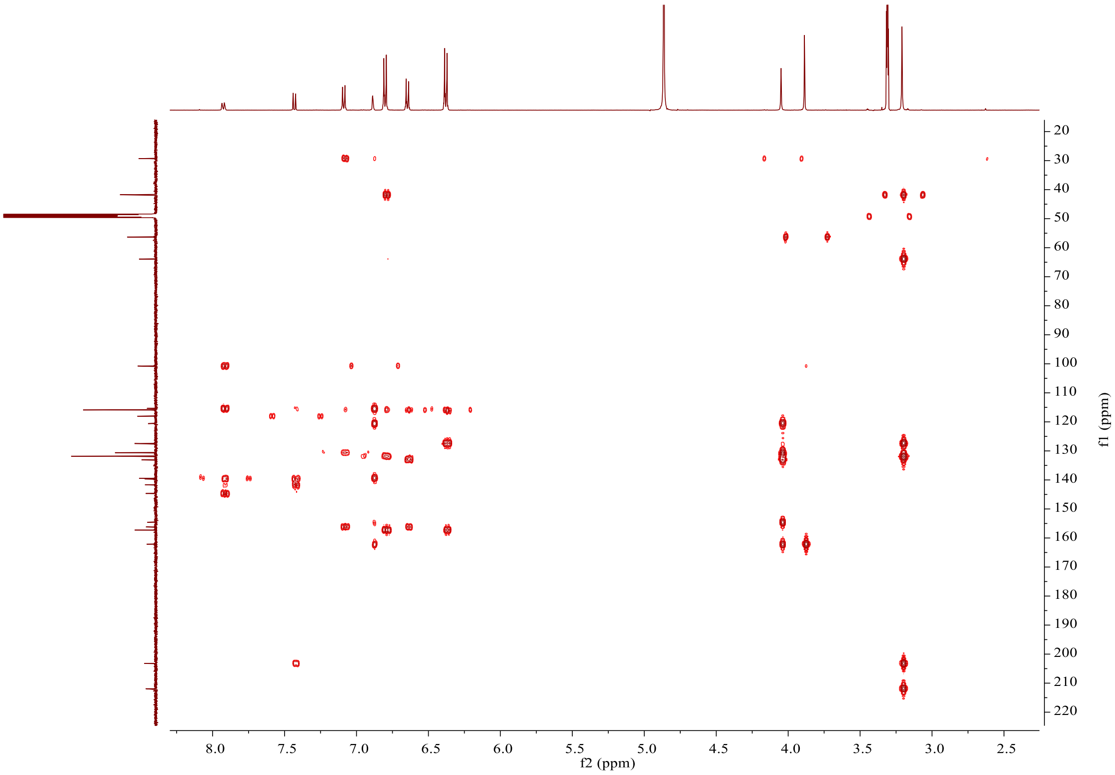

Figure S24. HMBC of compound **3**.

Qualitative Analysis Report

**Data Filename**	ZLZL-162.d	**Sample Name**	ZLZL-162
**Sample Type**	Sample	**Position**	P1-A1
**Instrument Name**	Instrument 1	**User Name**	
**Acq Method**	s-.m	**Acquired Time**	2/23/2022 4:50:12 PM
**IRM Calibration Status**	Success	**DA Method**	PCDL.m
**Comment**			

Sample Group **Info.**
Acquisition SW Version 6200 series TOF/6500 series Q-TOF B.05.01 (B5125.2)

User Spectra

Fragmentor Voltage	Collision Energy	Ionization Mode
135	0	ESI

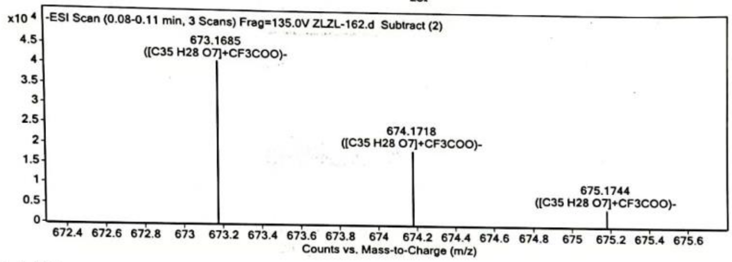

Peak List

m/z	z	Abund	Formula	Ion
453.1335	1	2719.11		
595.1522	1	5118.83		
596.1548	1	1799.25		
605.1801	1	6608.64		
606.1851	1	2744.7		
622.1706	1	7248.44		
623.1748	1	2736.77		
673.1685	1	41006.46	C35 H28 O7	(M+CF3COO)-
674.1718	1	18843.85	C35 H28 O7	(M+CF3COO)-
675.1744	1	4457.79	C35 H28 O7	(M+CF3COO)-

Formula Calculator Element Limits

Element	Min	Max
C	3	60
H	0	200
O	0	30

Formula Calculator Results

Formula	CalculatedMass	CalculatedMz	Mz	Diff. (mDa)	Diff. (ppm)	DBE
C35 H28 O7	560.1835	673.1691	673.1685	0.60	0.89	22.0000

--- End Of Report ---

Figure S25. HRESIMS of compound **4**.

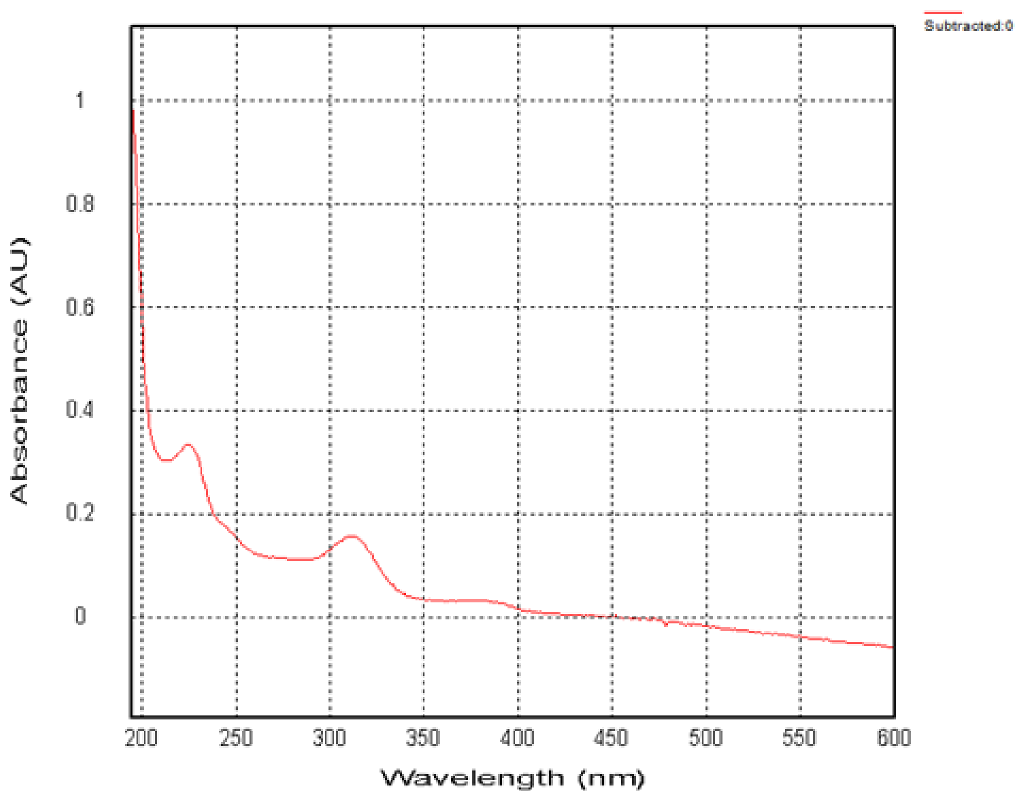

Figure S26. Ultraviolet spectra of compound **4.**

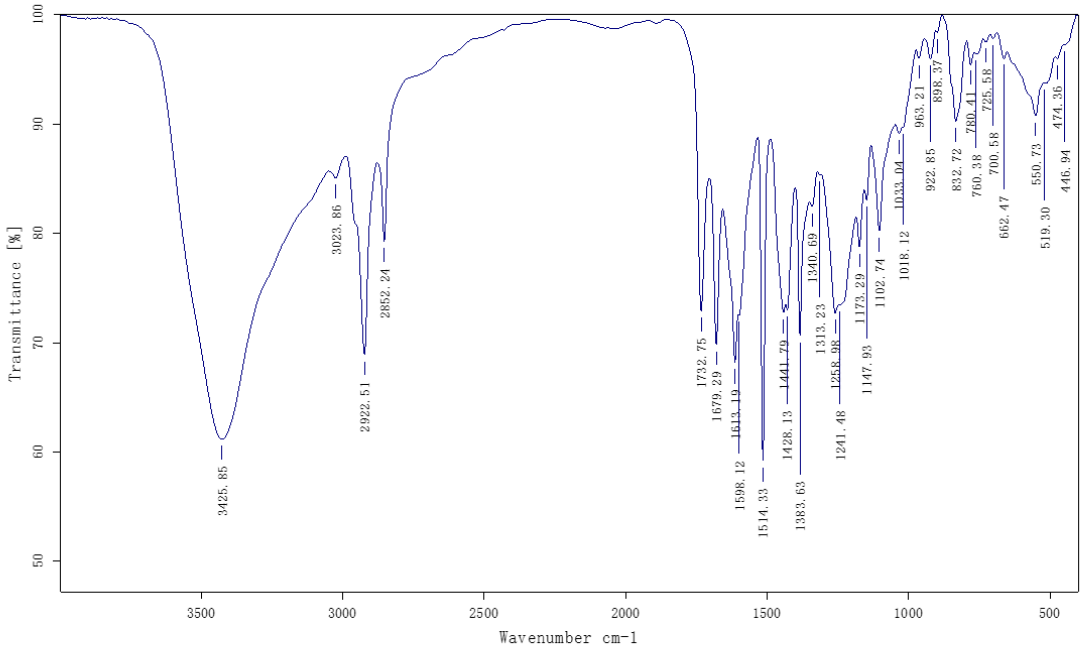

Figure S27. Infrared spectra of compound **4**.

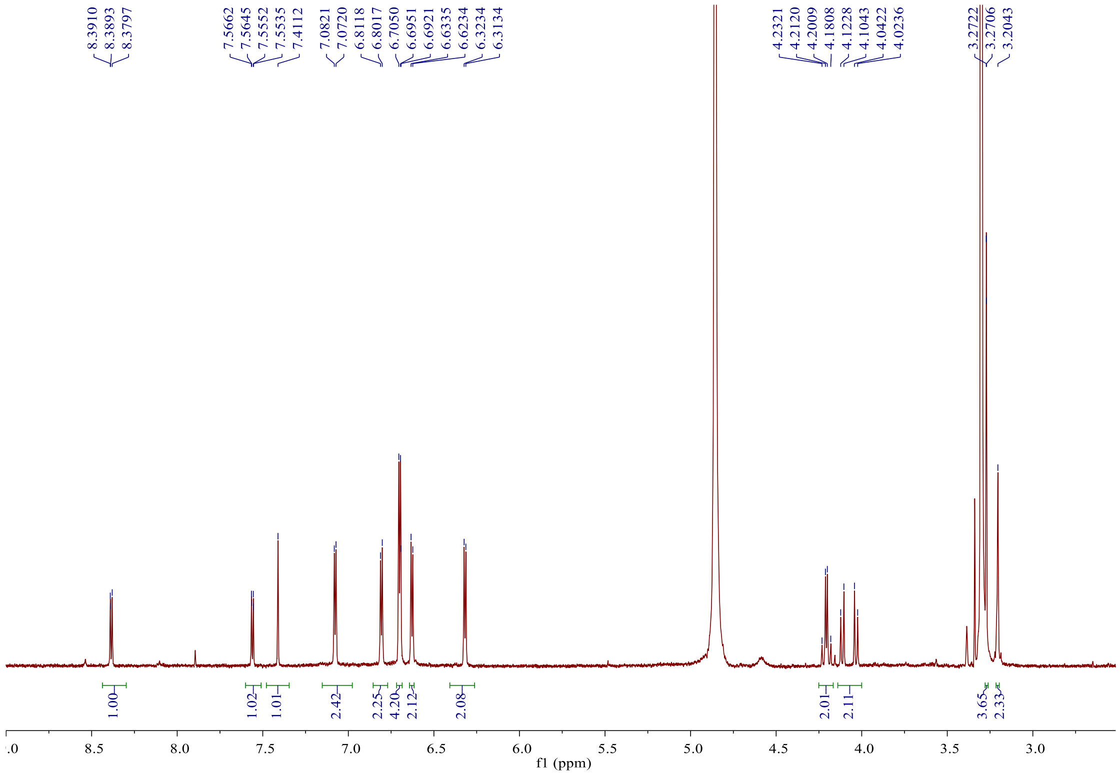

Figure S28. ^{1}H-NMR of compound **4**.

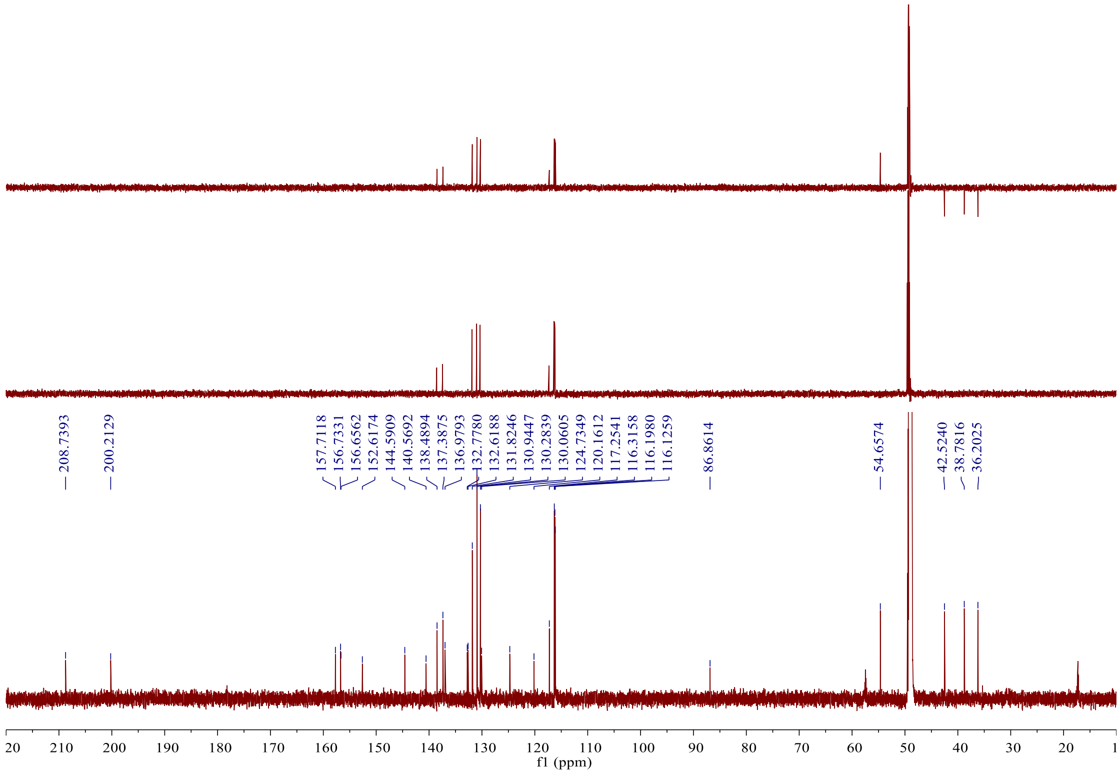

Figure S29. ^{13}C-NMR of compound **4**.

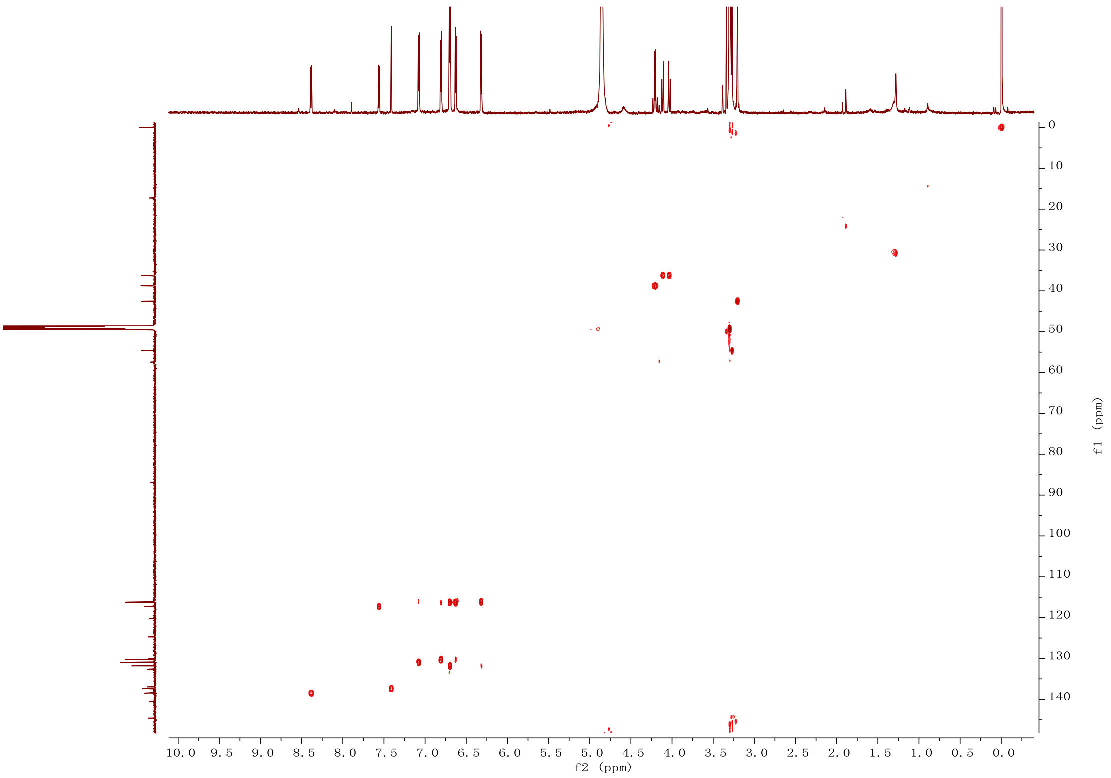

Figure S30. HSQC of compound **4**.

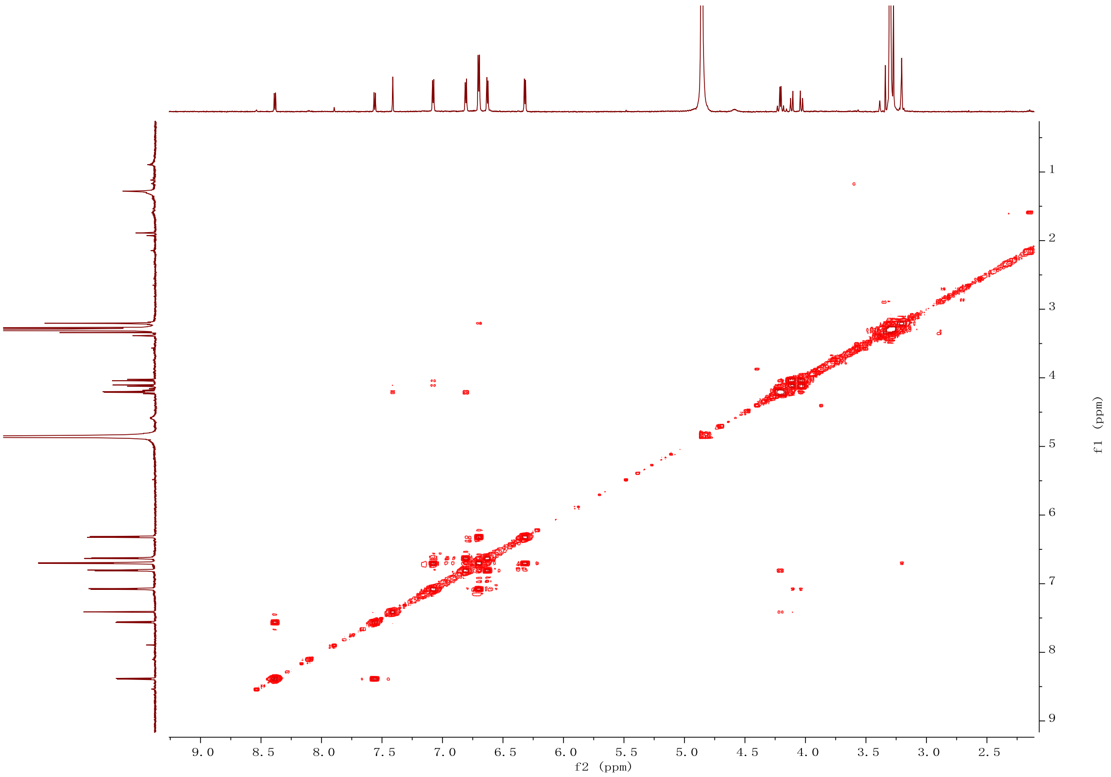

Figure S31. ^{1}H-^{1}H COSY of compound **4**.

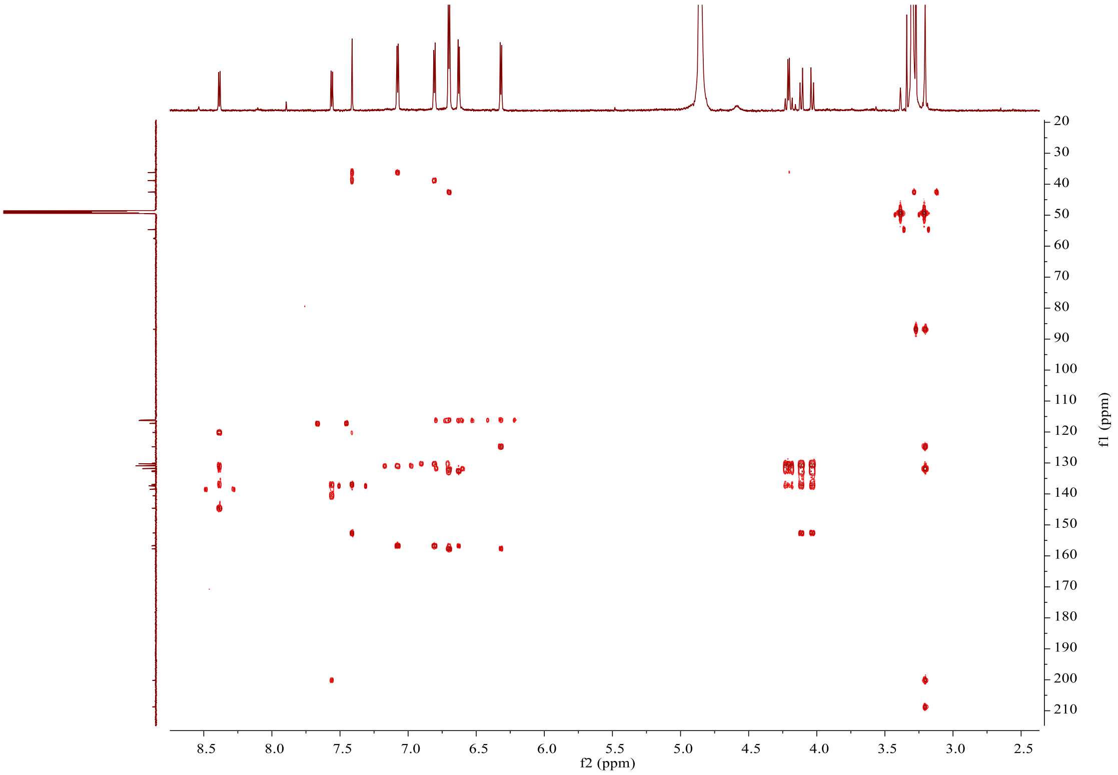

Figure S32. HMBC of compound **4**.

Qualitative Analysis Report

**Data Filename**	ZLZL-14.d	**Sample Name**	ZLZL-14
**Sample Type**	Sample	**Position**	P1-B5
**Instrument Name**	Instrument 1	**User Name**	
**Acq Method**	s-.m	**Acquired Time**	12/8/2021 3:17:43 PM
**IRM Calibration Status**	Success	**DA Method**	PCDL.m
**Comment**			

Sample Group **Info.**
Acquisition SW Version 6200 series TOF/6500 series Q-TOF B.05.01 (B5125.2)

User Spectra

Fragmentor Voltage	Collision Energy	Ionization Mode
135	0	ESI

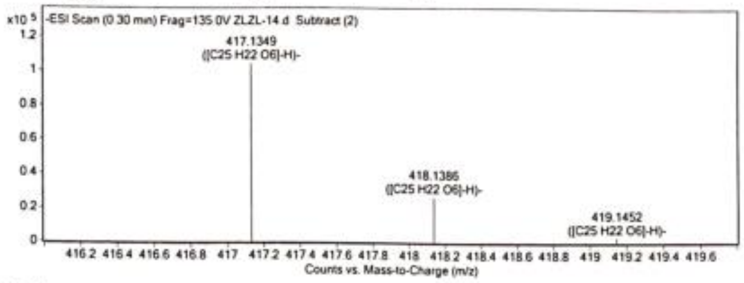

Peak List

m/z	z	Abund	Formula	Ion
283.0982	1	4470.12		
417.1349	1	105050.33	C25 H22 O6	(M-H)-
418.1386	1	27138.37	C25 H22 O6	(M-H)-
419.1452	1	3175.45	C25 H22 O6	(M-H)-
480.1289	1	5691.53		
485.1221	1	9074.04		
515.1123	1	5922.96		
531.127	1	12012.15		
532.1312	1	4971.14		
537.0926	1	4226.41		

Formula Calculator Element Limits

Element	Min	Max
C	3	120
H	0	240
O	0	30

Formula Calculator Results

Formula	CalculatedMass	CalculatedMz	Mz	Diff. (mDa)	Diff. (ppm)	DBE
C25 H22 O6	418.1416	417.1344	417.1349	-0.50	-1.20	15.0000

--- End Of Report ---

Figure S33. HRESIMS of compound **5**.

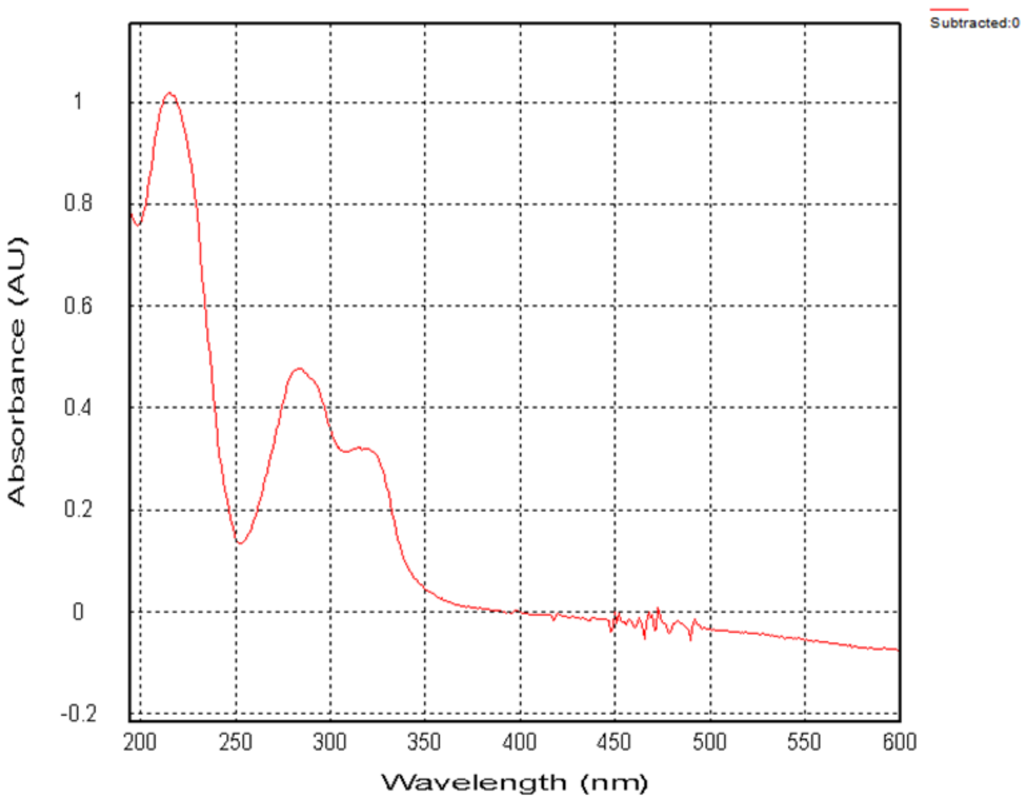

Figure S34. Ultraviolet spectra of compound **5.**

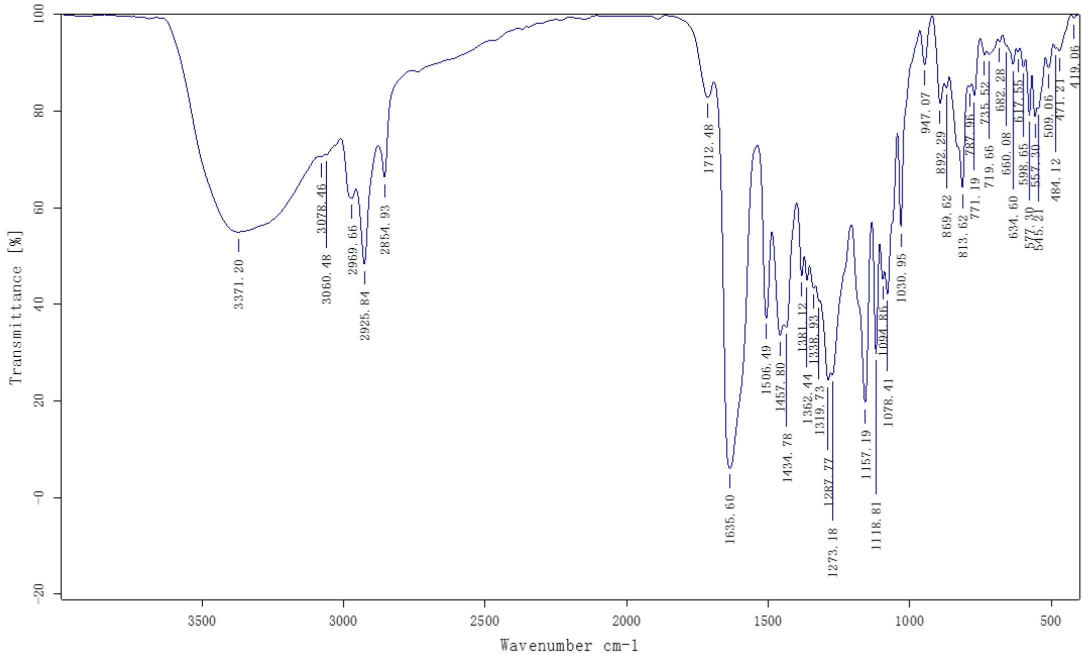

Figure S35. Infrared spectra of compound **5.**

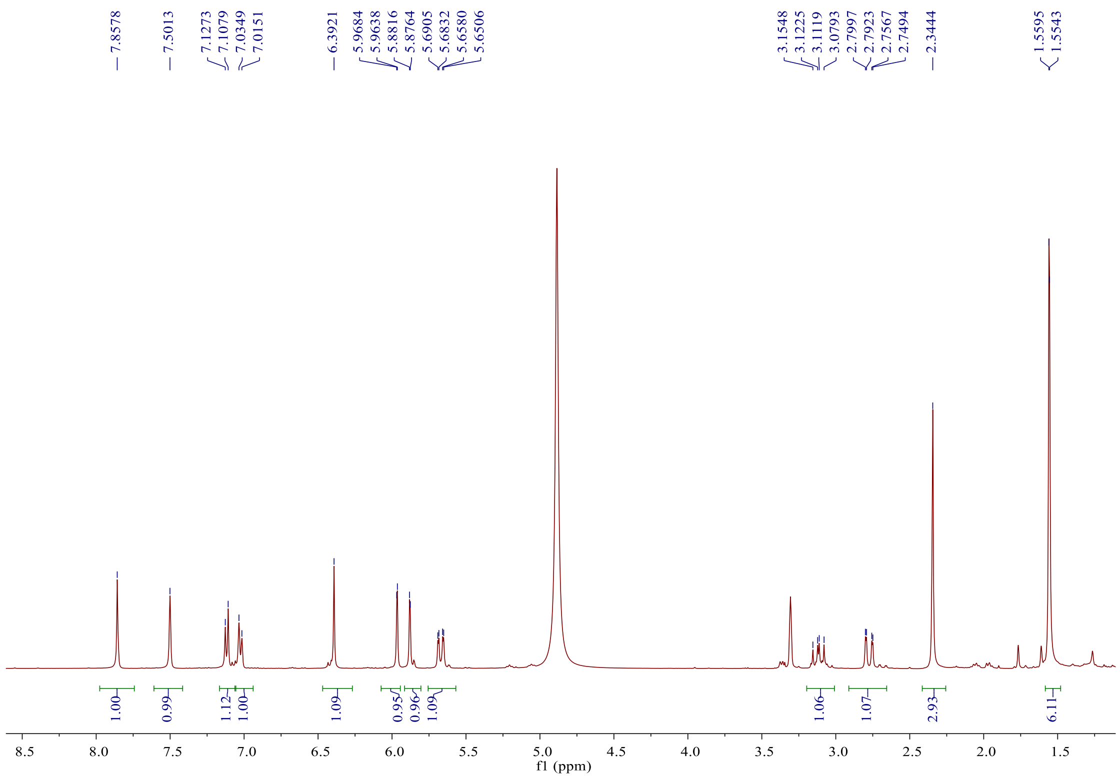

Figure S36. ^{1}H-NMR of compound **5**.

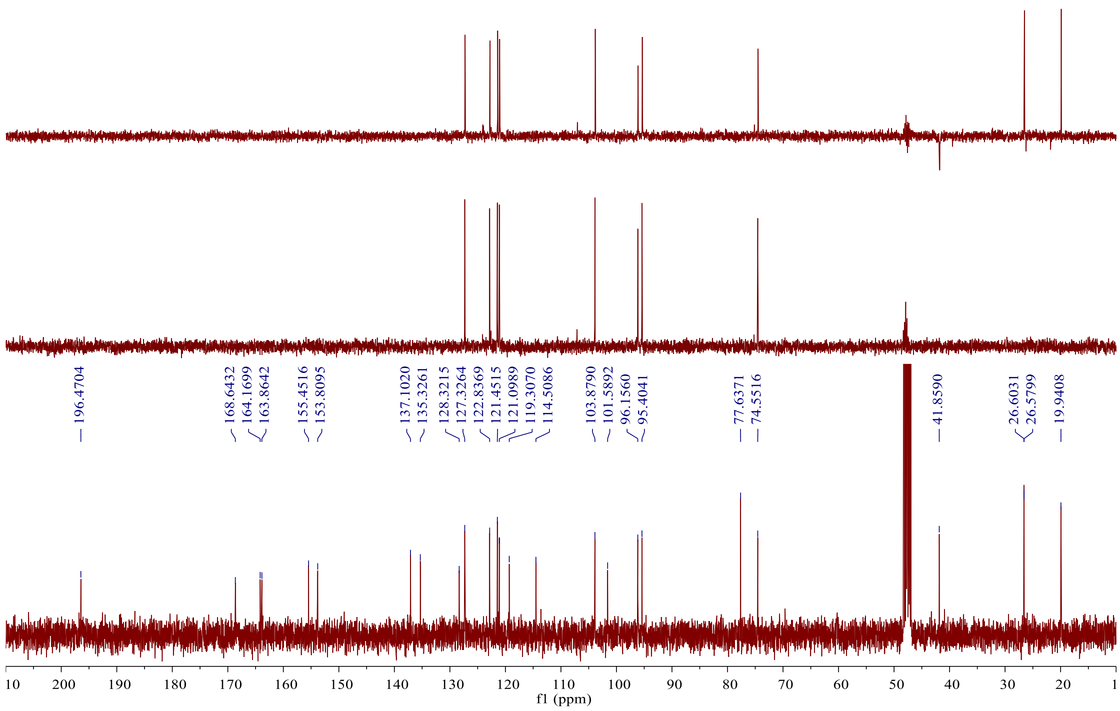

Figure S37. ^{13}C-NMR of compound **5**.

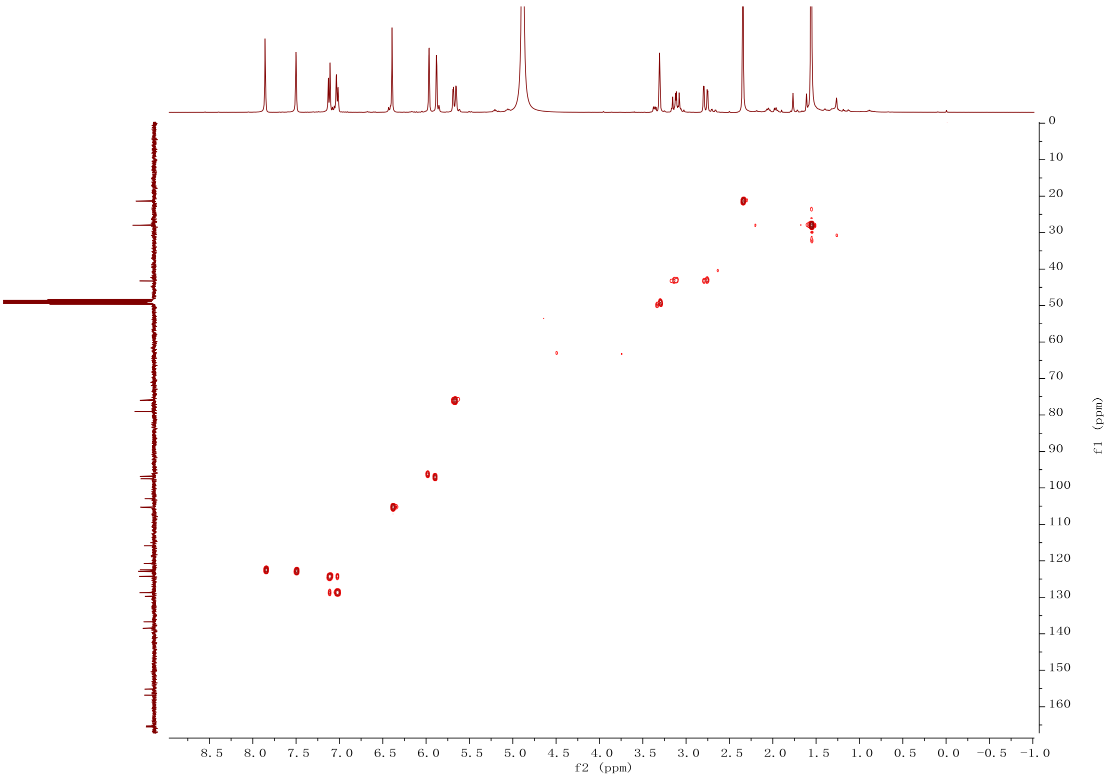

Figure S38. HSQC of compound **5**.

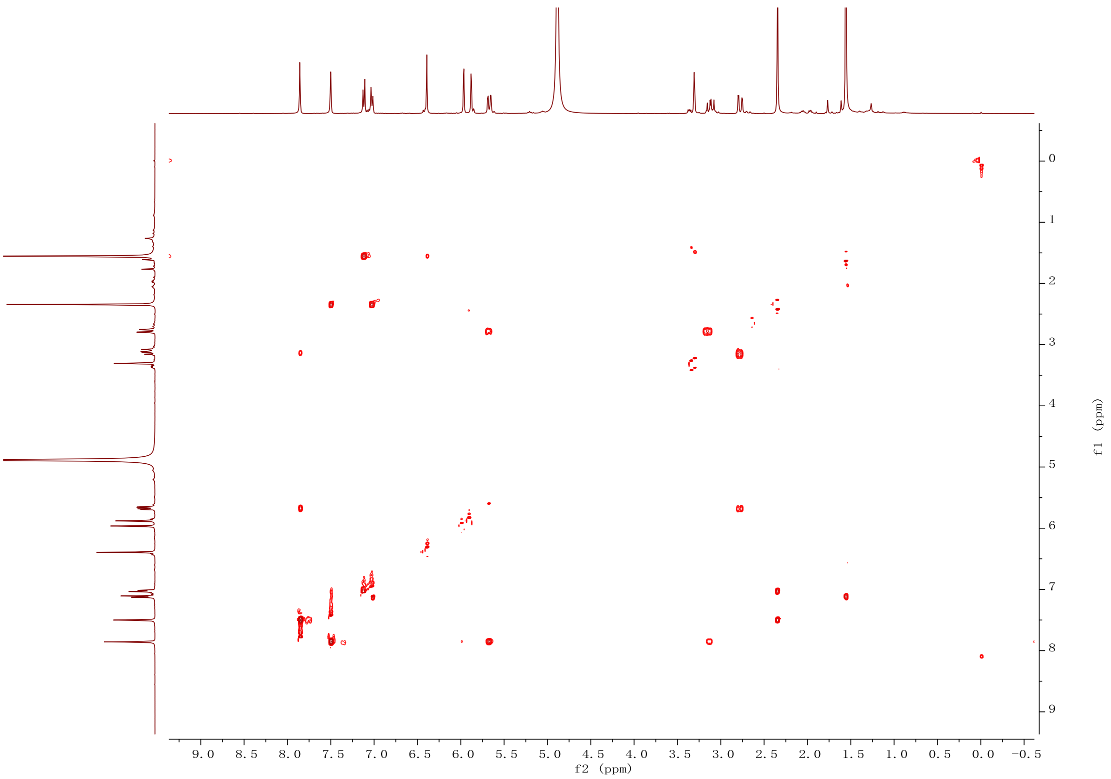

Figure S39. ^{1}H-^{1}H COSY of compound **5**.

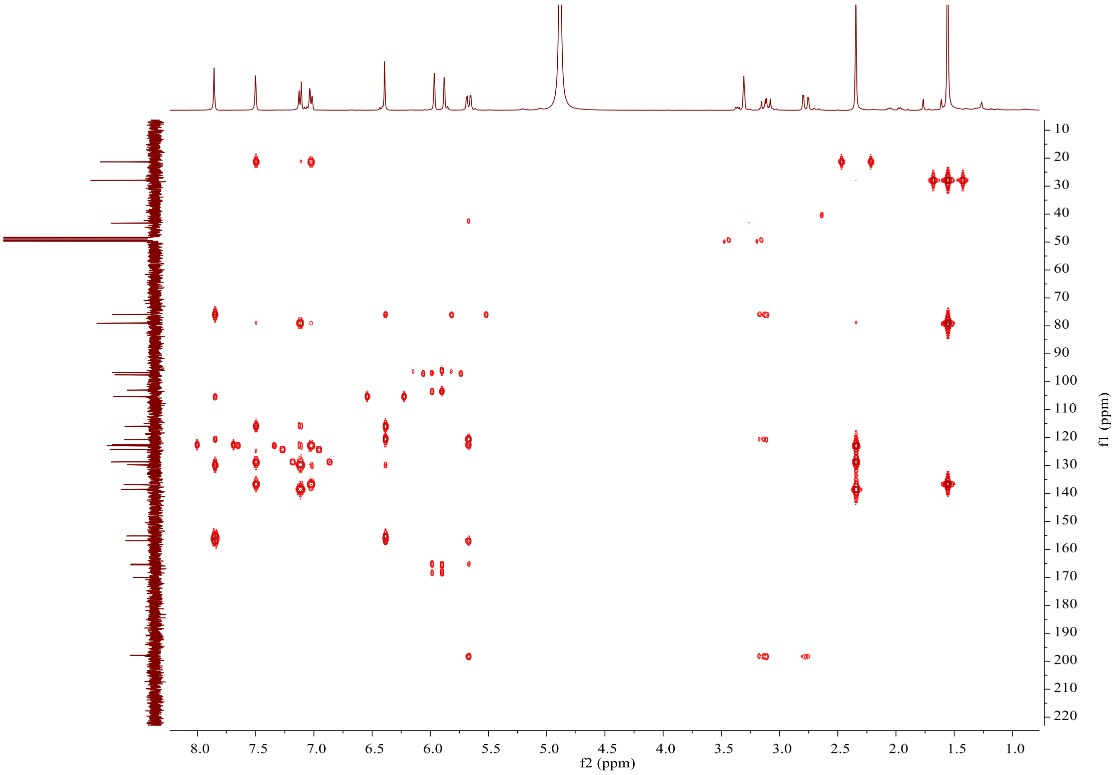

Figure S40. HMBC of compound **5**.

Qualitative Analysis Report

**Data Filename**	ZLZL-95d.d	**Sample Name**	ZLZL-95
**Sample Type**	Sample	**Position**	P1-B7
**Instrument Name**	Instrument 1	**User Name**	
**Acq Method**	s-.m	**Acquired Time**	12/8/2021 3:20:06 PM
**IRM Calibration Status**	Success	**DA Method**	PCDL.m
**Comment**			

Sample Group **Info.**
Acquisition SW Version 6200 series TOF/6500 series Q-TOF B.05.01 (B5125.2)

User Spectra

Fragmentor Voltage	Collision Energy	Ionization Mode
135	0	ESI

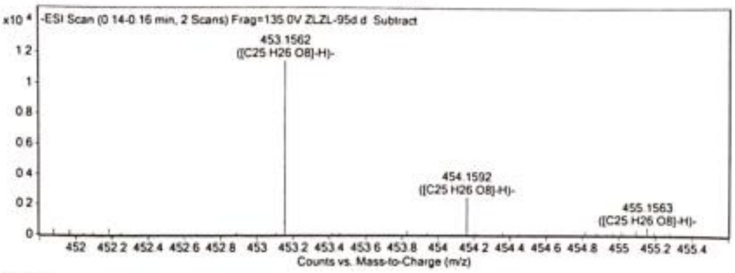

Peak List

m/z	z	Abund	Formula	Ion
89.0244	1	21881.15		
154.9736	1	5396.93		
248.961	1	5111.55		
453.1562	1	11405.68	C25 H26 O8	(M-H)-
955.971	1	20804.62		
956.9739	1	5463.54		
957.9687	1	8340.22		
1010.025	1	4588.8		
1033.9881	1	67414.75		
1034.9907	1	11134.63		

Formula Calculator Element Limits

Element	Min	Max
C	3	120
H	0	240
O	0	30

Formula Calculator Results

Formula	CalculatedMass	CalculatedMz	Mz	Diff. (mDa)	Diff. (ppm)	DBE
C25 H26 O8	454.1628	453.1555	453.1562	-0.70	-1.54	13.0000

--- End Of Report ---

Agilent Technologies Page 1 of 1 Printed at: 3:21 PM on: 12/8/2021

Figure S41. HRESIMS of compound **6**.

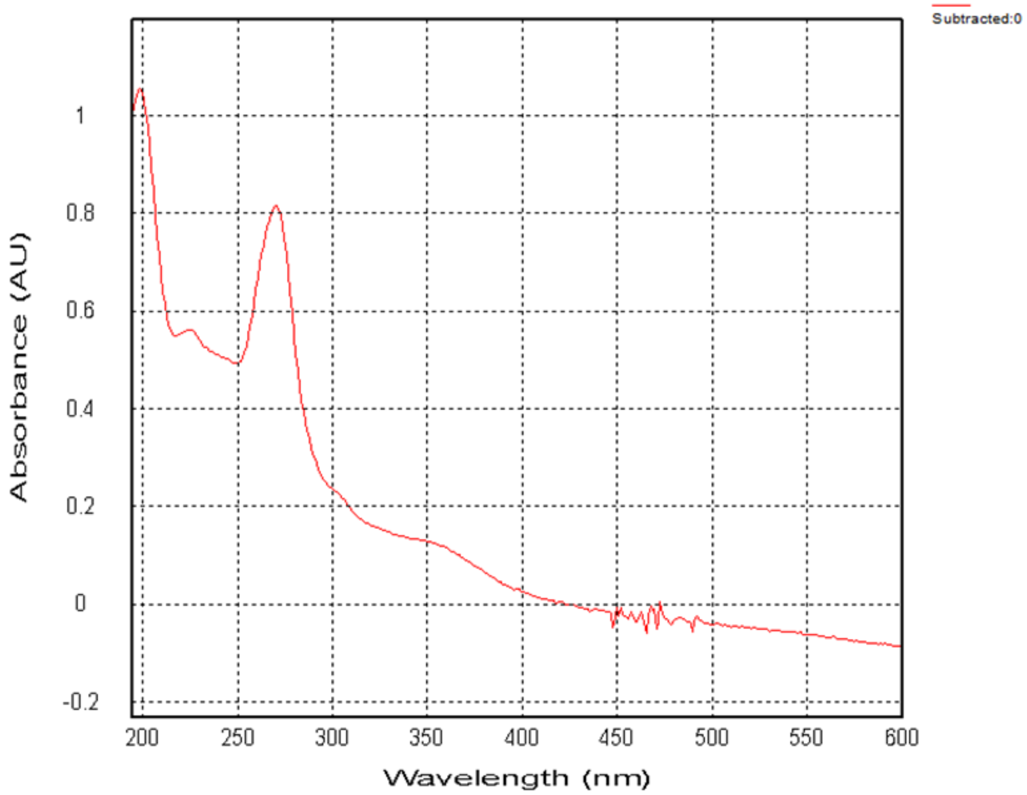

Figure S42. Ultraviolet spectra of compound **6.**

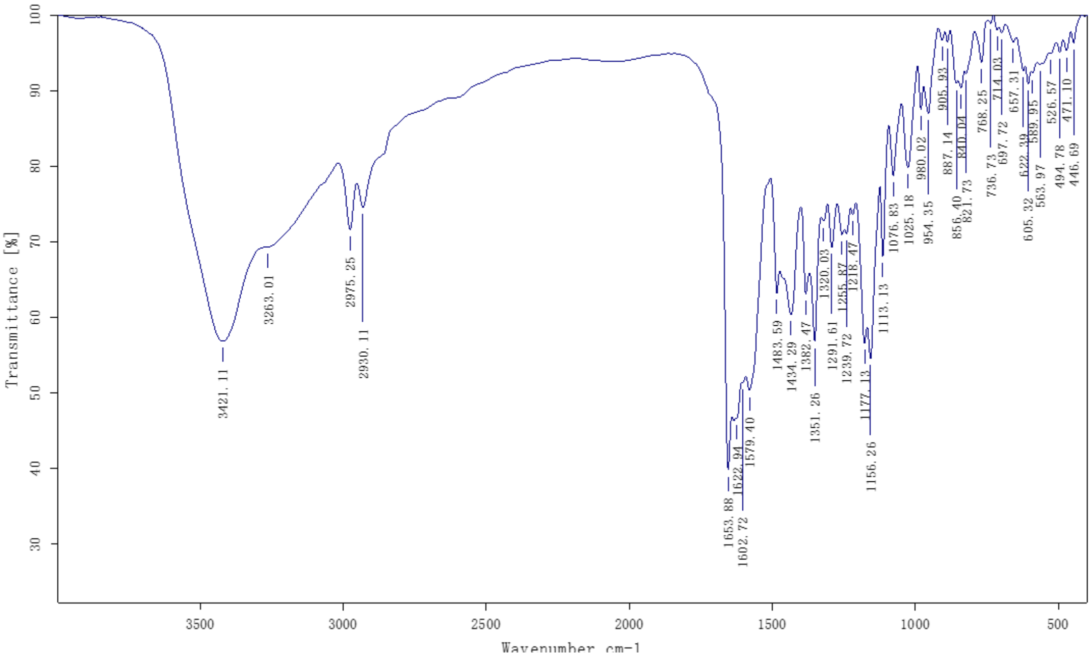

Figure S43. Infrared spectra of compound **6.**

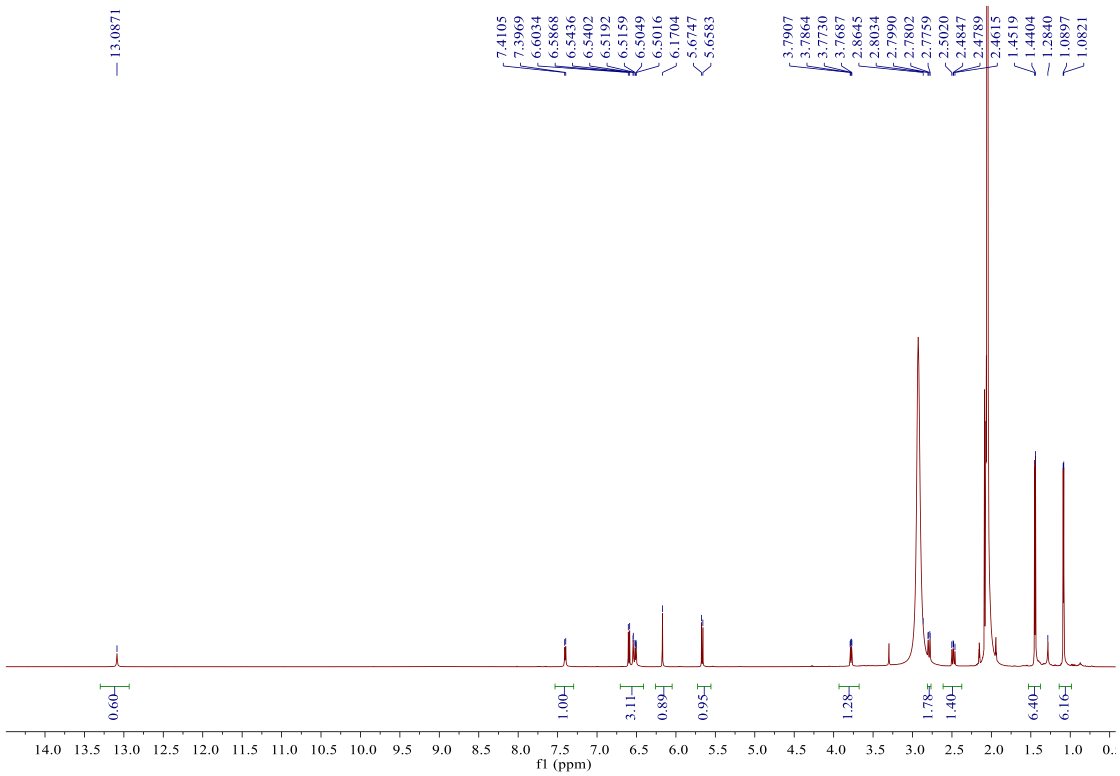

Figure S44. ^{1}H-NMR of compound **6**.

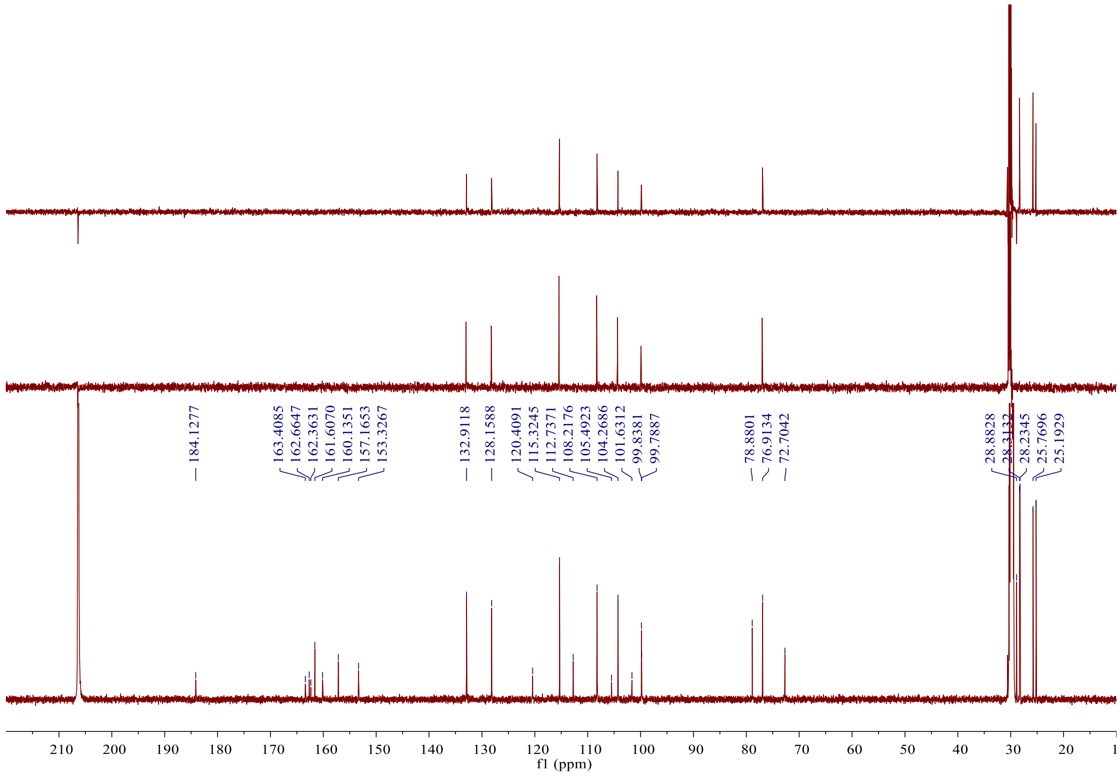

Figure S45. ^{13}C-NMR of compound **6**.

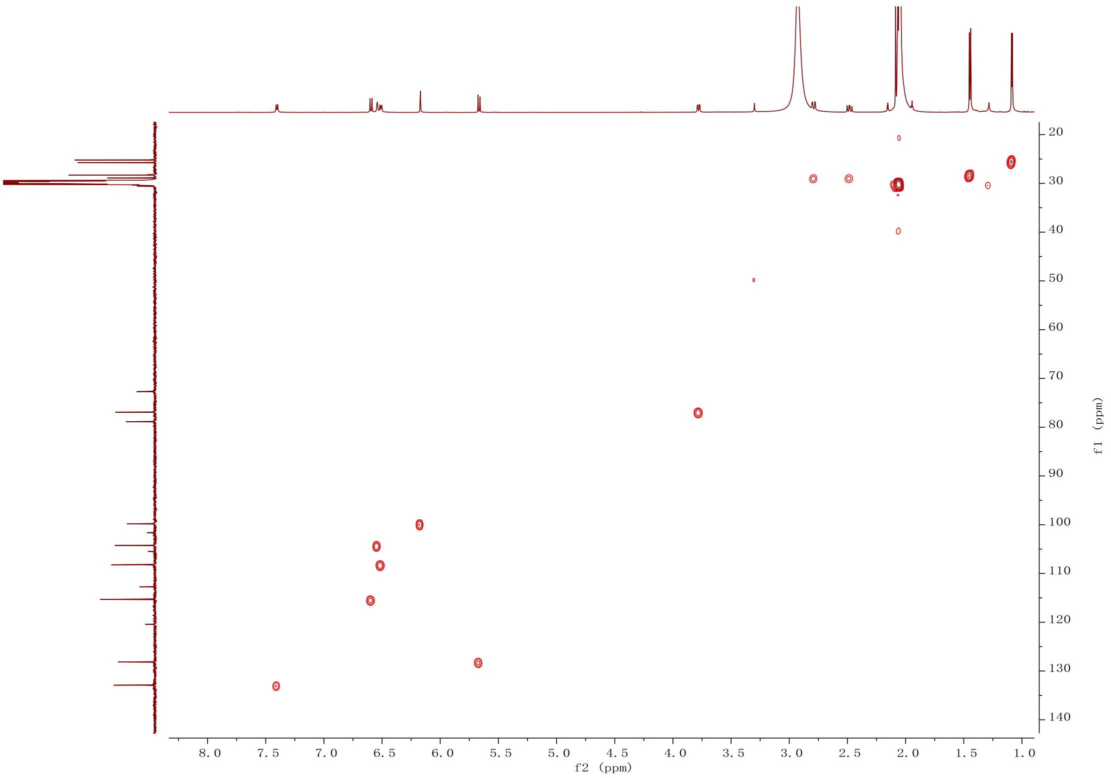

Figure S46. HSQC of compound **6.**

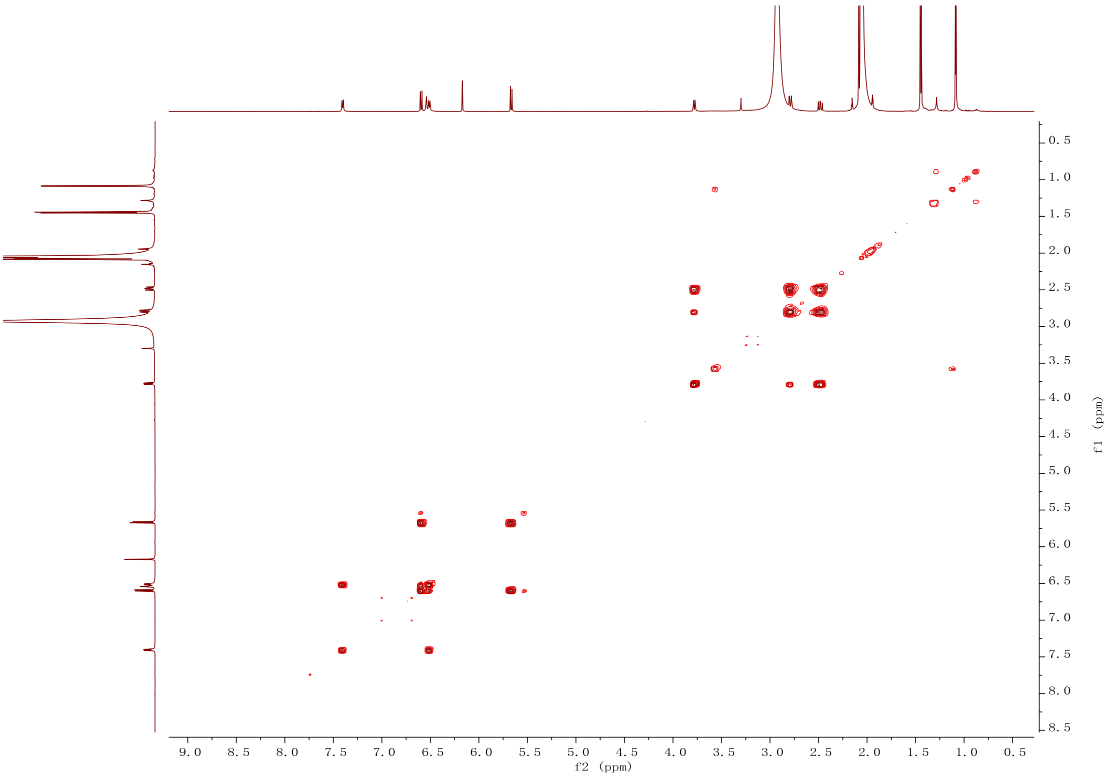

Figure S47. ^{1}H-^{1}H COSY of compound **6**.

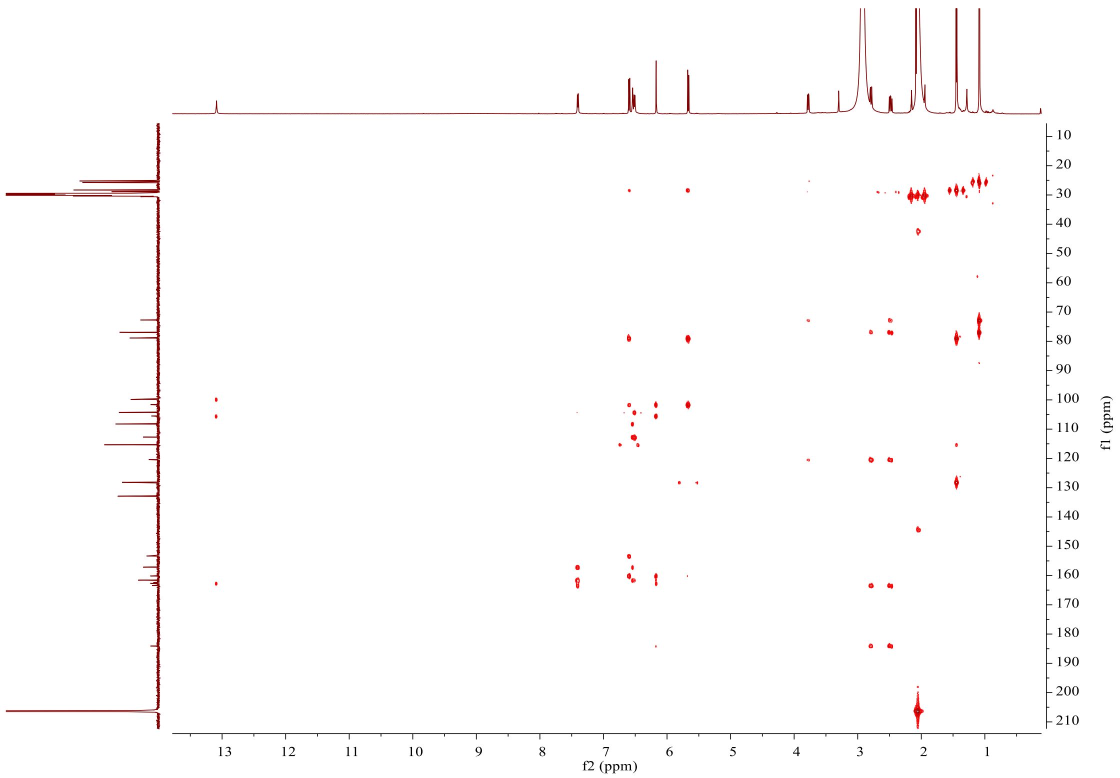

Figure S48. HMBC of compound **6**.

Qualitative Analysis Report

**Data Filename**	ZLZL-44.d	**Sample Name**	ZLZL-44
**Sample Type**	Sample	**Position**	P1-B6
**Instrument Name**	Instrument 1	**User Name**	
**Acq Method**	s-.m	**Acquired Time**	12/8/2021 3:18:54 PM
**IRM Calibration Status**	Success	**DA Method**	PCDL.m
**Comment**			

Sample Group **Info.**

Acquisition SW Version 6200 series TOF/6500 series Q-TOF B.05.01 (B5125.2)

User Spectra

Fragmentor Voltage	Collision Energy	Ionization Mode
135	0	ESI

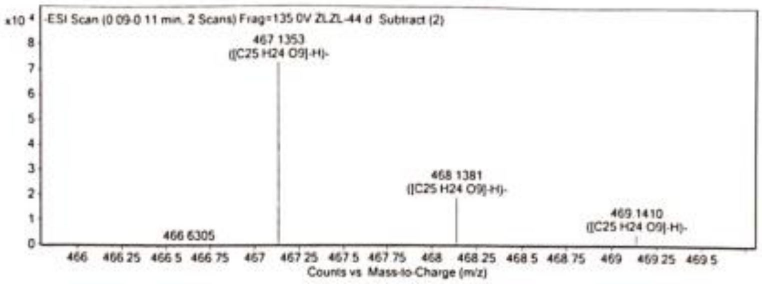

Peak List

m/z	z	Abund	Formula	Ion
403.3071	1	1944.01		
467.1353	1	72702.04	C25 H24 O9	(M-H)-
468.1381	1	19206.78	C25 H24 O9	(M-H)-
469.141	1	4629.53	C25 H24 O9	(M-H)-
503.1126	1	3532.65		
535.1228	1	8674.59		
536.1258	1	2351.45		
565.1127	1	6063.46		
587.094	1	3413.7		
603.1091	1	2698.43		

Formula Calculator Element Limits

Element	Min	Max
C	3	120
H	0	240
O	0	30

Formula Calculator Results

Formula	CalculatedMass	CalculatedMz	Mz	Diff. (mDa)	Diff. (ppm)	DBE
C25 H24 O9	468.1420	467.1348	467.1353	-0.50	-1.07	14.0000

--- End Of Report ---

Agilent Technologies Page 1 of 1 Printed at: 3:20 PM on: 12/8/2021

Figure S49. HRESIMS of compound **7**.

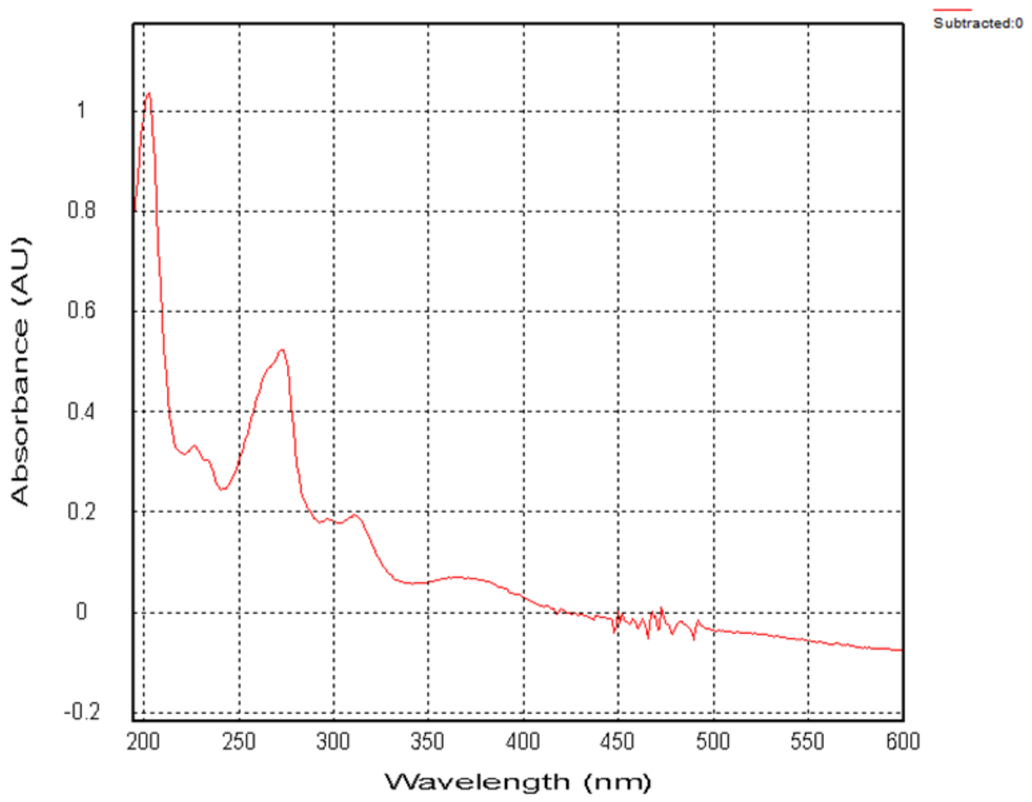

Figure S50. Ultraviolet spectra of compound **7.**

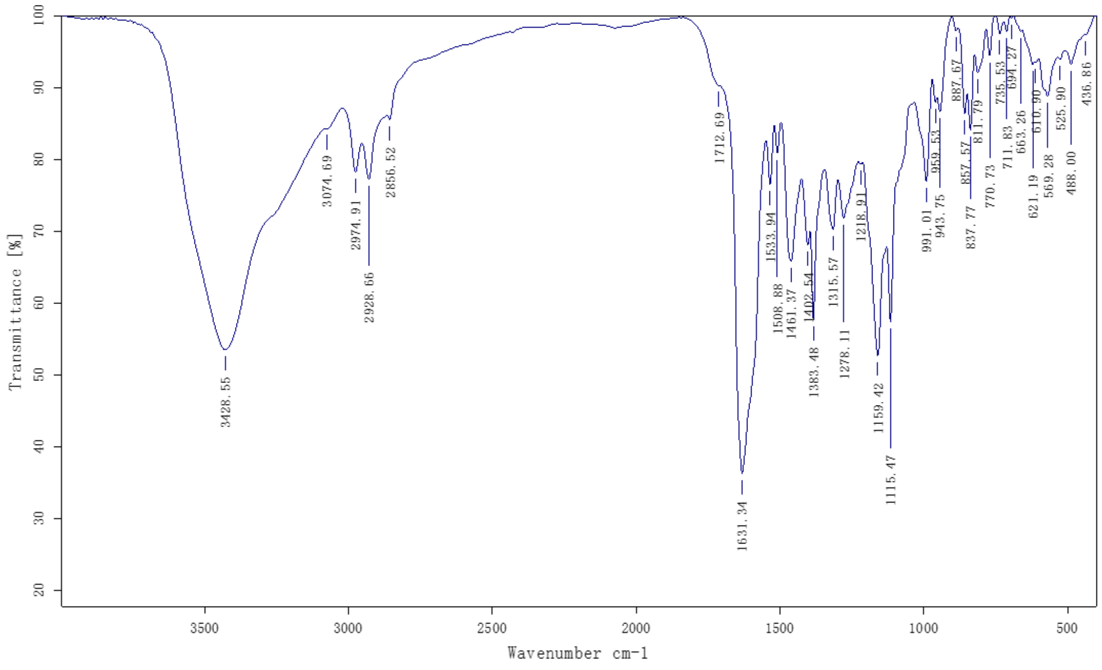

Figure S51. Infrared spectra of compound **7.**

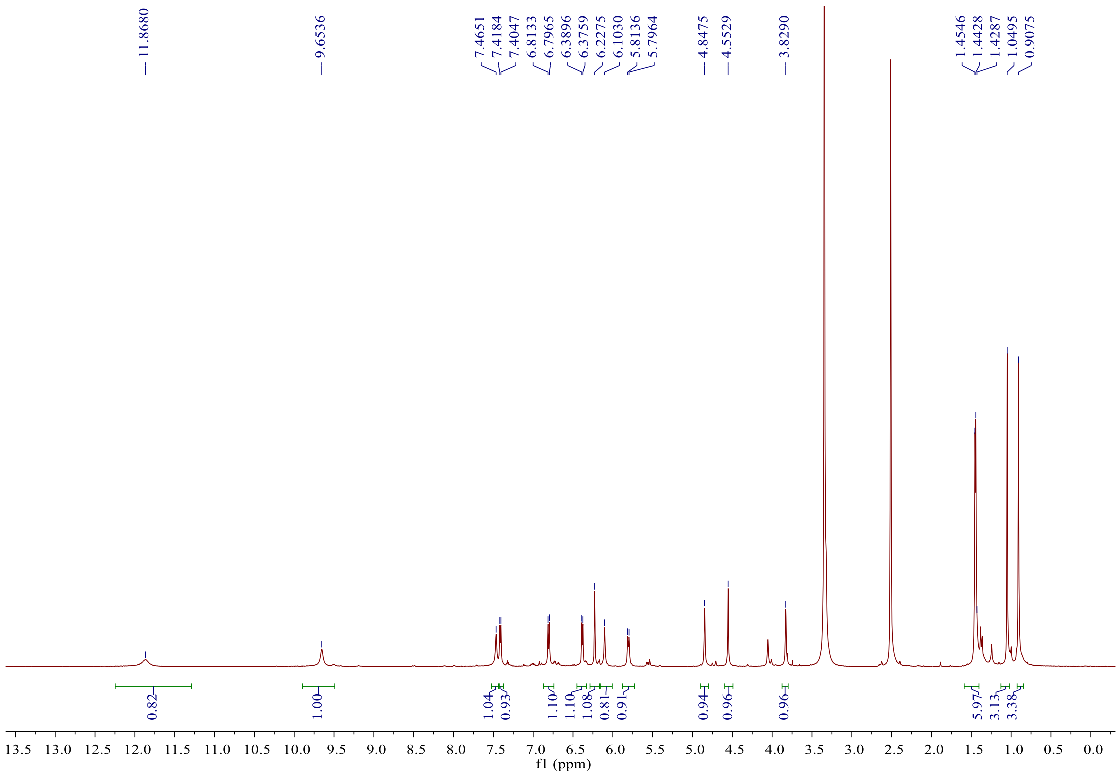

Figure S52. ^{1}H-NMR of compound **7**.

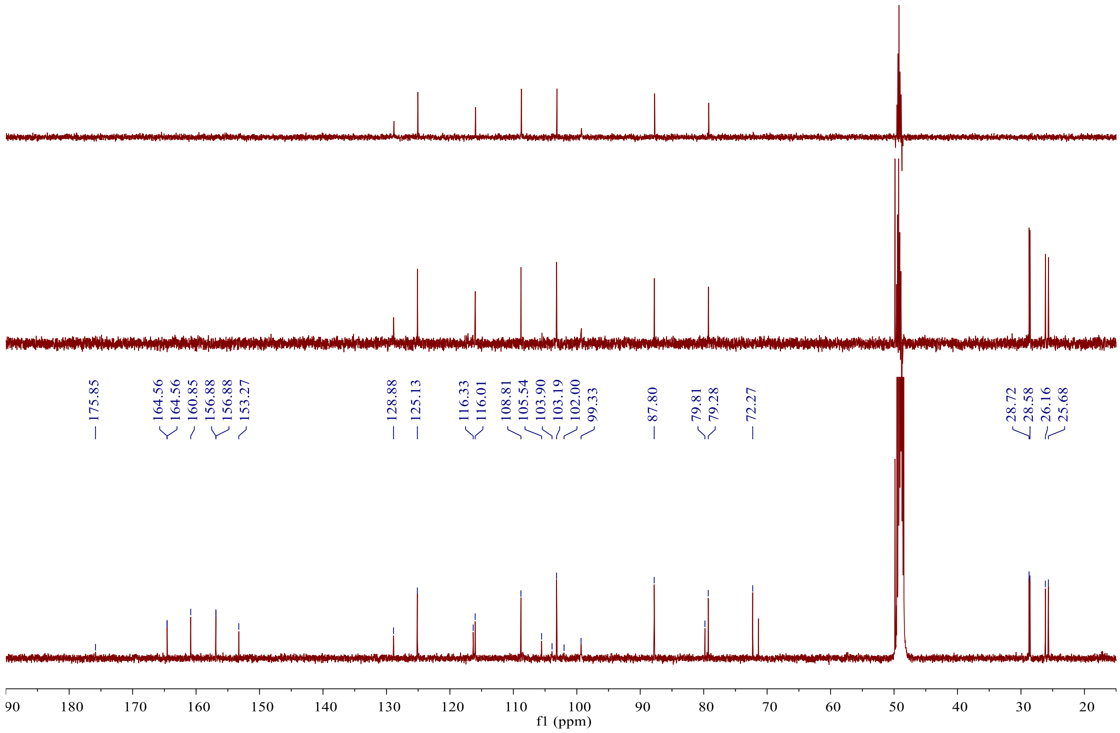

Figure S53. ^{13}C-NMR of compound **7**.

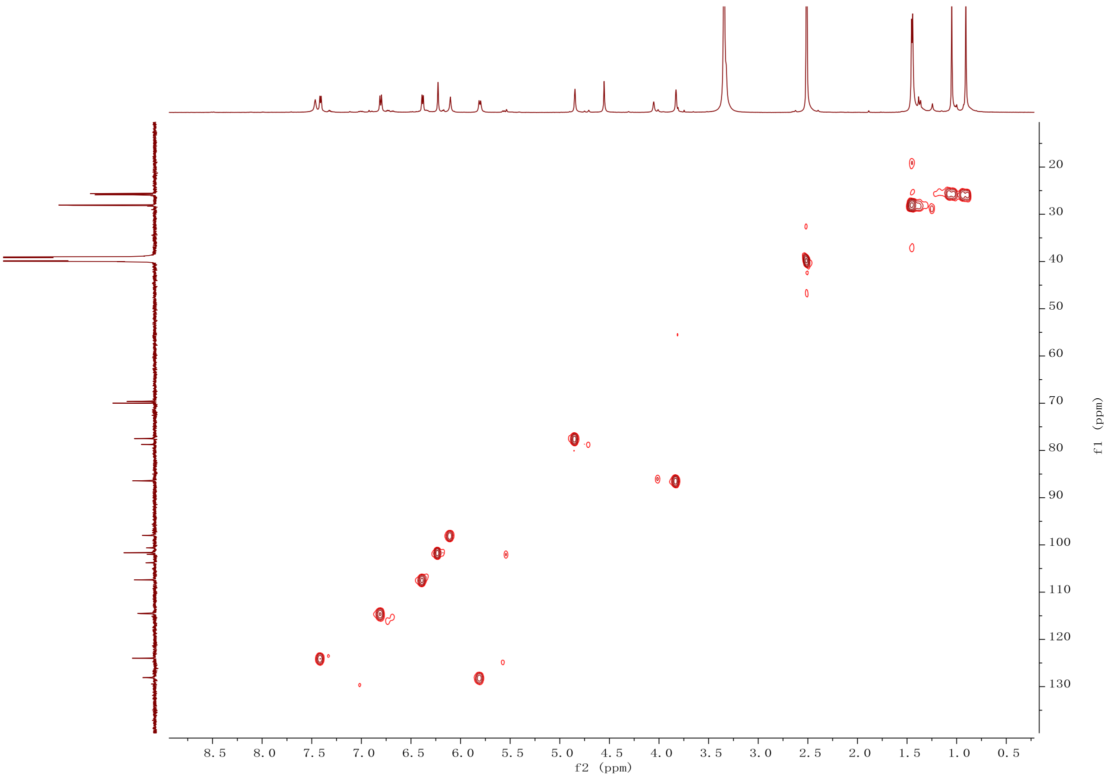

Figure S54. HSQC of compound **7.**

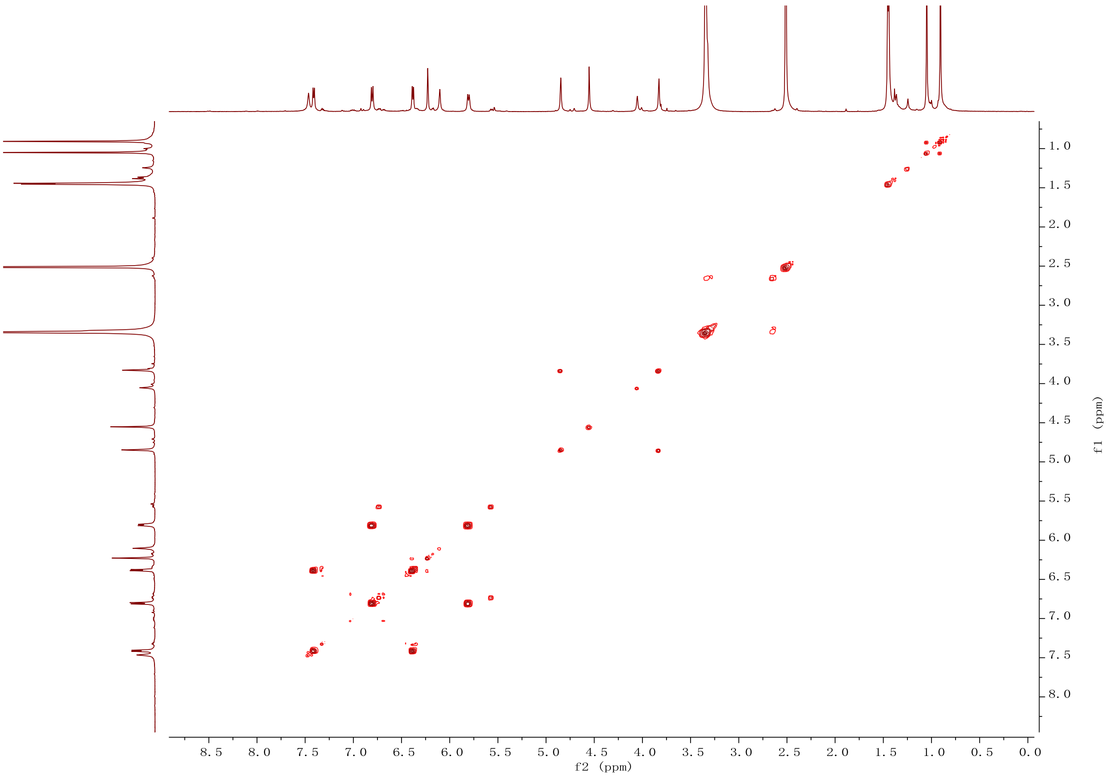

Figure S55. ^{1}H-^{1}H COSY of compound **7**.

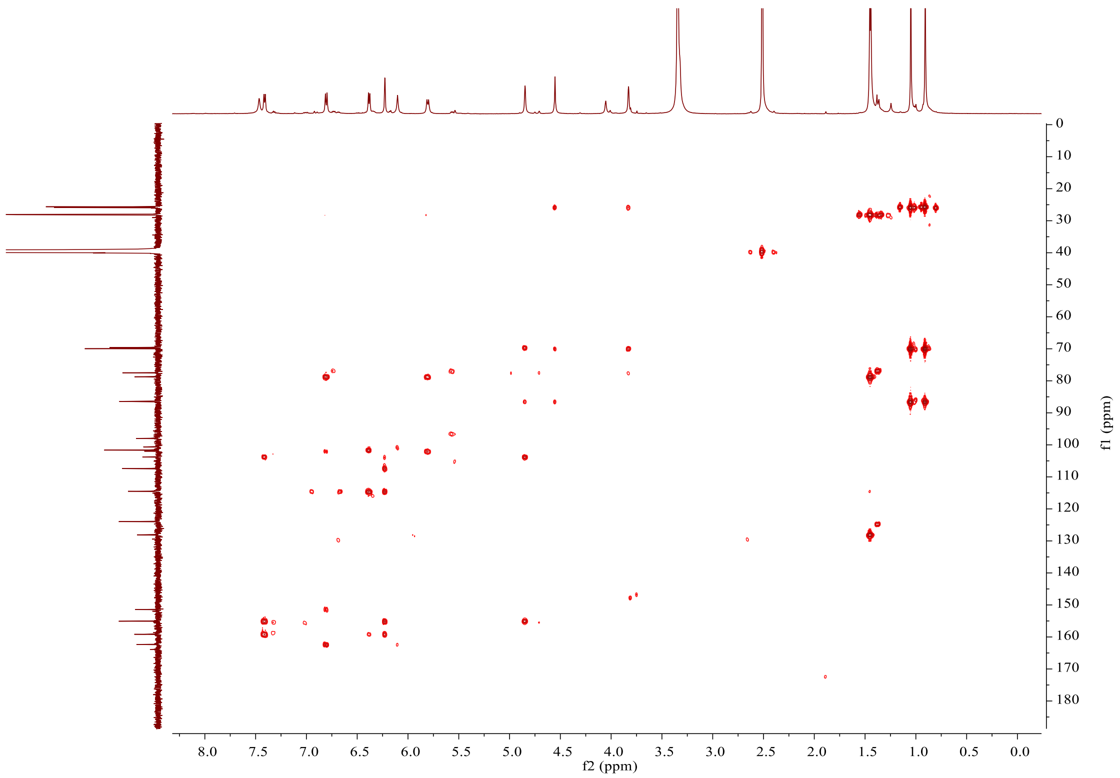

Figure S56. HMBC of compound **7**.

Qualitative Analysis Report

**Data Filename**	ZLZL-163.d	**Sample Name**	ZLZL-163
**Sample Type**	Sample	**Position**	P1-A2
**Instrument Name**	Instrument 1	**User Name**	
**Acq Method**	s-.m	**Acquired Time**	2/23/2022 4:51:21 PM
**IRM Calibration Status**	Success	**DA Method**	PCDL.m
**Comment**			

Sample Group **Info.**
Acquisition SW Version 6200 series TOF/6500 series Q-TOF B.05.01 (B5125.2)

User Spectra

Fragmentor Voltage	Collision Energy	Ionization Mode
135	0	ESI

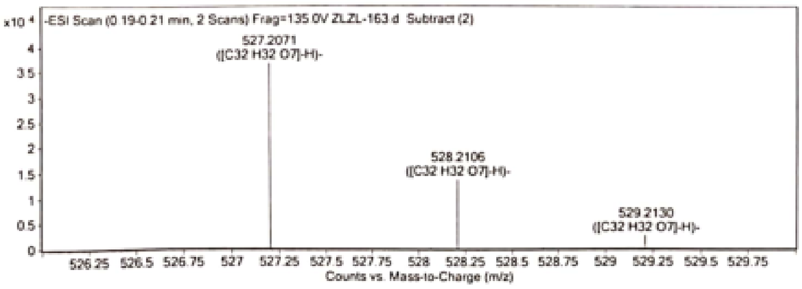

Peak List

m/z	z	Abund	Formula	Ion
355.3218	1	2441.46		
375.2748	1	2873.23		
403.3057	1	5781.05		
527.2071	1	36999.74	C32 H32 O7	(M-H)-
528.2106	1	13915.67	C32 H32 O7	(M-H)-
529.213	1	2644.26	C32 H32 O7	(M-H)-
590.2029	1	3904.25		
641.1994	1	51957.6		
642.2026	1	19168.59		
643.2055	1	4647.73		

Formula Calculator Element Limits

Element	Min	Max
C	3	60
H	0	240
O	0	30

Formula Calculator Results

Formula	CalculatedMass	CalculatedMz	Mz	Diff. (mDa)	Diff. (ppm)	DBE
C32 H32 O7	528.2148	527.2075	527.2071	0.40	0.76	17.0000

--- End Of Report ---

Agilent Technologies Page 1 of 1 Printed at: 3:35 PM on: 2/28/2022

Figure S57. HRESIMS of compound **8**.

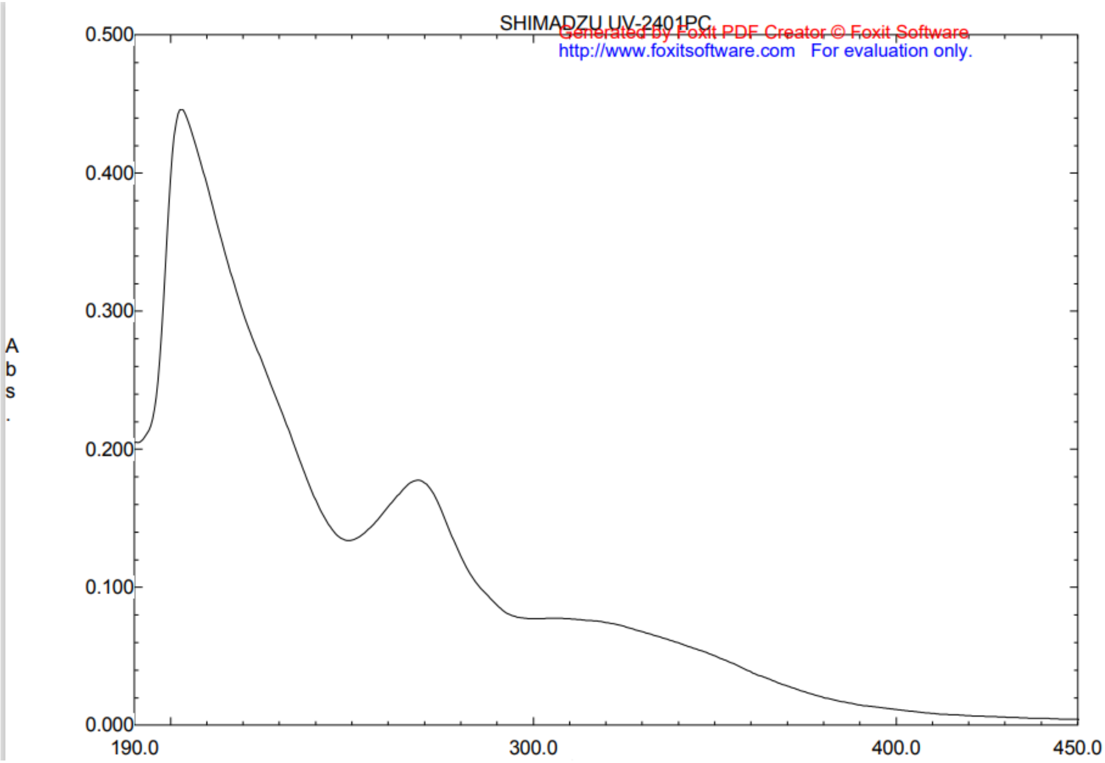

Figure S58. Ultraviolet spectra of compound **8.**

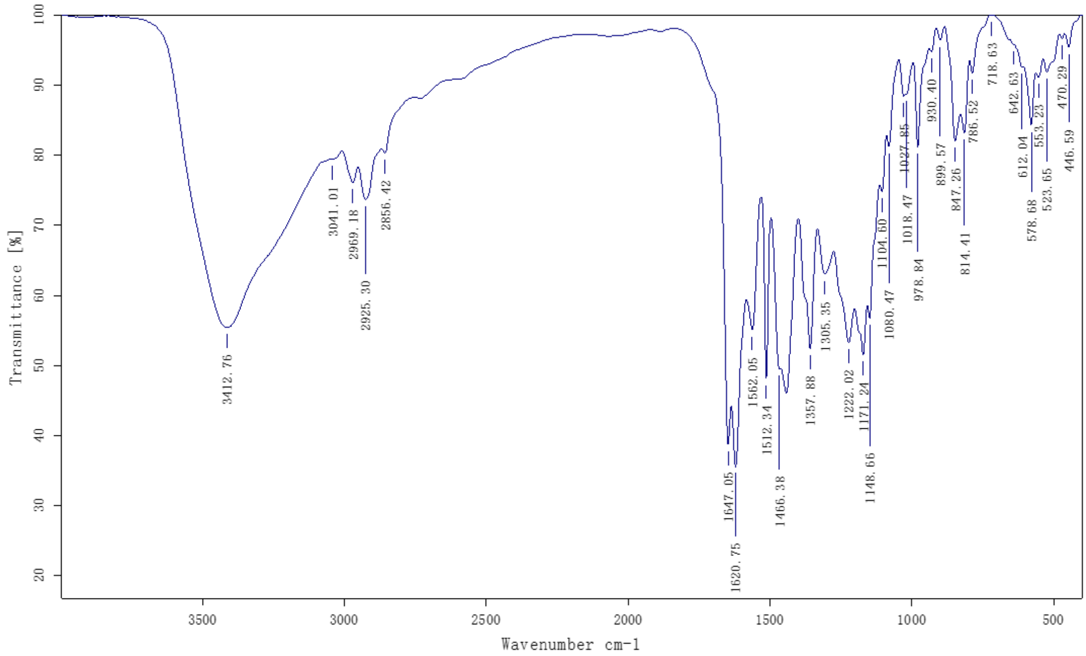

Figure S59. Infrared spectra of compound **8.**

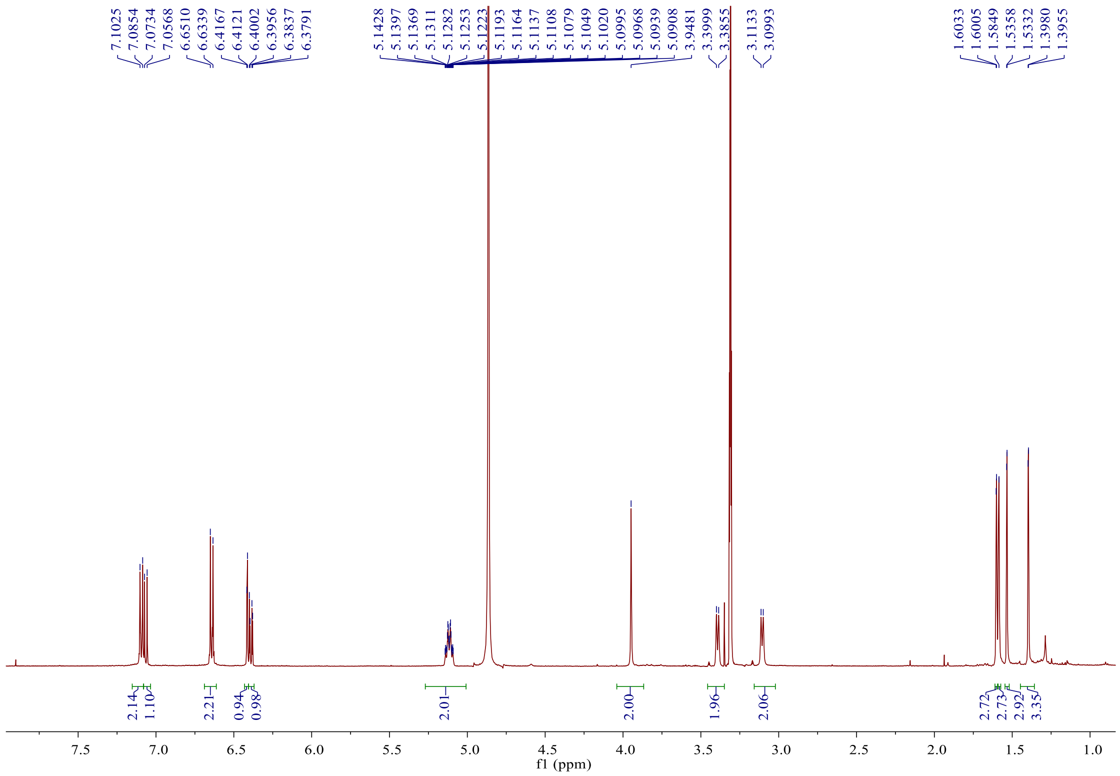

Figure S60. ^{1}H-NMR of compound **8**.

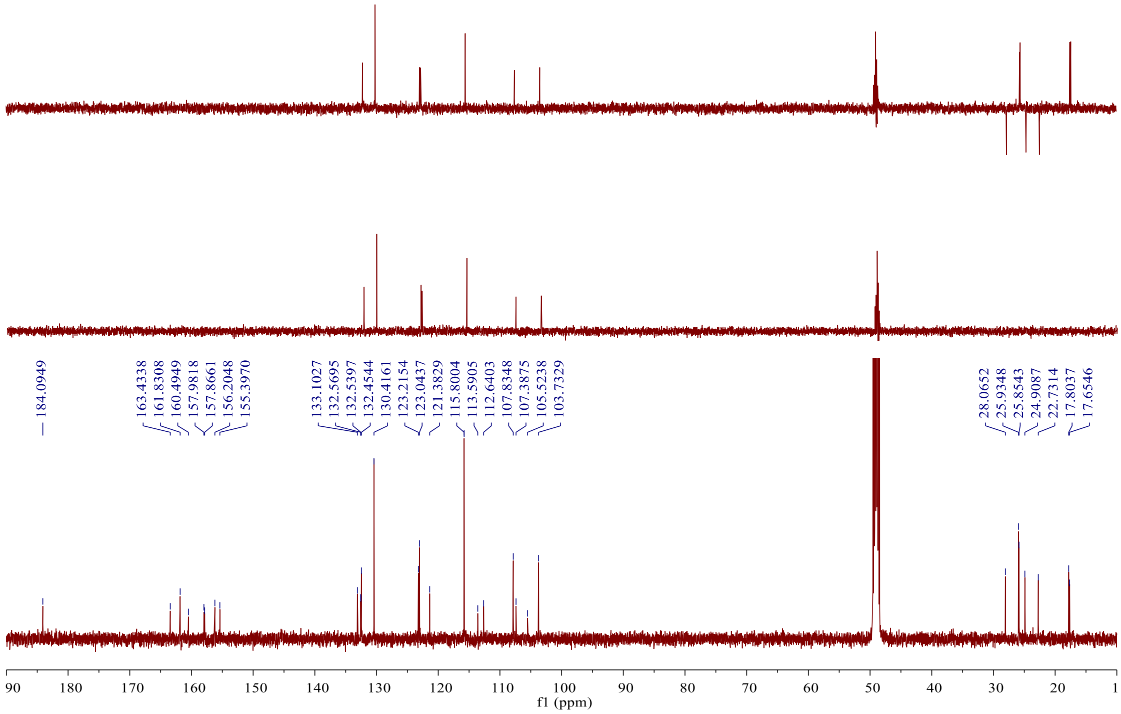

Figure S61. ^{13}C-NMR of compound **8**.

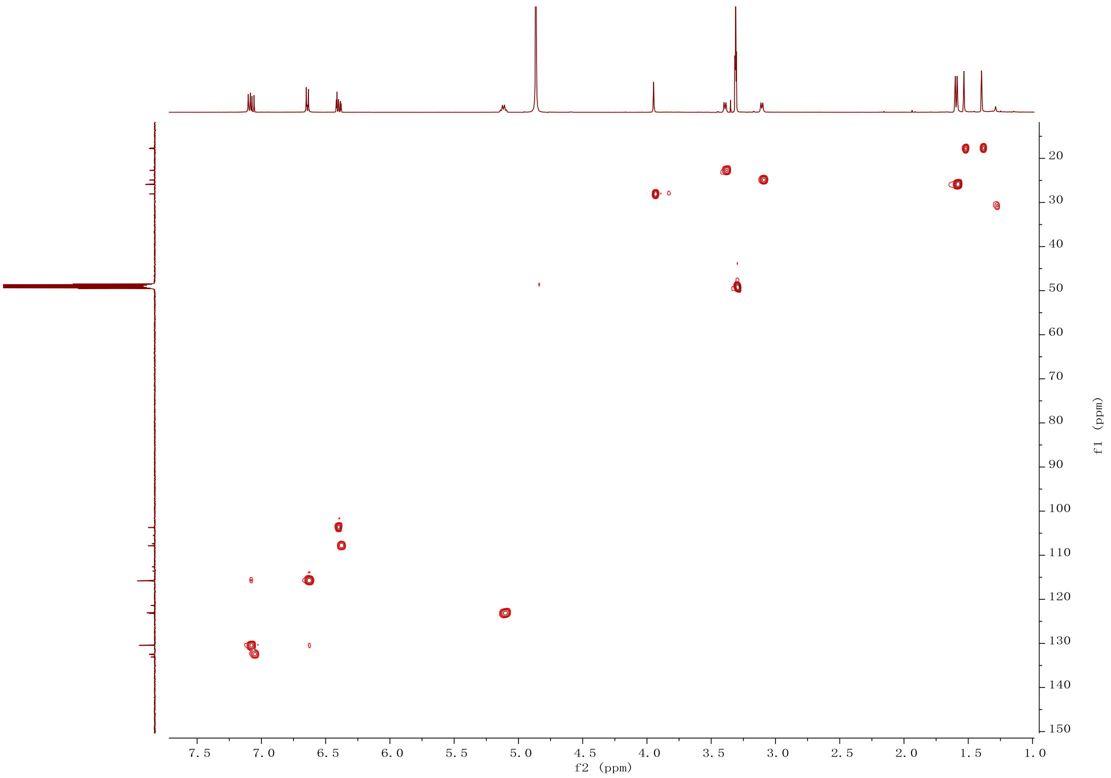

Figure S62. HSQC of compound **8.**

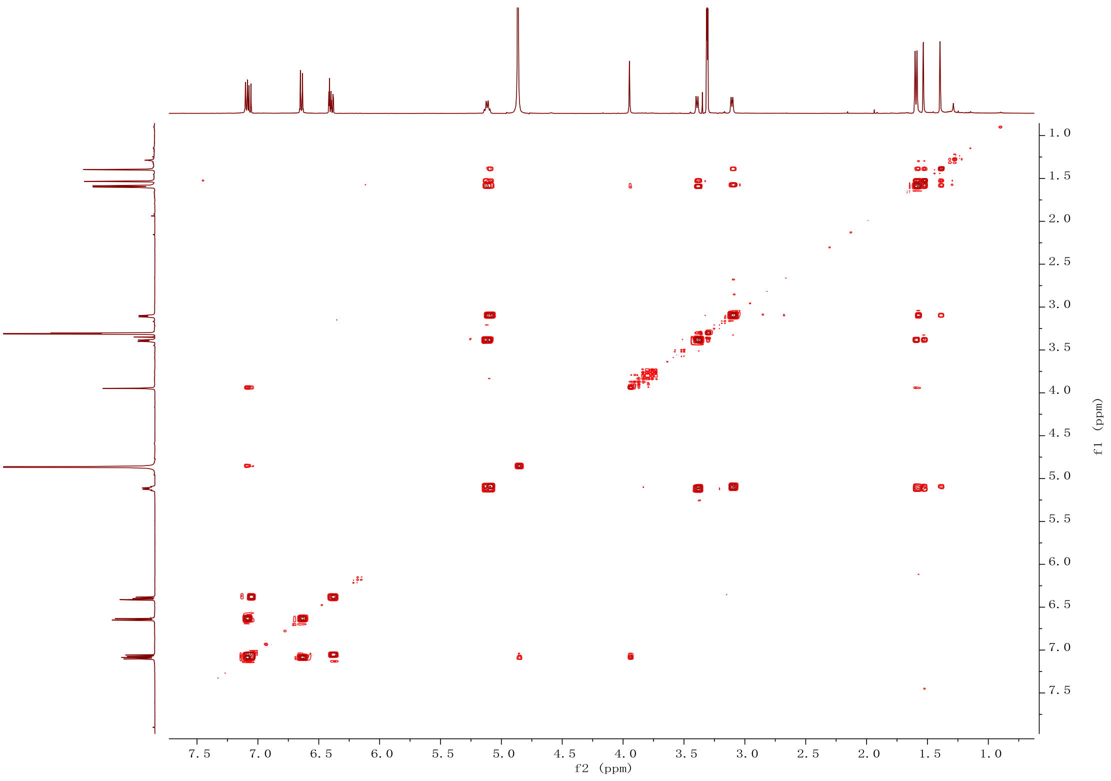

Figure S63. ^{1}H-^{1}H COSY of compound **8**.

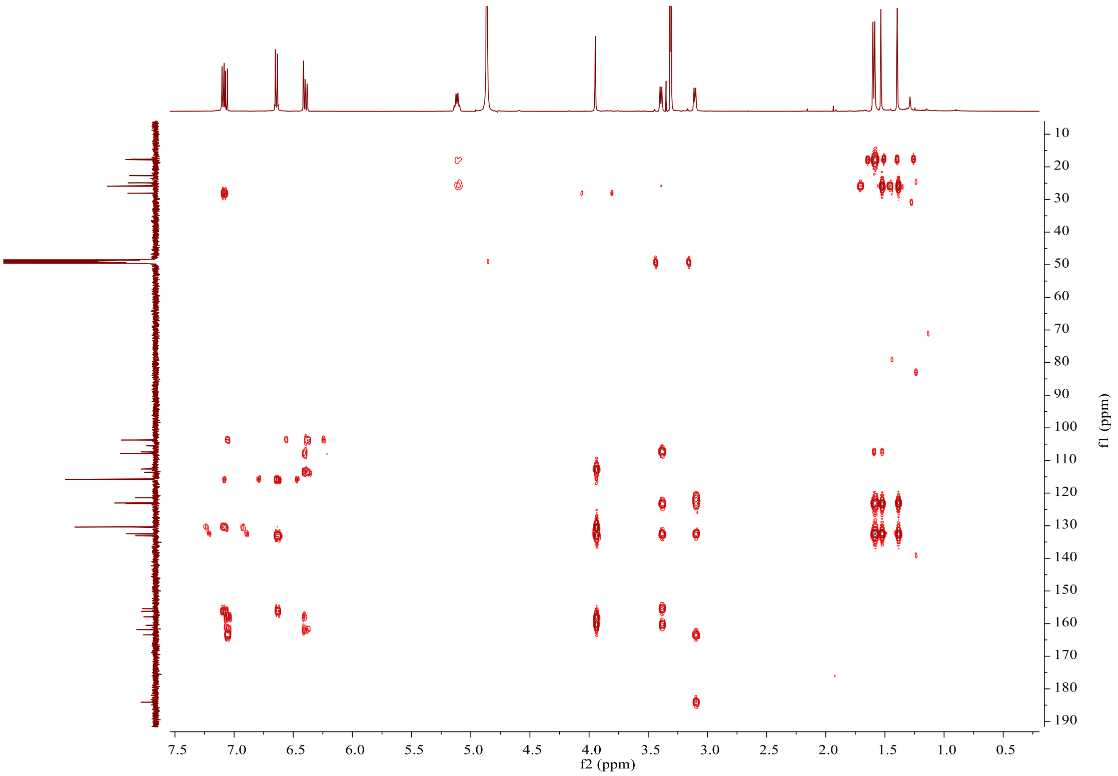

Figure S64. HMBC of compound **8**.

Qualitative Analysis Report

**Data Filename**	ZLZL-78+.d	**Sample Name**	ZLZL-78
**Sample Type**	Sample	**Position**	P1-A1
**Instrument Name**	Instrument 1	**User Name**	
**Acq Method**	s.m	**Acquired Time**	10/13/2021 11:03:19 AM
**IRM Calibration Status**	Success	**DA Method**	PCDL.m
**Comment**			

**Sample Group**		**Info.**
**Acquisition SW Version**	6200 series TOF/6500 series Q-TOF B.05.01 (B5125.2)	

User Spectra

Fragmentor Voltage	Collision Energy	Ionization Mode
135	0	ESI

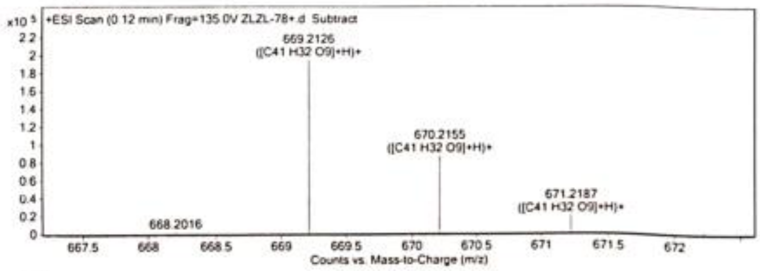

Peak List

m/z	z	Abund	Formula	Ion
101.0034		27252.34		
105.9814	1	24459.69		
137.0019	2	20800.98		
144.9879	1	14594.11		
225.1961	1	31604.95		
240.9875		15311.43		
256.9597	1	16341.07		
669.2126	1	194486.22	C41 H32 O9	(M+H)+
670.2155	1	87386.49	C41 H32 O9	(M+H)+
671.2187	1	19540.44	C41 H32 O9	(M+H)+

Formula Calculator Element Limits

Element	Min	Max
C	3	82
H	0	240
O	0	40

Formula Calculator Results

Formula	CalculatedMass	CalculatedMz	Mz	Diff. (mDa)	Diff. (ppm)	DBE
C41 H32 O9	668.2046	669.2119	669.2126	-0.70	-1.05	26.0000

--- End Of Report ---

Agilent Technologies Page 1 of 1 Printed at: 2:10 PM on: 10/13/2021

Figure S65. HRESIMS of compound **9**.

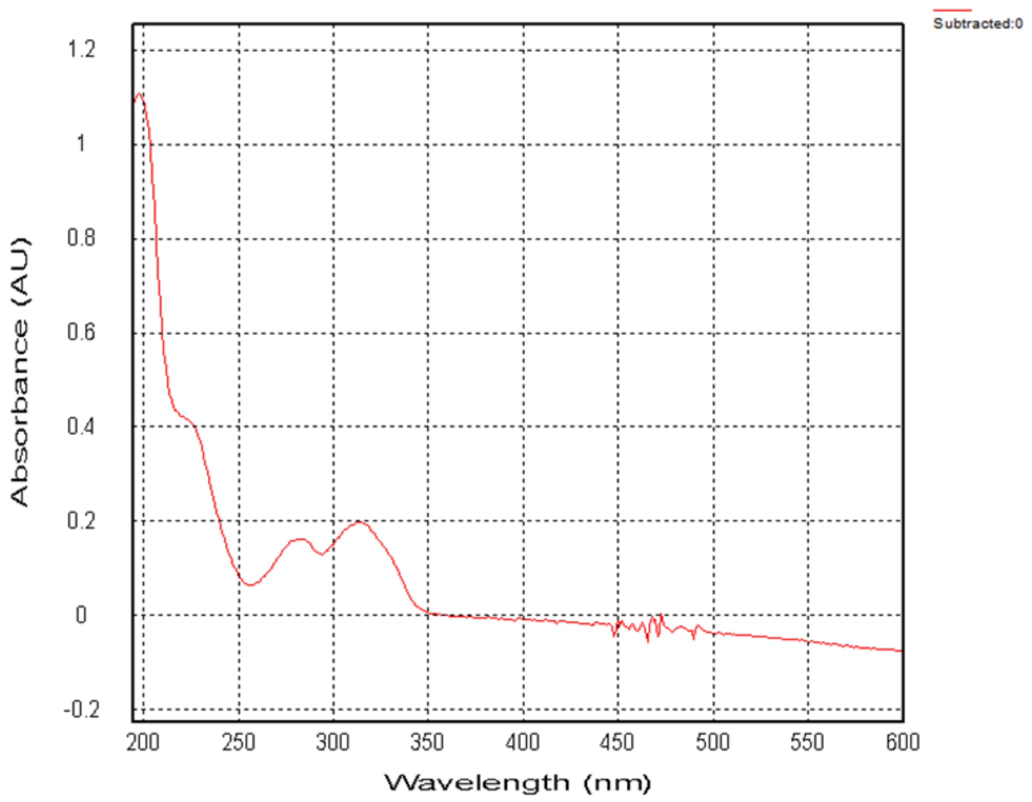

Figure S66. Ultraviolet spectra of compound **9.**

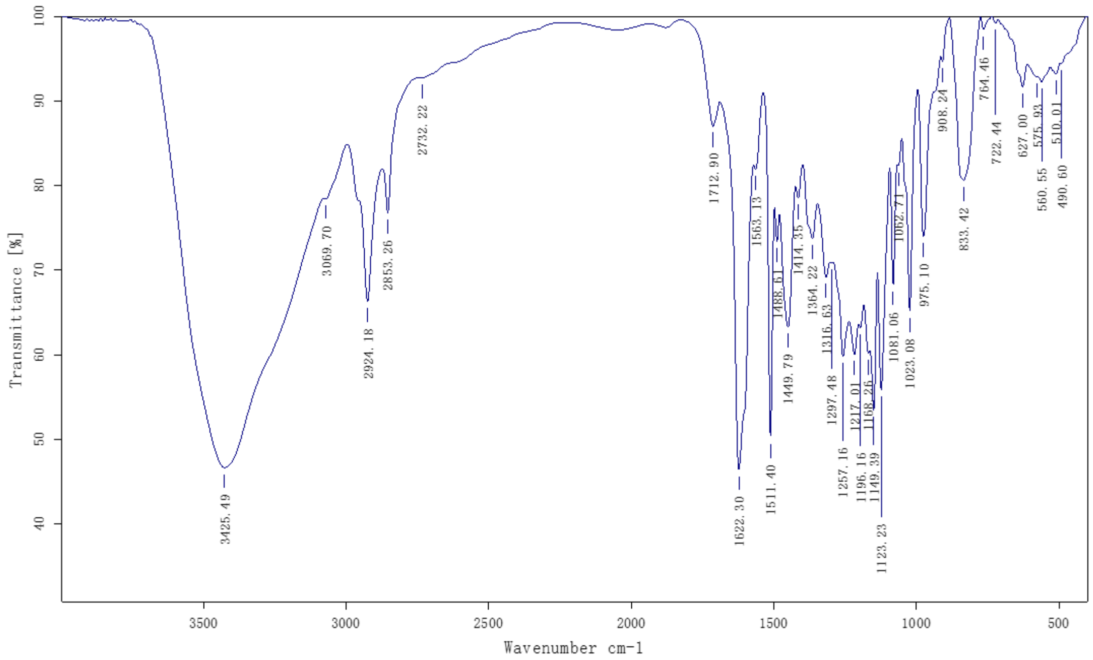

Figure S67. Infrared spectra of compound **9.**

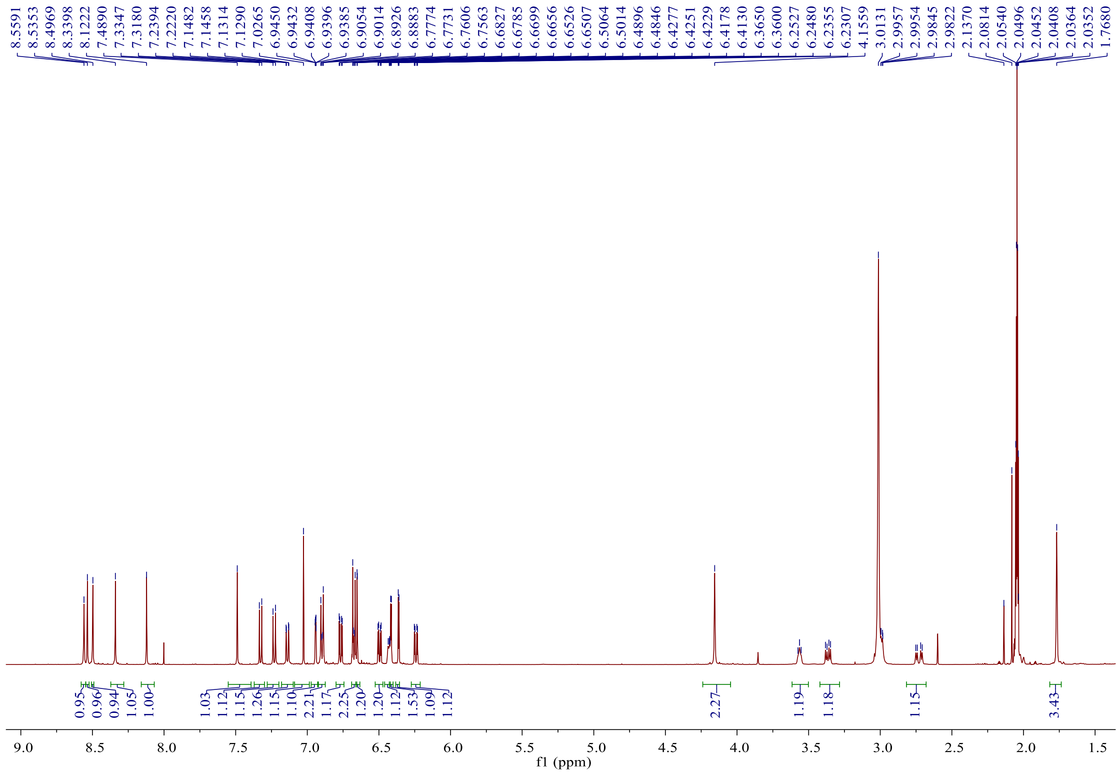

Figure S68. ^{1}H-NMR of compound **9**.

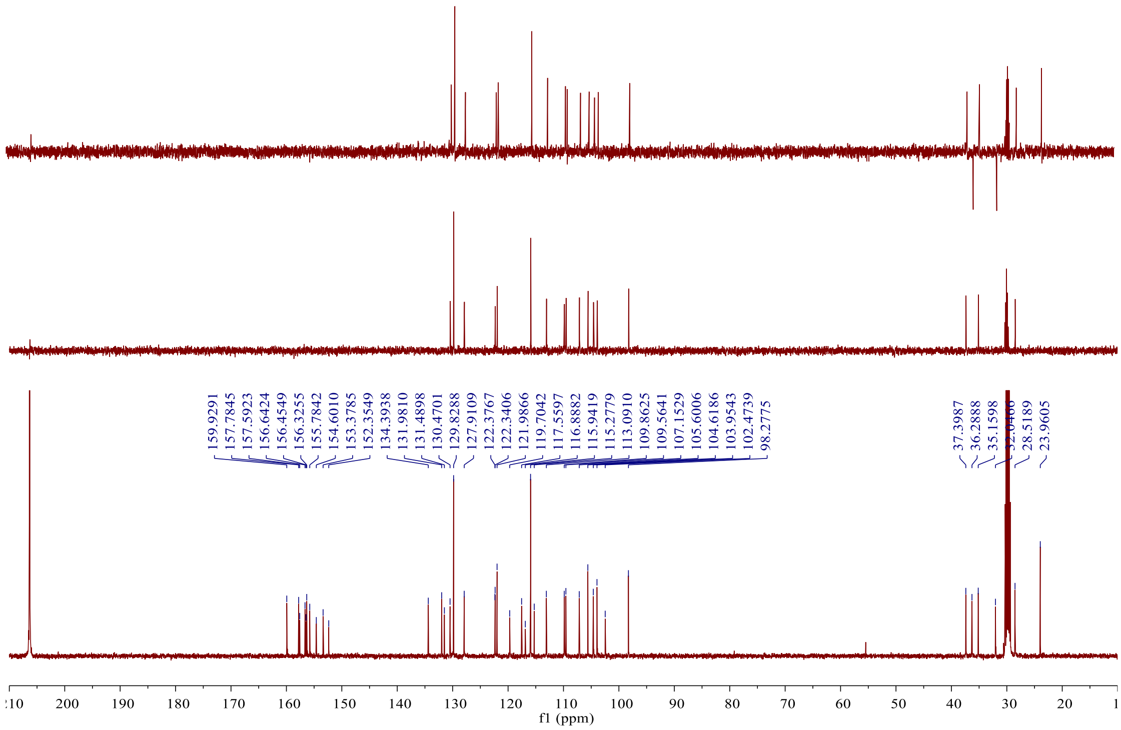

Figure S69. ^{13}C-NMR of compound **9**.

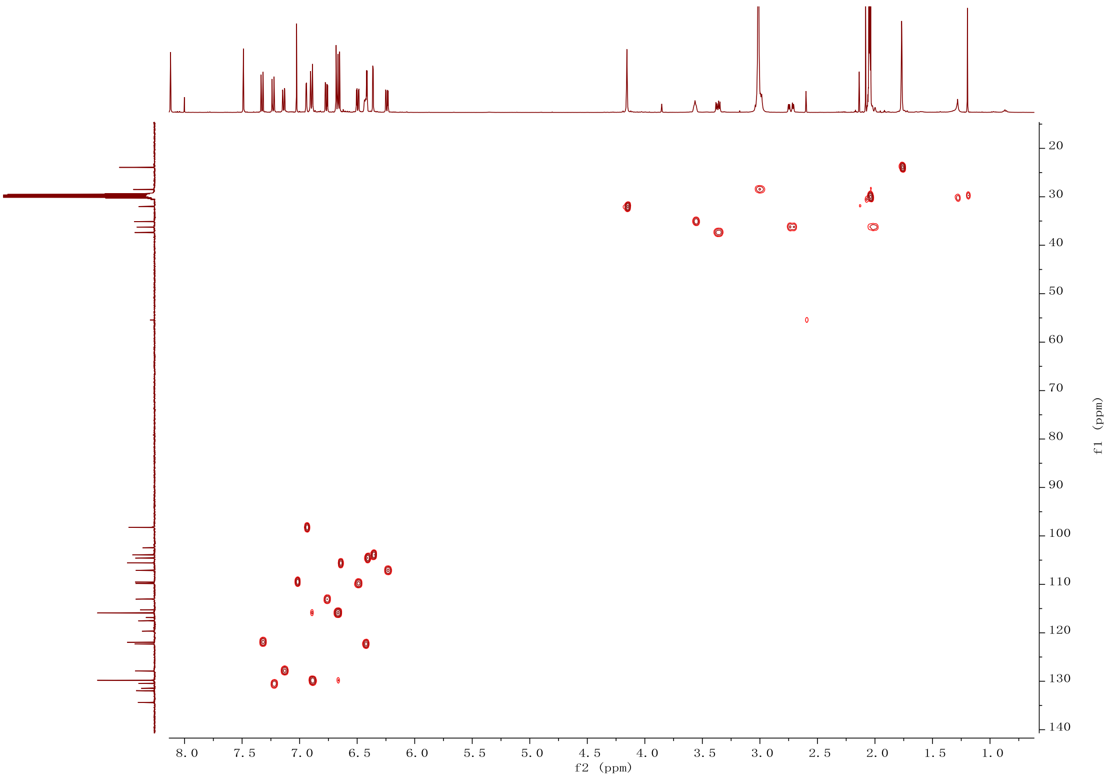

Figure S70. HSQC of compound **9.**

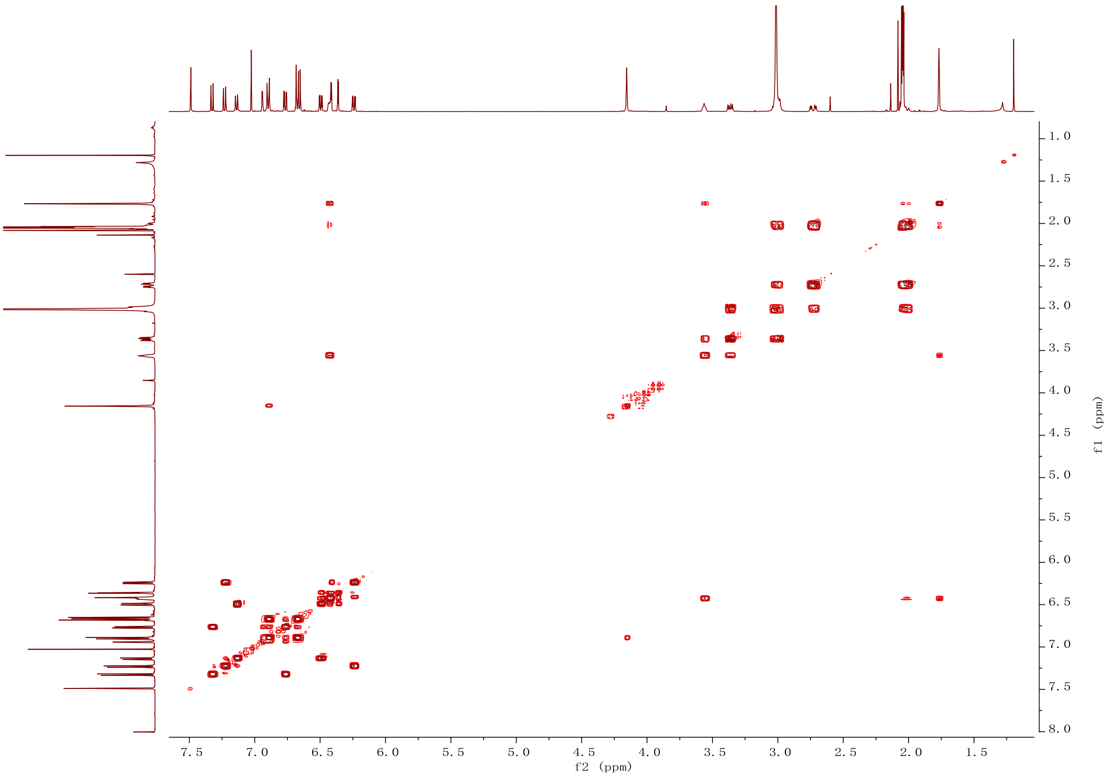

Figure S71. ^{1}H-^{1}H COSY of compound **9**.

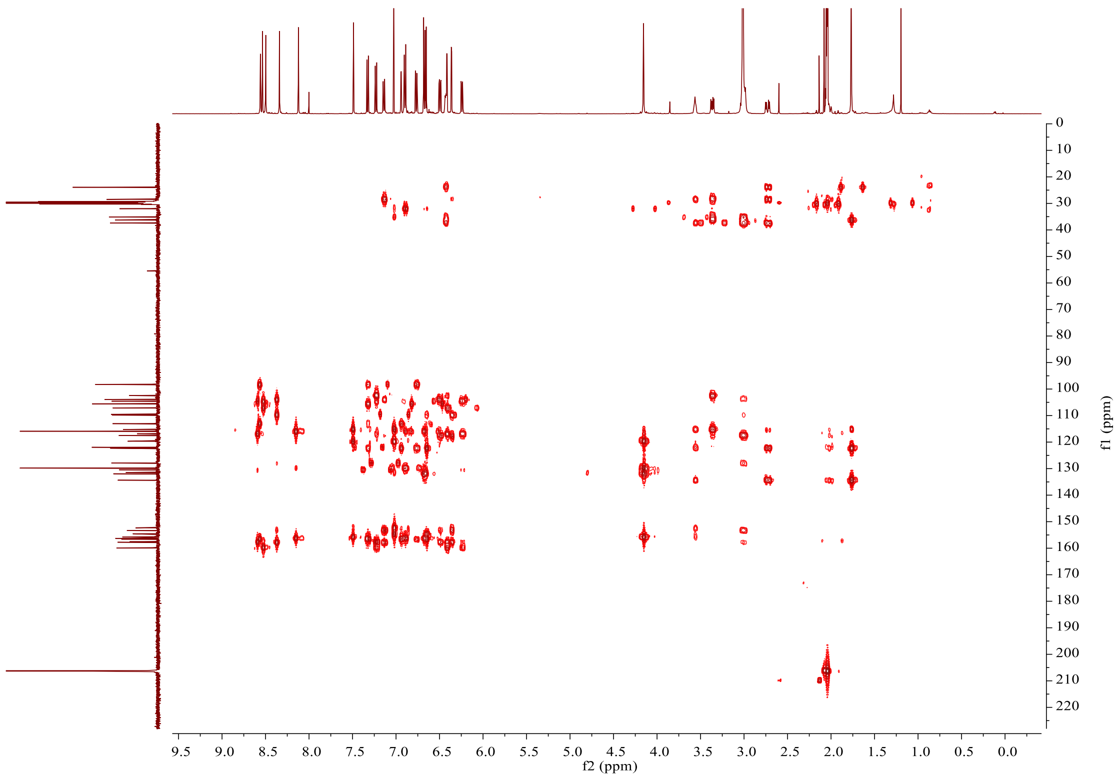

Figure S72. HMBC of compound **9**.

Qualitative Analysis Report

**Data Filename**	ZLZL-89.d	**Sample Name**	ZLZL-89
**Sample Type**	Sample	**Position**	P1-A1
**Instrument Name**	Instrument 1	**User Name**	
**Acq Method**	s.m	**Acquired Time**	10/22/2021 11:11:32 AM
**IRM Calibration Status**	Success	**DA Method**	PCDL.m
**Comment**			

Sample Group **Info.**
Acquisition SW Version 6200 series TOF/6500 series Q-TOF B.05.01 (B5125.2)

User Spectra

Fragmentor Voltage	Collision Energy	Ionization Mode
135	0	ESI

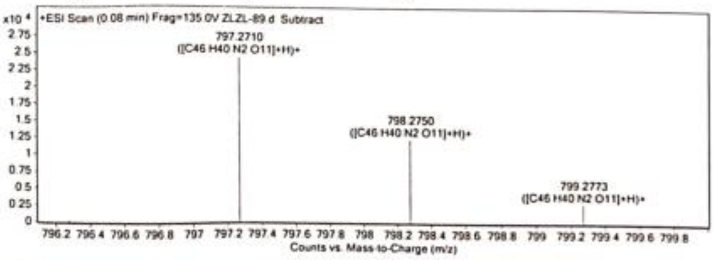

Peak List

m/z	z	Abund	Formula	Ion
79.0217	1	1609.11		
150.1124	1	3964.76		
163.9901	1	1543.67		
172.0944	1	1520.65		
223.1224	1	4983.22		
797.271	1	24322.06	C46 H40 N2 O11	(M+H)+
798.275	1	12399.62	C46 H40 N2 O11	(M+H)+
799.2773	1	2911.07	C46 H40 N2 O11	(M+H)+
819.254	1	4610.87		
820.2563	1	2781		

Formula Calculator Element Limits

Element	Min	Max
C	3	60
H	0	120
O	0	30
N	0	3

Formula Calculator Results

Formula	CalculatedMass	CalculatedMz	Mz	Diff. (mDa)	Diff. (ppm)	DBE
C46 H40 N2 O11	796.2632	797.2705	797.2710	-0.50	-0.63	28.0000

--- End Of Report ---

Figure S73. HRESIMS of compound **10**.

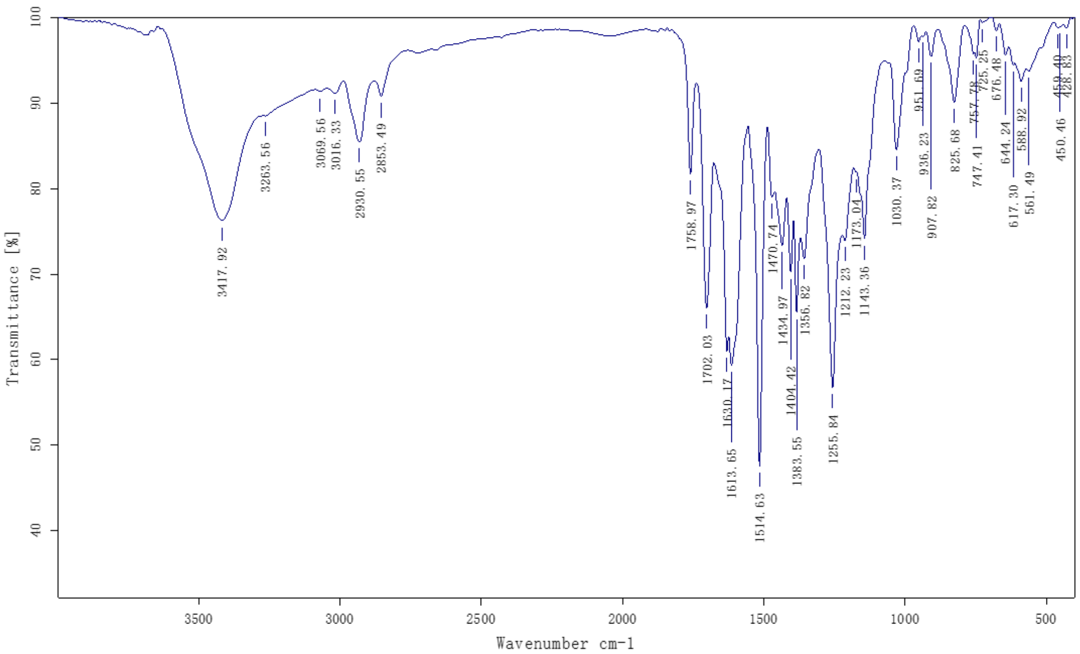

Figure S74. Infrared spectra of compound **10.**

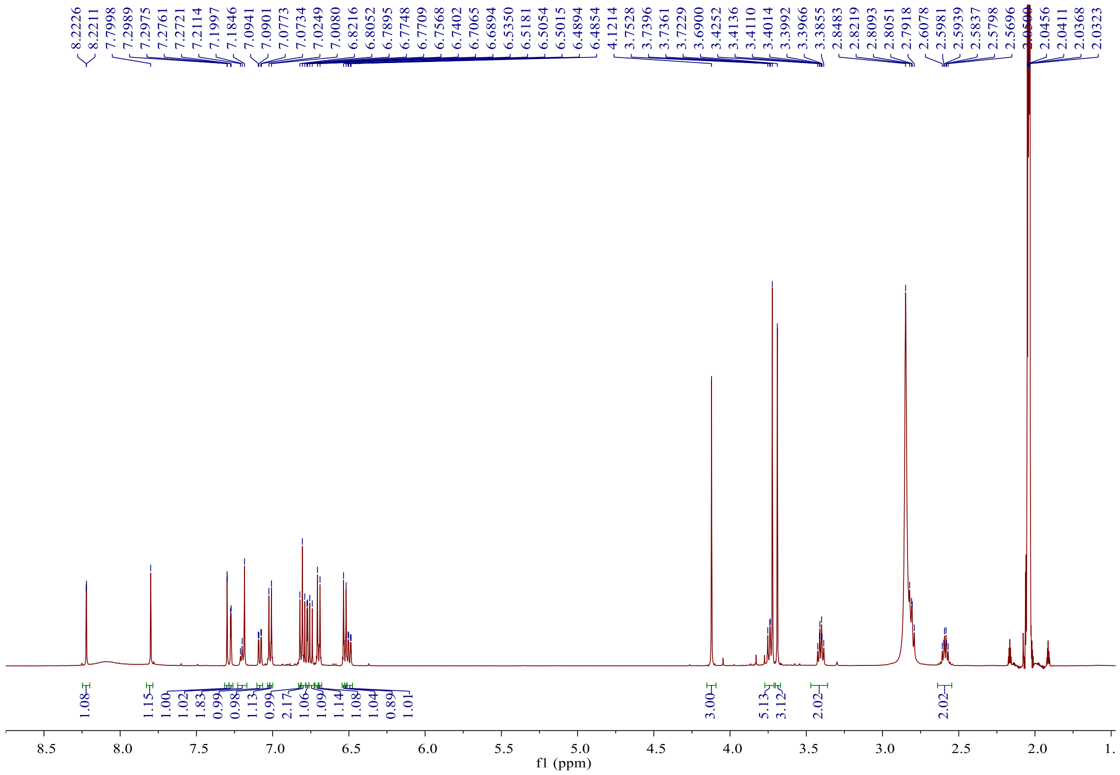

Figure S75. ^{1}H-NMR of compound **10**.

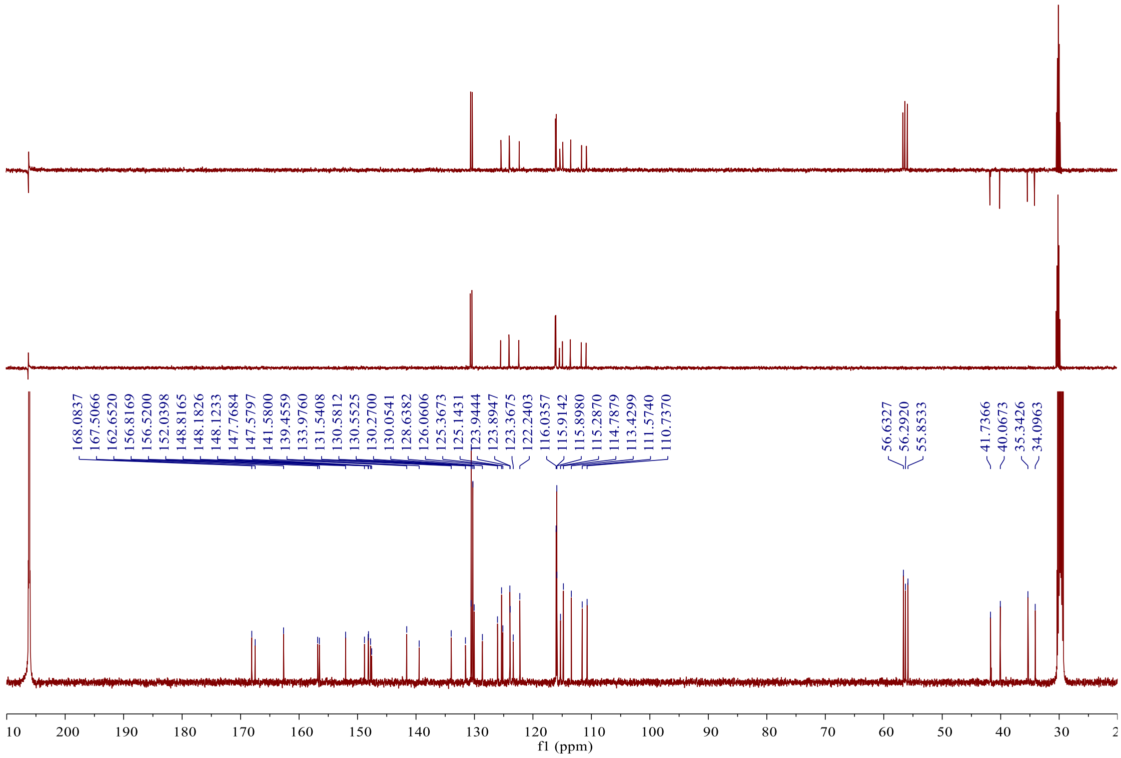

Figure S76. ^{13}C-NMR of compound **10**.

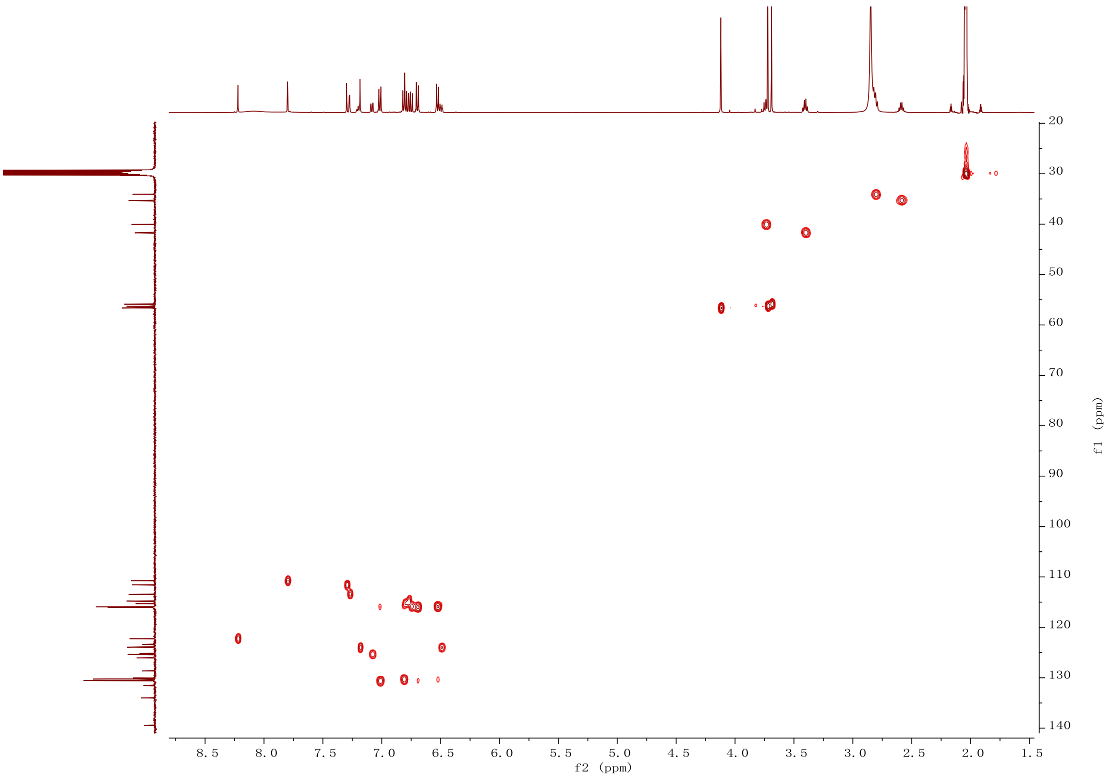

Figure S77. HSQC of compound **10.**

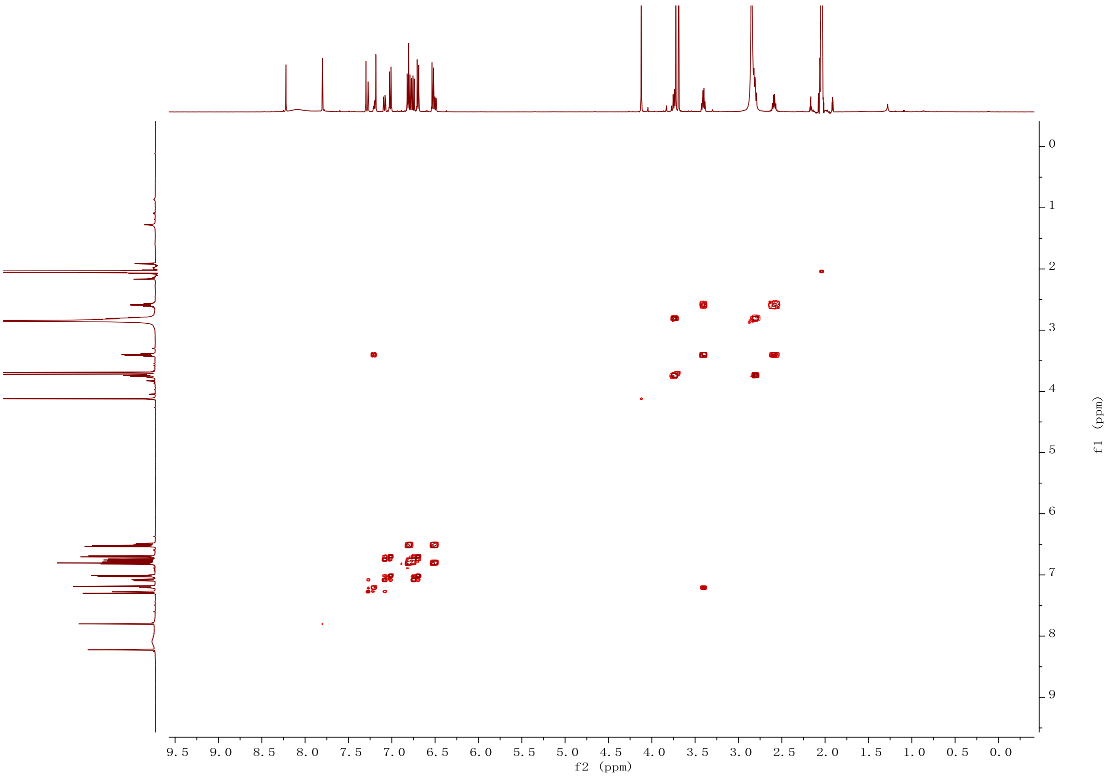

Figure S78. ^{1}H-^{1}H COSY of compound **10**.

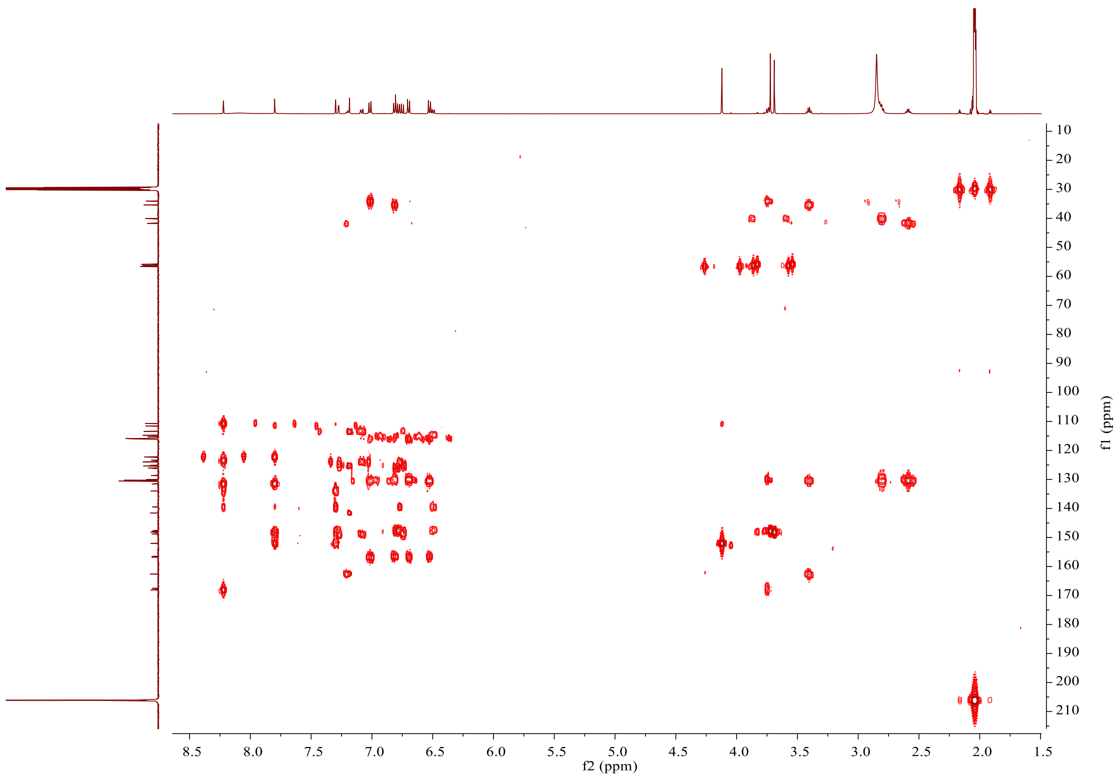

Figure S79. HMBC of compound **10**.
